# High-throughput antigen discovery using Functional Genomic Vaccinology (FGV) identifies protective *Streptococcus pneumoniae* vaccine candidates

**DOI:** 10.1038/s41467-026-75848-2

**Published:** 2026-07-21

**Authors:** Giuseppe Ercoli, Elisa Ramos-Sevillano, Samantha Palethorpe, Trisha Kerai, Timothy Scott, Rafael R. de Assis, Aarti Jain, Algis Jasinskas, Rie Nakajima, Jiin Felgner, Stephanie W. Lo, Brendan W. Wren, Philip Felgner, Jeremy S. Brown

**Affiliations:** 1 UCL Respiratory, University College London; Rayne Institute, London, UK; 2 Department of Infection Biology, London School of Hygiene and Tropical Medicine, London, UK; 3 Department of Physiology and Biophysics, Vaccine Research and Development Center, University of California, Irvine, Irvine, CA USA; 4 Parasites and Microbes, Wellcome Sanger Institute, Cambridge, UK

**Keywords:** Protein vaccines, High-throughput screening, Target identification, Protein vaccines

## Abstract

The discovery of protective antigens remains a major bottleneck in bacterial vaccine development. To overcome this limitation, we present Functional Genomic Vaccinology (FGV), a high-throughput antigen discovery platform integrating genome-wide antigen prediction, proteome-scale screening, and experimental immunogenicity validation to identify protective bacterial antigens. Using FGV, 222 conserved *S. pneumoniae* proteins are expressed in vitro, incorporated into a protein microarray, and coupled to magnetic beads for mouse vaccination. Protein array analysis shows significant IgG responses in 40% of the screened proteins. Antigen-specific responses measured in human sera guide the prioritisation of 22 candidates, which undergo further studied for their serological and Th17 responses. Four antigens combined in a multicomponent vaccine induces protection from pneumonia and sepsis in mice, with epitope mapping revealing potential protective sites for each protein. These results establish FGV as a scalable, experimentally driven approach for bacterial vaccine discovery and demonstrate its applicability in developing protective pneumococcal vaccines.

## Abstract (web summary)

To aid vaccine development against *Streptococcus pneumoniae*, the authors report a high-throughput antigen discovery platform integrating genome-wide antigen prediction, proteome-scale screening, and experimental immunogenicity validation.

## Introduction

Bacterial infections are estimated to cause 7.7 million deaths annually, yet at present there are no vaccines in clinical use for four of the top five bacterial causes of mortality, as well as many other important bacterial pathogens^1^. Vaccine development for these pathogens has been hindered for a variety of reasons, an important one of which is failure to identify suitable target antigens. For many bacterial pathogens, a vaccine that prevents infection with the majority of strains will need to target proteins with conserved expression across a species. However, due to the comparatively lower level of expression of protein compared to capsular polysaccharide antigens, and their localisation beneath the bacterial capsule, IgG to an individual protein antigen in general provides relatively weak protective immunity^2,3^. This can be overcome by creating multiantigen vaccines, promoting immunity through recognition of several surface structures^4^. Traditional reverse vaccinology has enabled genome-based identification of potential vaccine antigens, yet in many bacterial species, computational prediction alone fails to reliably identify proteins capable of inducing protective immunity^5^. More systematic approaches to antigen discovery include using phage display or protein microarrays to identify which proteins antibodies develop when humans or animals have been exposed to the whole pathogen, either through infection or whole cell vaccine approaches^6–10^. However, these approaches can only detect proteins that generate strong host antibody responses and will miss those that fail to induce measurable antibody levels upon bacterial exposure. To bridge this gap between prediction and protection, we developed a next-generation framework termed Functional Genomic Vaccinology (FGV). FGV unites genomic conservation analysis, proteomic expression, and functional immunogenicity screening into a single experimental continuum, directly linking a pathogen’s genetic repertoire to its capacity to elicit protective immune responses, thereby substantially increasing the pool of potential vaccine candidates and facilitating the development of multivalent vaccines.

FGV has been applied for the first time to the Gram-positive pathogen *Streptococcus pneumoniae* (or pneumococcus). *S. pneumoniae* causes pneumonia, septicaemia, and meningitis, and is the third most common cause of mortality due to bacterial infection and causes the highest level of years of life lost among bacterial pathogens^1^. *S. pneumoniae* has several advantages as a test pathogen, including (i) a relatively small genome encoding approximately 2200 proteins, (ii) established murine infection and vaccination models, and (iii) extensive background data from previous protein subunit or whole cell vaccine approaches and human serological studies to identify suitable proteins for screening and to cross-reference results^2,6,11–14^. Furthermore, although *S. pneumoniae* is the only one of the top five bacterial pathogens for which vaccines are in clinical use, these vaccines have major drawbacks that could be overcome by a multivalent protein subunit vaccine. Existing *S. pneumoniae* vaccines rely on purified capsular polysaccharide to induce protection either as polysaccharide alone (Pneumococcal Polysaccharide Vaccine) or conjugated to protein (Pneumococcal Conjugate Vaccine)^15^. Although these vaccines are highly effective^16^, they are both expensive and target only a proportion of the 100+ *S. pneumoniae* capsular serotypes, with particularly poor coverage of serotypes causing adult pneumonia^17–19^. They also provide minimal protection against serotype 3, an increasingly common cause of invasive infections globally^20^. Hence, there remains a strong need for a novel low-cost vaccine targeting protein antigens that protects against the majority of *S. pneumoniae* capsular serotypes for use in older adults or to boost protection for at-risk populations in low-and middle-income countries^15,21,22^.

In this study, we have developed FGV, an enhanced screening technology to identify antigenic proteins based on simultaneous immunisation of mice with multiple antigens and measuring antibody responses using a protein microarray. This integrative approach extends the principles of reverse vaccinology by coupling large-scale antigen discovery with quantitative experimental validation, enabling rational vaccine design across diverse bacterial pathogens. We have used this approach to identify which of 222 *S. pneumoniae* protein antigens induce significant antibody responses, and further down-selected from these to create a promising four-antigen multivalent vaccine formulation able to protect mice against sepsis and pneumonia.

## Results

### The design of an *S. pneumoniae* conserved protein microarray

**Fig. 1:**
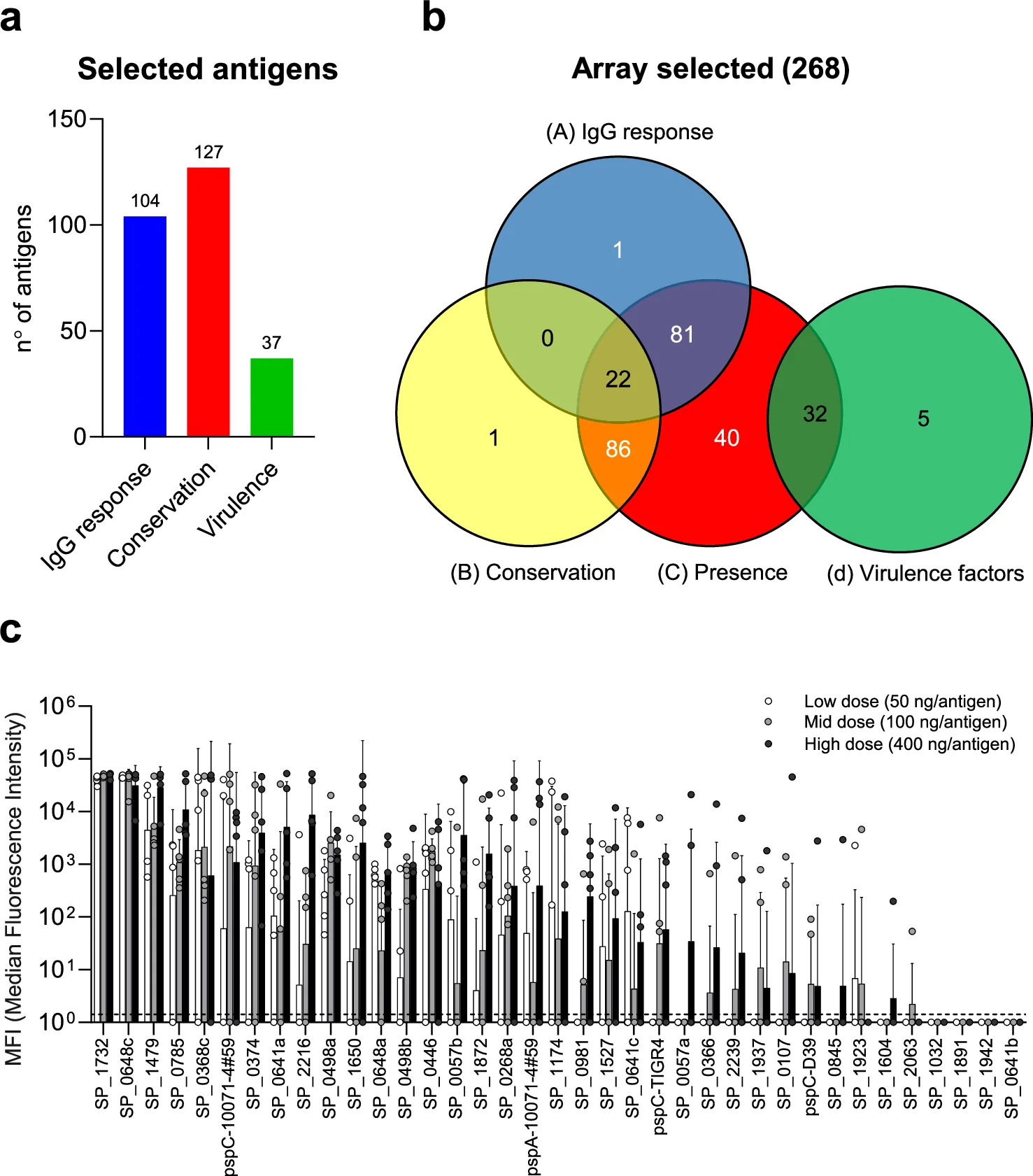
Protein microarray design. **a** Summary of the number of antigens selected for each criterion applied. **b** The number of antigens belonging to the different criteria used for the selection is reported. IgG response (N-IgG>1, blue), antigen conservation (πn < 0.002, yellow), presence (>97% of strains, red) across pneumococcal strains, and associated with virulence (green). **c** IgG response measured by protein array to CD1 mice sera immunised with 37 randomly selected bead-linked antigens (9–10 antigens combination per group, *n* = 5 mice/group, total *n* = 20). The whole panel of 37 antigens were tested in separate groups of mice using three different doses: 50 (white), 100 (grey) or 400 (black) ng per antigen per dose (total number of mice: 80). Bars represent average MFI/ group of mice, error bars standard deviation (SD) and dots the individual mouse values.

A *S. pneumoniae* protein microarray containing 253 proteins was developed using reference genome data from 2048 conserved genes identified across 616 strains and including strains from 15 sequence clusters and expressing 32 capsular serotypes^23,24^. Protein immunogenicity was used as first criterion of selection, which was measured in the same study as normalised IgG binding (N-IgG) to human sera probing a whole genome *S. pneumoniae* protein array with healthy human sera from six unvaccinated individuals (with no history of proven pneumococcal infection) and two sources of pooled human immunoglobulin (known to contain IgG to multiple pneumococcal protein antigens induced by prior nasopharyngeal colonisation)^23,25^. Out of a total of 185 proteins identified (N-IgG>1), 104 were included in our protein array, prioritising the conserved ones (Fig. 1a, Supplementary Data 1—array selection). Protein conservation was calculated in the same study using nucleotide diversity (πn) and presence across all isolates (616 strains)^23^. 127 conserved proteins (πn < 0.002 and present in over 97% of strains, >600/616) were also included in our array selection (Fig. 1a, Supplementary Data 1—array selection). In addition, 37 less conserved proteins previously associated with pneumococcal virulence were included (Fig. 1a, Supplementary Data 1—array selection)^13,26,27^. Many proteins met more than one criterion, and the full breakdown is shown in Fig. 1b.

Proteins shorter than 80 amino acids were excluded, while those encoded by DNA sequences longer than 3000 bp were split into domains (11 proteins were divided into 24 peptides). Because PspA and PspC antigens have been described to have different allelic variants^28,29^ we included four different sequences of each in the array. The sequences were selected after a phylogenetic analysis on gene sequences deriving from over 349 clinical isolates^30^ (Fig. S1), with the aim to try to cover the majority of the variability. The final selection was dominated by conserved proteins with significant IgG responses in human sera. This yielded a total of 288 array spots, corresponding to 268 unique proteins (255 single-domain proteins, 11 multi-domain proteins, and 2 proteins represented by four variants each).

The selected proteins for the microarray represented 18.6% (261/1399) of genes present in 97% of strains, with enrichment for proteins with highly conserved amino acid sequences (108/470, 23.0% of highly conserved proteins) and proteins recognised by IgG in human sera (104/221 antigens, 47.1%). The microarray was validated with sera from healthy humans (*n* = 6), intravenous immunoglobulins (Flebogamma and Vigam), and compared to data from previously tested pneumococcal colonised mice sera^10,31^. Protein-specific signal measured with our array was also compared with the results to those from Croucher et al.^23^. Proteins known to be recognised by human serum IgG that showed poor responses in our array were excluded, together with proteins spotted unevenly and with high background signal, yielding a final 253 protein microarray (Supplementary Data 1—array validation samples).

### Protein display on nickel beads and pilot vaccination experiments

To screen the selected *S. pneumoniae* proteins for their ability to induce antibody responses, the proteins included in the microarray were captured on nickel beads, purified and quantified by immunoblotting against the His-tag (Fig. S2). Of the 253 proteins present on the microarray, 222 were successfully linked to nickel beads; the remaining 31 antigens failed to be captured on the beads, mostly due to poor expression (Fig. S3). Groups of mice were vaccinated with combinations of nickel beads carrying 9–10 proteins with three intraperitoneal injections (with Addavax adjuvant) 2 weeks apart. The strength of IgG responses to each protein was assessed by probing the protein microarray with mouse sera obtained 21 days after the last vaccination. To help differentiate the strength of response between proteins, the doses used were markedly lower than usual for mouse vaccination studies using recombinant proteins. Initial dose finding studies performed for 37 randomly selected *S. pneumoniae* proteins tested 50 ng, 100 ng, or 400 ng per protein per vaccination on separate groups of mice (Supplementary Data 2). Results demonstrated comparable IgG responses to 50 and 100 ng/antigen doses (10/37 positives, 27%) but significantly higher responses to the 400 ng/antigen dose (20/37 positives, 54%) (Fig. 1c). IgG responses to individual proteins remained consistent when mice were re-vaccinated with the nickel beads reformatted to create pools containing different combinations of proteins (Fig. S4).

### Screening 222 *S. pneumoniae* proteins for their antigenicity

**Fig. 2:**
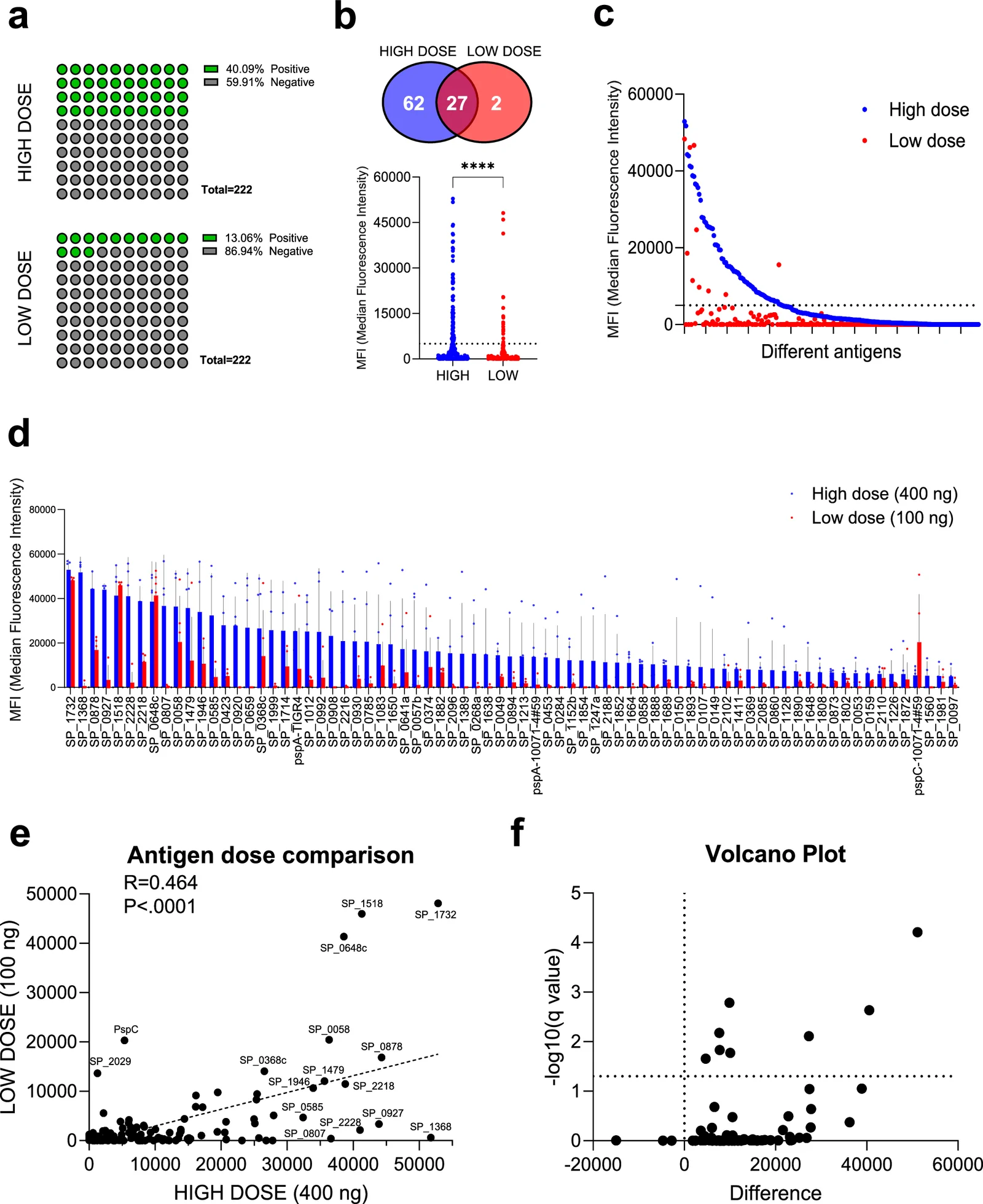
Protein microarray screening. **a** The percentage of antigens displaying specific IgG response in vaccinated mice sera measured by the protein array for both high (400 ng/antigen) and low dose (100 ng/antigen) screening (10–11 antigens per group, 5 mice/group, total mice *n* = 180). **b** The number of antigens positive for each dose tested (and common) is indicated in the Venn diagram (top) (blue, high dose - red, low dose). The median MFI measured for each antigen is reported for the two conditions, grouping all the values from the same dose (bottom) and **c** ranking the antigens from the top recognised to the lowest signal. Statistical significance was tested in (**b**) using two-tailed Mann–Whitney *U* tests (****p < 0.0001). **d** The average MFI signal measured for the top 75 proteins is reported (5 mice/group, *n* = 180), bars represent the average signal and dots the individual mouse values. Error bars represent the standard deviation. **e** A correlation between the response measured to individual antigens has been calculated for the two doses tested. Each dot represents an antigen. Correlation analyses were performed using Spearman’s rank correlation (two-tailed) to calculate the correlation coefficient (*R*) and *p* values that are reported in the top left corner. **f** Multiple *t*-tests were applied to compare the response obtained for each antigen in the two conditions, showing overall an increased response for the mice immunised with the high dose for the majority of antigens (right arm of the V). The dotted line indicates the threshold for statistical significance of the change.

The above experiments demonstrated that screening for protein antigenicity using the nickel bead approach produced consistent results. Hence, all 222 nickel bead-linked proteins were formatted into pools of 10 antigens and used to vaccinate mice (*n* = 5 per pool) on three occasions by intraperitoneal inoculation using both the 100 and 400 ng/protein dose (Supplementary Data 3). Probing the microarray with sera from vaccinated mice revealed specific IgG responses to a total of 91 proteins (41.0%), of which 89 (39.9%) proteins were positive after 400 ng dose vaccinations and 29 (13.1%) after 100 ng dose vaccinations (Fig. 2a, Supplementary Data 4—screening results). Positive antigens have been identified following the criteria described in the methodology section. For the majority of proteins that induced positive IgG responses, only a proportion of mice in each group had significantly raised IgG levels (Supplementary Data 4—screening results).

All proteins inducing IgG responses after low-dose screening were also positive in the high-dose screen, except two (SP_0498a and SP_2029) (Fig. 2b, Supplementary Data 4—candidates features). The median fluorescence intensity of IgG binding was higher in sera from mice vaccinated with the 400 ng dose for 23 of the 27 proteins with positive IgG responses in the 100 ng group. Three proteins (SP_1732, SP_1518, and SP_0648c) had similar MFIs for both the higher and lower dose vaccinations, suggesting these proteins were the most highly antigenic of the proteins investigated (Fig. 2c, d). Plotting the median IgG MFI for each protein according to the strength of responses showed close fitting to an exponential decay curve similar to human IgG data^23^, suggesting this vaccination dose had successfully identified all the proteins inducing significant IgG responses under these conditions (Fig. 2c). There were higher IgG MFIs after low-dose vaccination compared to high dose for one of the PspC alleles. The top 75 antigens ranked by the IgG response generated in the high-dose group are shown in Fig. 2d. When comparing the responses to the two doses, a strong correlation was observed in the antigens’ response (Fig. 2e), and a multiple *t*-test confirmed a statistically significant stronger response in the high-dose group for some of the antigens (Fig. 2f).

Antigen prediction software Vaxijen^32^ has been used to correlate the screening results with sequence-based predictions. A correlation matrix (Fig. S5A) shows no significant link between the predictions and the screening results.

### Proteins inducing IgG responses in mice significantly overlap with those inducing IgG responses in humans

**Fig. 3:**
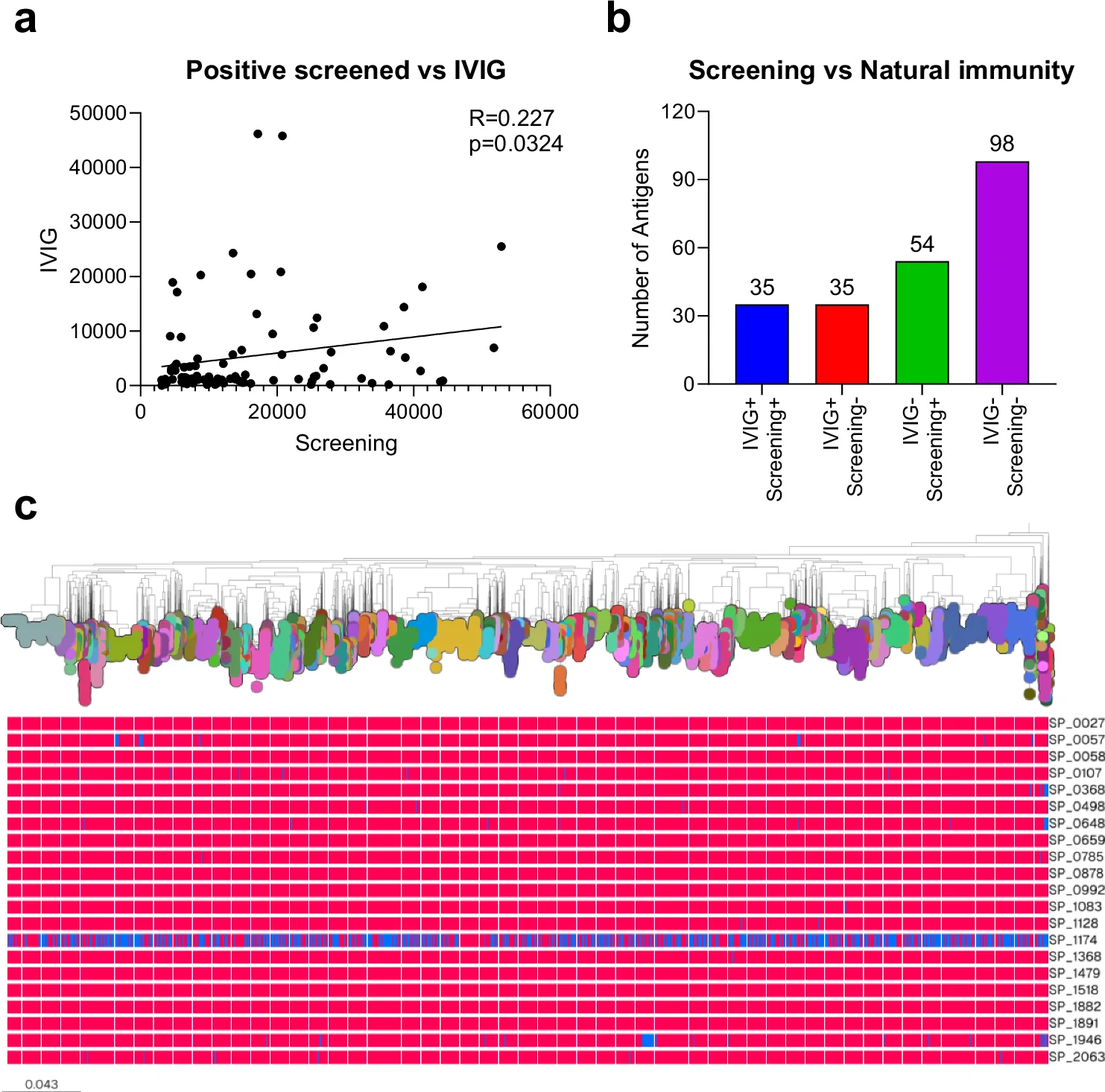
Vaccine antigens conservation across *S. pneumoniae* strains. **a** We report the correlation between the response measured 89 positive antigens at the high-dose screening in the vaccinated mice sera versus the array response to pooled human IgG (natural colonisation). Each dot represents an antigen; Correlation analyses were performed using Spearman’s rank correlation (two-tailed) to calculate the correlation coefficient (*R*) and *p* values that are reported in the top left corner. **b** The number of antigens positive or negative for both datasets is reported, as well as the number of antigens that were positive only in one of the datasets. **c** Heatmap to show the presence and absence of genes encoding the 22 candidate antigens produced as recombinant proteins in a global collection of 20,938 pneumococcal genomes. Presence (red) is defined by ≥80% identity and ≥80% coverage to the reference genes in *S. pneumoniae* TIGR4 (accession number AE005672). The phylogenetic tree on top indicates the pneumococcal population structure coloured by Global Pneumococcal Sequence Clusters (GPSCs). This figure can be viewed interactively at https://microreact.org/project/pneumococcal-protein-antigens.

Probing the microarray with IVIG identified, at a population level, naturally acquired human serum IgG responses (Supplementary Data 1—array validation samples) and allowed the direct comparison of protein antigenicity between humans and mice. The MFIs for individual proteins in IVIG showed a weak correlation probing to the MFIs for the high dose mouse screening positive antigens, with a significant proportion of proteins showing positive IgG responses in both IVIG and the screen (Fig. 3a). Comparing the positive and negative proteins after probing the microarray with IVIG and high dose screening mouse sera showed that most antigens behaved consistently across both groups, with 35 (15.8%) positive and 98 (44.1%) negative in both IVIG and vaccinated mice (Supplementary Data 4). Of the remaining proteins, 54 (24.3%) were positive in the screen but negative in IVIG, and 35 (15.8%) antigens were negative in the screen but positive in IVIG (Fig. 3b). This result shows that a good proportion of the beads-linked proteins (59.9%) are capable of inducing a response comparable to natural colonisation, suggesting a similar level of exposure, accessibility to the immune system and native conformation for these antigens. Other antigens that normally fail to trigger a response after colonisation (24.3%) were able to elicit a strong response when delivered with the bead-linked formulation. This suggests that such proteins, which initially generate no response, could be promising vaccine targets, as starting from a negative baseline may offer greater potential to enhance protection.

### Antigen selection for further vaccine characterisation

To assess the vaccine potential of proteins identified by the mouse antigen screen, 24 were selected for expression as recombinant proteins for further vaccination studies, of which 22 were successfully expressed (Supplementary Data 1—screening steps, Supplementary Data 4—candidates features). Proteins previously extensively used for vaccine studies (e.g. PspA, PspC, Ply, PsaA) were excluded. Proteins were selected to capture those that elicited positive IgG responses in (i) both IVIG and the mouse screen (10 proteins), (ii) only in the mouse screen (7 proteins), (iii) only in IVIG (4 proteins), plus as a negative control one protein that was negative in IVIG and the mouse screen (Table 1, Supplementary Data 4—candidates features). For selected proteins that were positive in the mouse screen, eight exhibited high microarray IgG MFIs (>30,000), while the remaining proteins were chosen to represent a range of lower MFIs. Selected proteins positive in IVIG were enriched for those with relatively low MFI, as these are more likely to represent proteins to which circulating IgG can be readily increased compared to proteins with high MFIs when the microarray was probed with IVIG.

**Table 1:** *S. pneumoniae* proteins shortlisted for further evaluation

TIGR4 number	Name	Function	Size	Localisation	Mouse antigen screen		IVIG	
			KDa		Positive	MFI	Positive	MFI
SP_0027	RPPK	Ribose-phosphate diphosphokinase; PRPP synthesis	35.4	Cytosol	No	266.7	No	238.2
SP_0057b	StrH	N-acetyl-glucosaminidase	79.5	Cell wall/secreted	Yes	17,011.1	Yes	15,309.2
SP_0058	GntR family regulator	HTH transcriptional regulator	27.9	Cytosol	Yes	36,358.9	No	231.2
SP_0107	Sep	LysM peptidoglycan-binding protein	17.9	Surface/cell-wall	Yes	8810.4	Yes	19,712.2
SP_0368c	Eng	Endo-α-N-acetylgalactosaminidase	76	Cell wall-anchored surface protein	Yes	25,860.2	Yes	14,585.2
SP_0498b	Endo-beta-N-acetylglucosaminidase	Putative exported lipoprotein	92.3	Cell wall-anchored surface protein	No	1501.6	Yes	13,736.2
SP_0648a	BgaA	Beta-galactosidase	84.8	Cell wall-anchored surface protein	No	1383.6	Yes	7995.2
SP_0648c	BgaA	Beta-galactosidase	99.8	Cell wall-anchored surface protein	Yes	38,572.8	Yes	12,456.2
SP_0659	Etrx1	Thioredoxin family protein	19.2	Cytosol	Yes	26,824.9	Yes	4558.2
SP_0785	-	Membrane fusion protein	41.8	Secreted/surface exposed.	Yes	20,549.9	Yes	20,277.2
SP_0878	FtsK/SpoIIIE	Cell division	66.3	Cytosol	Yes	44,285.7	No	483.2
SP_0992	RocS	DNA-binding protein	19.8	Transmembrane protein	Yes	24,981.7	No	599.2
SP_1083	-	Putative transcriptional regulator	48.5	Cytosol	Yes	19,470.1	No	829.2
SP_1128	Eno	Enolase	47	Cytosol	Yes	7469.5	No	1066.2
SP_1174	PhtB	Manganese and zinc uptake	90.3	Secreted/surface-associated	Yes	4703.7	Yes	15,259.2
SP_1368	Psr	Regulatory protein	35	Cytosolic	Yes	51,743.7	Yes	6890.2
SP_1479	PgdA	Peptidoglycan N-acetylglucosamine deacetylase A	48.6	Membrane-associated	Yes	35,654.0	Yes	13,253.2
SP_1518	MltG	Endolytic murein transglycosylase	37.9	Transmembrane protein	Yes	41,287.0	Yes	16,493.2
SP_1882	-	Conserved hypothetical	18.3	Membrane-bound protein	Yes	16,152.3	No	416.2
SP_1891	AmiA	ABC transporter	70.3	Lipoprotein/surface	No	0	Yes	7556.2
SP_1946	PlcR	Putative transcriptional regulator	34.9	Cytosolic	Yes	33,931.4	No	548.2
SP_2063	LysM domain protein	Peptidoglycan-binding protein	38.1	Surface/cell-wall associated	No	0	Yes	16,601.2

Whether selected proteins were conserved across *S. pneumoniae* strains was analysed using the Global Pneumococcal Sequencing (GPS) database, which contains 20938 pneumococcal genomes at the time of writing^33,34^. For the majority of the candidates, there was a high degree of conservation with 97–100% present in the global collection (Fig. 3c). The exception was SP_1174, which was present in only 49.5% (10361/20938) of isolates. To assess whether any common pneumococcal lineages (GPSCs with *n* > 100) exhibited a high frequency of candidate protein absence, we found that 94.5% of GPSC42 isolates were absent for SP_1946, 68.8% of GPSC61 and 15.3% of GPSC8 isolates were absent for SP_0057, and 10.6% of GPSC106 isolates were absent for SP_2063. All other candidates were present in >90% of genomes in each GPSC.

### Mouse vaccination with selected antigens

**Fig. 4:**
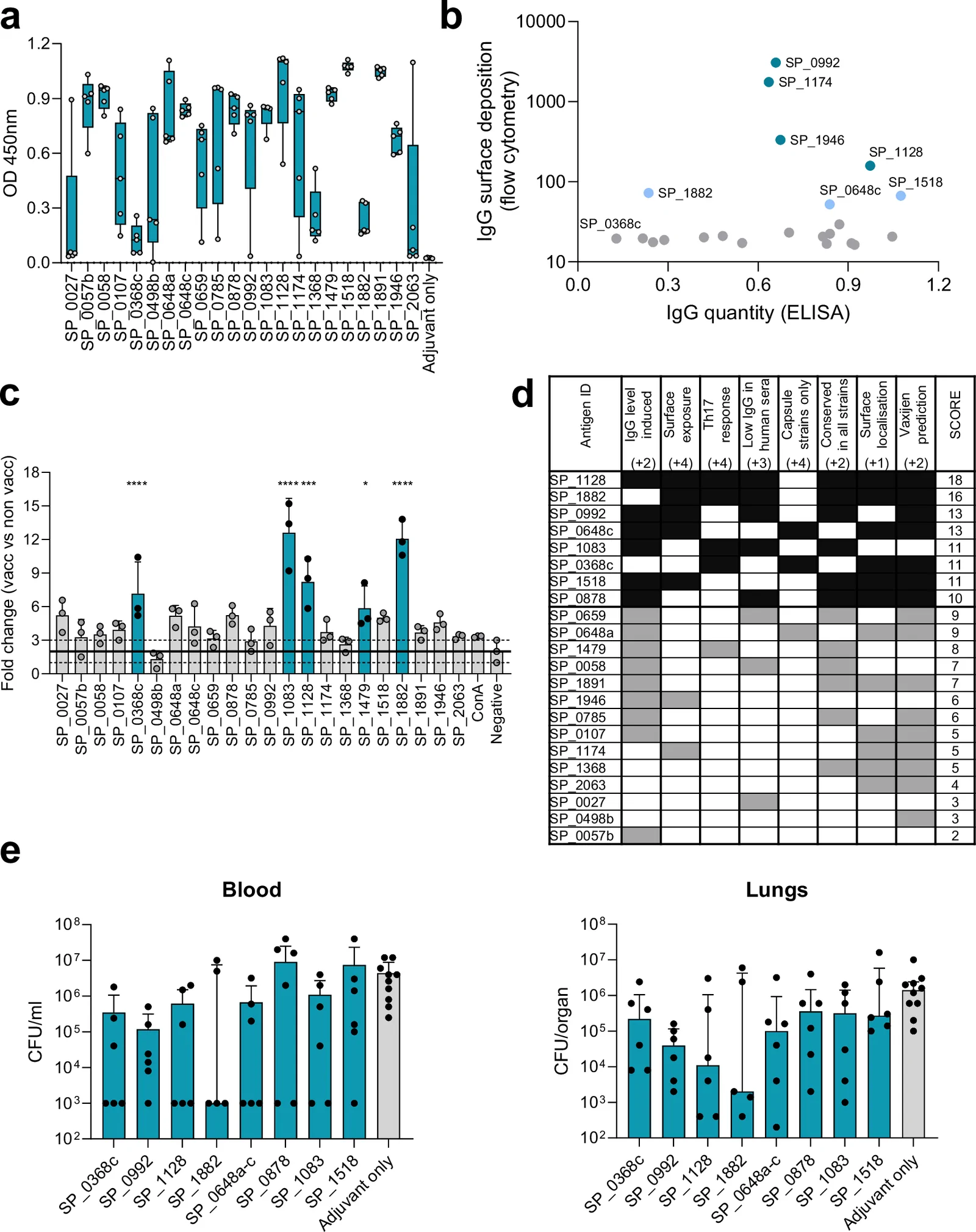
Single protein immune response. **a** Protein ELISAs were performed using sera from mice immunised three times by intraperitoneal injection with 10 µg of each of the 22 selected recombinant proteins. IgG levels for each individual antigen are shown (floating bars show line at median). PBS vaccinated mice sera were included as a negative control (5 mice/group, total number of mice *n* = 115). **b** Protein ELISA results were correlated to IgG opsonisation on *S. pneumoniae* measured by flow cytometry using the average values for IgG deposition on three separate strains (TIGR4, 6B and BVJ1JL) for each antigen. Each dot represents an antigen, and the colour indicates the degree of IgG opsonisation: high (teal), intermediate (light blue) and low levels (grey). **c** IL-17 release after 24 h stimulation of splenocytes obtained from mice vaccinated with the 22 shortlisted antigens (11 antigens/group and adjuvant only cohort, 5 mice/group, total number of mice *n* = 15) measured using Elispots reported as fold-changes between the number of spots measured in vaccinated and unvaccinated splenocytes. Concavallin A was used as a positive control, and DMSO as a negative. Bars indicate the average ratios, error bars the standard deviations. Red continuous and dotted lines indicate the average baseline signal (DMSO) and the threshold of variability from baseline. The Kruskal–Wallis test was used to identify statistically significant signals (*p < 0.05, ***p < 0.001, and ****p < 0.0001). Significant IL-17 release has been measured in vaccinated mice splenocytes exposed to SP_1128 (*p* < 0.0001), SP_1882 (*p* < 0.0001), SP_1479 (*p* = 0.0249), SP_0368c (*p* = 0.0009), and SP_1083 (*p* < 0.0001). **d** A table summarising the protein features (with weighted scores) was used for the selection of the antigens to be used in a mouse model of protection (coloured boxes indicate the presence of that characteristic, the boxes are coloured in black for the 8 shortlisted antigens and in grey for the others). **e** Colony-forming units (CFU) evaluated in the blood and lungs of mice vaccinated three times by intraperitoneal injection with 10 µg of 8 individual protein antigens (SP_0992, SP_1083, SP_1128, SP_0648a, SP_0878, SP_1882, SP_1518 and SP_0368c), challenged using an intranasal challenge dose of 1 × 10⁷ CFU TIGR4 (representing a pneumonia model) 24 days after the last vaccination, then culled after 24 h. Dots represent individual mice in each group (6 mice/group, total number of mice *n* = 54), bars represent the average CFU values, and error bars represent the standard deviations.

To assess the humoral and cellular responses to the 22 recombinant proteins, groups of CD1 mice were vaccinated with each protein (*n* = 5 per protein). Serum IgG responses were measured by protein ELISAs, and IgG opsonisation of live *S. pneumoniae* was assessed using flow cytometry for *S. pneumoniae* strains TIGR4 (serotype 4), BHN418 (serotype 6B), and BVJ1JL (serotype 1) (Figs. 4a, b and S6 and Supplementary Data 4). The ELISA data showed major variations in results for individual proteins. SP_0027 (the control protein with negative IgG responses in both IVIG and mouse screen) showed poor ELISA responses, whereas 6 of 8 proteins with high MFIs (>30,000) in the mouse screen also had high protein ELISA titres (SP_0058, SP_0648c, SP_0878, SP_1479, SP_1518, and SP_1946) (Figs.4a and S5B). The IgG opsonisation results varied with vaccine antigen and target strain (Supplementary Data 4); for example, it is interesting to observe how the anti-SP_1174 sera show specific binding only against the TIGR4 strain, which is the only strain tested in which the antigen should be present. We reported the data as a correlation matrix of the average IgG MFI to the three strains versus the results for the protein ELISA IgG to identify proteins that induced strong IgG responses capable of opsonising different *S. pneumoniae* strains (Fig. 4b). To assess differences between proteins in their ability to induce cellular immunity, mice were vaccinated (three intraperitoneal doses containing 10 µg of recombinant protein plus aluminium hydroxide adjuvant) with pools of 10 to 12 antigens pools, splenocytes were isolated 3 weeks after the last vaccination, re-stimulated with individual recombinant proteins, and IL-17 release was measured using Elispots (Fig. 4c). Four proteins (SP_0368c, SP_1083, SP_1479, SP_1882) induced a significant IL-17 response compared to unvaccinated controls, two of which induced only poor IgG responses when measured by whole cell ELISAs and flow cytometry assays of IgG opsonisation (SP_0368c and SP_1882).

### Vaccination with selected antigenic proteins protects mice against *S. pneumoniae* pneumonia and sepsis

The above data were used to generate a weighted score for each immune and antigen characteristic which was used to select SP_0368c, SP_0648c, SP_0878, SP_0992, SP_1083, SP_1128, SP_1518 and SP_1882 for testing of their protective efficacy when used as single antigen vaccines in CD1 mice (*n* = 6/group) mice (Fig. 4d). Mice were then challenged by intranasal inoculation with *S. pneumoniae* TIGR4, and colony-forming units (CFUs) obtained by plating blood and lungs collected 48 h after challenge. Target organ CFU were reduced compared to the adjuvant-only control for most of the antigens tested, although as expected given the n number and number of vaccine groups these differences were not statistically significant (Fig. 4e). Based on the CFU data, we identified SP_0368c, SP_1882 (both of which induce strong Th17 responses but poor IgG opsonisation), SP_0992, and SP_1128 (both of which induce strong IgG opsonisation but poor Th17 responses), as the four most promising protective protein antigen candidates to be taken forward for testing as a multivalent vaccine.

### Epitope mapping of SP_0368c, SP_1882, SP_0992, and SP_1128

These candidates have not been considered before for a vaccine approach, yet in our mouse model, they showed protective potential against pneumococcal challenge. SP_1128 encodes alpha-enolase (Eno), a highly conserved glycolytic metalloenzyme that also translocates to the pneumococcal surface independently of classical secretion signals, where it functions as a plasminogen receptor. Pneumococcal Eno binds human plasmin(ogen) with high affinity via a nine-residue internal motif (residues 248–256) that is fully exposed on the surface of its octameric assembly, enabling the bacterium to acquire host proteolytic activity and thereby promote tissue invasion and immune evasion^35–37^. SP_0368 is annotated as an endo-α-N-acetylgalactosaminidase (Eng), and was previously shown to be involved in cleaving *O*-linked glycans from host glycoproteins^38^, a function that could facilitate persistence on mucosal surfaces and make the protein accessible to immune recognition. Indeed, previous studies showed that the Eng mutant has a reduction in adherence to human airway epithelial cells and a significantly decreased ability to colonise the upper respiratory tract^38^. In contrast, SP_0992 and SP_1882 are described only as conserved hypothetical proteins with no clear functional assignment. Their localisation is likewise uncertain, but their conservation across pneumococcal isolates and the protection observed in our model suggest they warrant further investigation as novel vaccine antigens.

**Fig. 5:**
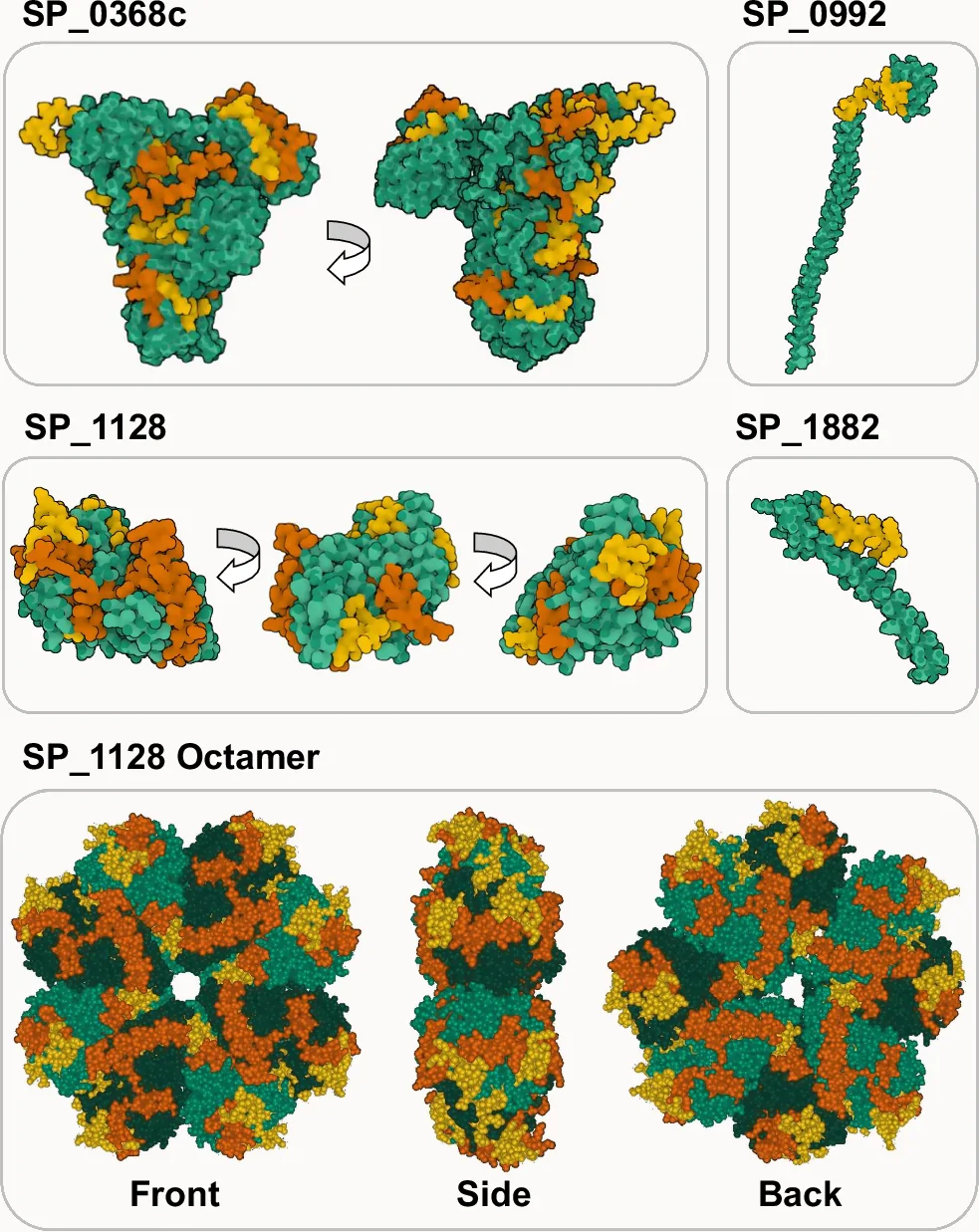
Epitope mapping of the four candidates included in the vaccine formulation. Protein structural prediction has been calculated using AlphaFold2 methodology (green backbone) for the 4 antigens included in the vaccine combination. Epitope array data guided us to highlight the regions in each antigen bound by human IVIG. Regions recognised strongly by IVIG are highlighted in yellow, while regions with intermediate signal are shown in orange. For SP_1128 (enolase), both its single monomer and the octameric form are shown. Full sequences are reported in the supplementary Data 5 file.

The epitopes recognised by human IgG in IVIG were identified by probing a 15 amino acid epitope array at one amino acid resolution covering the full sequences of SP_0368c, SP_1882, SP_0992, and SP_1128 with IVIG. This identified multiple strongly recognised epitopes for SP_0368c (nine in total) and SP_01128 (five in total), and one each for SP_0992 and SP_1882 (Supplementary Data 5). Mapping the linear epitopes with the highest IgG binding in human IVIG to the AlphaFold2 structural prediction for each protein (Fig. 5) identified epitopes that are likely to be surface exposed for all four vaccine candidates. Interestingly, for antigen SP_1128 (alpha-enolase), human IgG recognition is restricted to epitopes exposed on the surface of the assembled octamer; the inter-monomer contact interfaces, which are buried upon oligomerisation, are not recognised by IVIG. This is consistent with the crystal structure of pneumococcal alpha-enolase, which demonstrated that residues at the dimer-dimer interfaces are inaccessible to solvent and ligand binding, and supports the conclusion that naturally acquired antibodies target the surface-exposed regions of the functional oligomeric form of the protein.

### Vaccination of mice with a combination of SP_0368c, SP_1882, SP_0992, and SP_1128 protects against sepsis and pneumonia

**Fig. 6:**
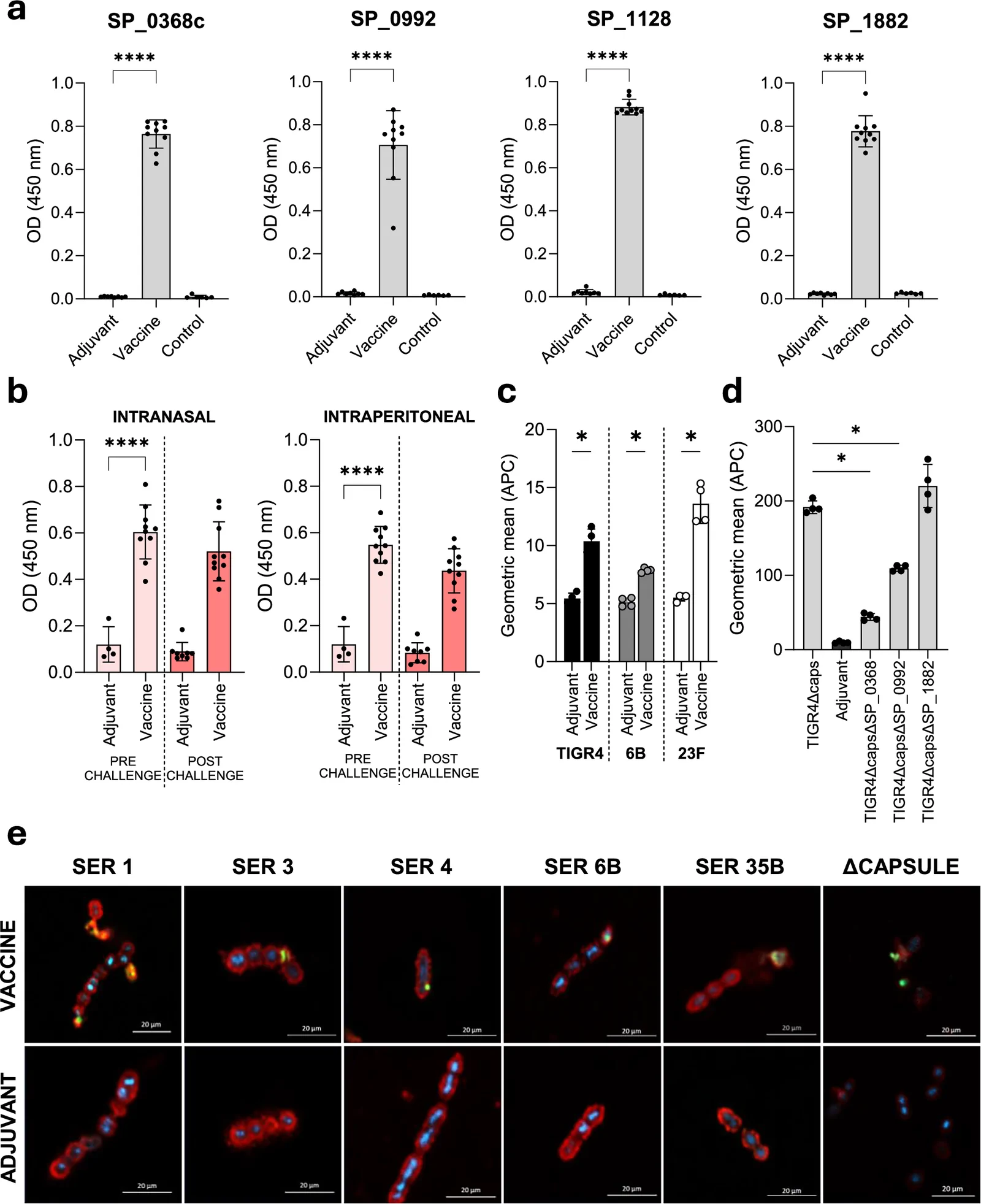
Antibody response generated by a combination vaccine of the selected antigens. **a** Specific IgG responses for each individual antigen included in the combination vaccine have been measured by protein ELISA using mouse sera post vaccination (8–10 mice/group, total number of mice *n* = 36). Negative controls were also added, including sera from mice immunised with adjuvant alone (Alhydrogel) and PBS-only coated wells (control). Data are presented as individual data points. Bars represent the mean, and error bars indicate the standard deviation (SD). A Kruskal–Wallis test with Dunn correction (two-tailed) was used to identify statistically significant differences (****p < 0.0001). **b** The specificity for *S. pneumoniae* of the antibodies generated by vaccination has been tested by Whole-cell ELISA against the TIGR4 strain. Sera obtained before (light red) and post challenge (red), were compared to the adjuvant-only immunised mice (10 mice/group for the vaccine and 8 mice/group for the control, *n* = 18). Data are presented as individual data points. Bars represent the mean, and error bars indicate the standard deviation (SD). A Kruskal–Wallis test was used to identify statistically significant differences (****p < 0.0001). Serum deposition using sera from mice vaccinated with the quadrivalent vaccine and adjuvant alone was tested to measure IgG binding on WT capsulated bacteria (**c**) and unencapsulated mutants (**d**). Pooled sera from the vaccine (*n* = 10) and control (*n* = 8) groups have been tested in three independent experiments. Single mutants from three out of the four antigens included in the vaccine have been assessed. Data are presented as individual data points. Bars represent the mean, and error bars indicate the standard deviation (SD). To test statistical significance, a Mann–Whitney *U* test (two-tailed) has been used in (**c**) (*p < 0.05, *p* = 0.0209) and a Kruskal–Wallis test with Dunn’s correction was used in (**d**) (*p < 0.05, *p* = 0.0286). **e** The capability of antibodies induced by vaccination to bind different strains of pneumococcus was tested by confocal imaging. Stained with anti-capsule antibody (red), sera from vaccinated mice (green) and DAPI (blue). Adjuvant-only (Alhydrogel) immunised mice serum was used as a negative control. The images presented are representative of results obtained from three independent experimental replicates.

The protective efficacy of vaccination with SP_0368c, SP_1882, SP_0992, and SP_1128 in combination was assessed in mice using three intraperitoneal inoculations of the combination of the four antigens with the addition of Alhydrogel adjuvant. ELISAs demonstrated that sera from vaccinated mice had strong protein IgG responses to each individual protein (Fig. 6a) and to the *S. pneumoniae* TIGR4 strain (Fig. 6b). Repeating whole cell ELISAs with sera obtained after *S. pneumoniae* infection challenge demonstrated a slight drop in OD compared to pre-infection sera, perhaps representing binding of circulating antibodies to infecting *S. pneumoniae*. Flow cytometry was used to assess the functionality of antibodies induced by measuring the capability to opsonise three different *S. pneumoniae* encapsulated strains (Figs. 6c and S7). To identify the dominant antigens contributing to opsonisation with IgG, these assays were repeated for unencapsulated mutant strains for three of the four antigens (TIGR4Δ*cps*Δ*SP_*0368, TIGR4Δ*cps*ΔSP_0992, and TIGR4Δ*cps*ΔSP_1882). We were unable to obtain a ΔSP_1128 strain, possibly because this is an essential gene^39,40^. The results showed a significant reduction in IgG binding in the TIGR4Δ*cpsΔSP_*0368 and TIGR4Δ*cpsΔ*SP_0992 strains (Fig. 6d). Loss of *sp_1882* gene did not significantly affect IgG opsonisation in serum from mice vaccinated with all four antigens, but this protein was selected for inclusion in the multivalent vaccine largely due to its strong induction of Th17 responses (Fig. 4c). Fluorescent microscopy also confirmed the capability of antibodies induced by the quadrivalent protein vaccine to bind six *S. pneumoniae* strains, representing recognition of multiple capsular serotypes and strain backgrounds (Fig. 6e).

**Fig. 7:**
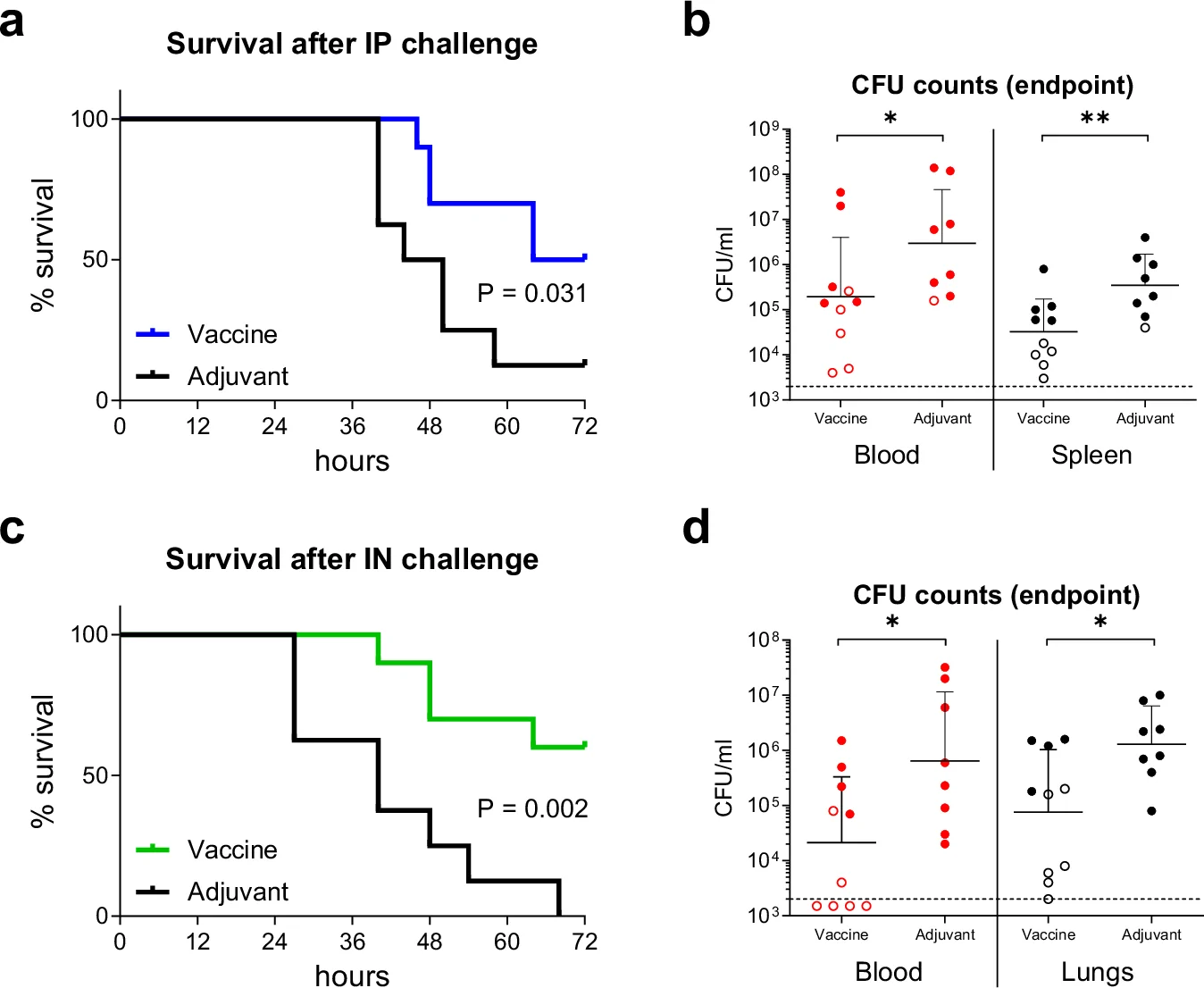
Protective effect of selected antigen combination vaccine in mouse models. Groups of CD1 mice (*n* = 10, vaccinated) were vaccinated with a combination of 4 pneumococcal antigens: SP_0992, SP_1128, SP_1882 and SP_0368c using 10 µg/dose of each antigen plus Alum adjuvant (Alhydrogel) on 3 occasions 2 weeks apart. An adjuvant-only group was included as a negative control (*n* = 8, adjuvant). All groups were infected 4 weeks after the third dose of vaccine with a lethal dose of *S. pneumoniae* strain. Vaccine protective efficacy was tested in a mouse model of pneumococcal sepsis using intraperitoneal inoculation of 4 × 10^4^ CFU of TIGR4 to generate **a** survival curves (mice culled when showing severe symptoms of sepsis) and **b** blood and spleen CFU measured at the endpoint (on culling or at 72 h). Data are presented as individual data points. The horizontal centre lines represent the median, and the whiskers indicate the interquartile range (bounds of the box corresponding to the 25th and 75th percentiles). Open circles represent values below the lower limit of quantification (LLOQ), plotted at the detection threshold. Vaccine protective efficacy was tested in a mouse model of pneumococcal pneumonia using intranasal inoculation of 2 × 10^7^ CFU of the TIGR4 strain to generate **c** survival curves (mice culled when showing severe symptoms of infections) and **d** blood and lung CFU measured at the endpoint (on culling or at 72 h). Data are presented as individual data points. The horizontal centre lines represent the median, and the whiskers indicate the interquartile range (bounds of the box corresponding to the 25th and 75th percentiles). Open circles represent values below the lower limit of quantification (LLOQ), plotted at the detection threshold. Survival curves were analysed using the log-rank Mantel–Cox test and target organ CFU using two-tailed Mann–Whitney *U* tests (*p < 0.05).

When challenged with *S. pneumoniae* TIGR4 using the pneumonia (intranasal inoculation) and a sepsis (intraperitoneal inoculation) model, mice vaccinated with the quadrivalent vaccine showed improved survival compared to the adjuvant control in both models (Fig. 7). Culturing target organs (blood and spleen for the sepsis model, blood and lungs for the pneumonia model) showed significantly reduced CFU levels in the vaccinated groups. Overall, these data show that a quadrivalent vaccine using protein antigens identified by our screening approach and combining induction of Th17 and humoral responses can protect against *S. pneumoniae* pneumonia and septicaemia.

## Discussion

Building on the foundation of reverse vaccinology, our work introduces FGV as an alternative conceptual and methodological framework for bacterial vaccine discovery. FGV integrates genome-guided antigen selection with proteomic screening and in vivo functional validation, thereby transforming antigen identification from a predictive exercise into an experimentally driven process. By directly mapping the relationship between genomic conservation, immune recognition, and protection, FGV provides a systematic route to identify conserved vaccine targets that elicit both humoral and cellular immunity.

FGV was applied to *S. pneumoniae* to assess the relative antigenicity of 222 protein antigens through pooled bead-based vaccination in mice, followed by quantitative measurement of serum IgG responses using a protein microarray. The screen identified 91 proteins capable of eliciting IgG responses, most of which were strongly conserved across a global collection of >20,000 pneumococcal genomes. These proteins included many that have previously been identified as vaccine candidates, including PspA (SP_0017), PspC (SP_2190), PsaA (SP_1650), StkP (SP_1732), PiuA (SP_1872); the notable exceptions were Ply (SP_1923) and PiaA (SP_1032). Further vaccine experiments with single proteins identified four proteins, which, when used in combination, showed protective efficacy in murine models of pneumonia and sepsis. Our findings have several important implications: they substantially expand the potential number of known vaccine candidates for *S. pneumoniae*, provide some insight into the balance between humoral and Th17-mediated immunity, and demonstrate the feasibility of a scalable antigen discovery strategy that can be applied broadly to other pathogens. Irrespective of being a *S. pneumoniae* protein-based vaccine, our selection of these four antigens would be valuable as carrier proteins for glycoconjugate vaccines, particularly bioconjugate vaccines^41^.

**Fig. 8:**
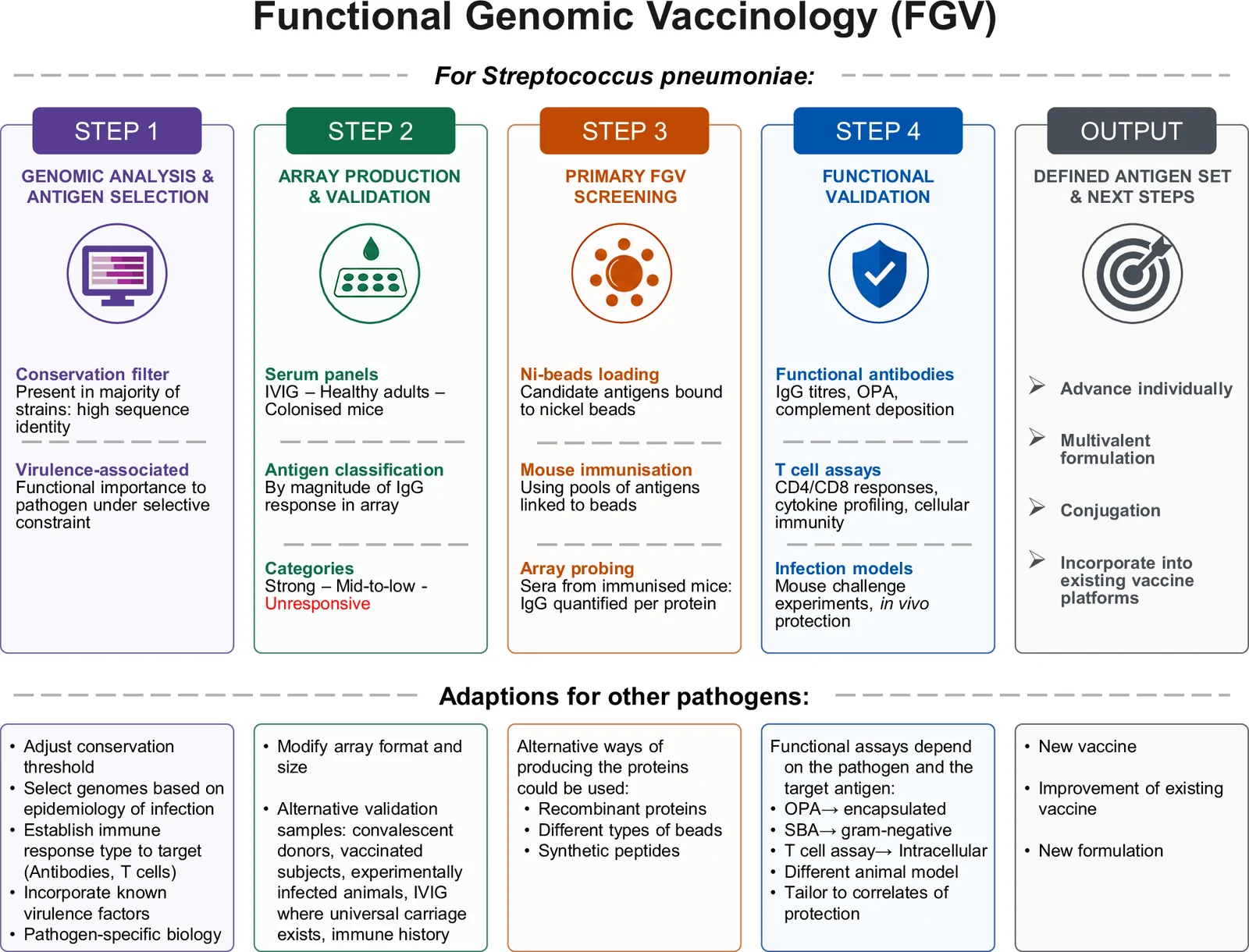
Functional Genomic Vaccinology (FGV) pipeline. Four-step workflow from pan-proteome analysis to functional antigen validation. Adaptations for other bacterial pathogens are listed in the bottom panel.

A major obstacle to protein-based vaccines for bacterial pathogens has been the difficulty of selecting antigens with both broad strain coverage and strong immunogenicity. Previous efforts relied largely on candidate-driven approaches^11,15,42–44^ or using human serological responses to identify antigenic proteins^7^. While these approaches often identify antigens that are immunogenic and induce protective immunity in animal models, human sera often contain high IgG levels to the candidate antigens^14,23,45^, which limits the ability to boost responses by vaccination and, in some cases, biases vaccination towards boosting non-protective IgG^46^. In addition, many candidates (e.g*. S. pneumoniae* PspA and PspC) show marked allelic variation between strains, making them less effective vaccine candidates^28,29^. FGV offers several major advantages. Firstly, it is an unbiased strategy with no need to predict in advance which proteins are likely to be good antigens. Secondly, the screen rapidly provides data on dozens of proteins despite using relatively limited numbers of mice; once the nickel beads and microarray were available, screening 222 proteins was completed within 3 months using 115 mice (for each of the doses tested). Multivalent *S. pneumoniae* vaccines now use 25 different capsular antigens, suggesting the efficiency of our screening technique could be improved further by using nickel beads carrying at least 25 different proteins. Although many of the antigens identified by the screen also showed significant IgG responses in human sera, there was limited IgG binding to 54 proteins in IVIG studies. This highlights another advantage of the FGV approach: its ability to identify bacterial antigens that, although weakly immunogenic during natural infection—possibly due to low or transient expression—elicit strong IgG responses when delivered as purified proteins, thereby expanding the vaccine candidate repertoire beyond the conventional targets. The main disadvantage of our screen is its dependency on a protein microarray to analyse antibody responses, the expense of which can limit the number of proteins investigated. For this reason, our screen remains incomplete, with another 1138 conserved *S. pneumoniae* proteins yet to be assessed for their antigenicity. We developed the screen using *S. pneumoniae* due to its relatively small genome and the extensive existing data on genome variation, human IgG responses, and vaccine candidates. However, the FGV approach is pathogen-agnostic and could be applied to other high-priority bacterial pathogens for which there are no existing vaccines, including *Klebsiella pneumoniae*, *Escherichia coli*, *Acinetobacter baumannii* or *Staphylococcus aureus*^47–50^. To facilitate application of FGV to other bacterial pathogens, we outline the four-step process and how each stage can be adapted (Fig. 8). In Step 1, proteins conserved across the majority of strains are prioritised, supplemented where appropriate with virulence-associated targets. In Step 2, the microarray is validated using well-characterised serum panels—sourced from naturally exposed adults, convalescent donors, vaccinated subjects, or experimentally infected animals, depending on the pathogen—to classify antigens by natural immunogenicity. In Step 3, nickel beads loaded with candidate antigens are used to immunise mice, and resulting sera are probed on the array to quantify specific IgG responses. In Step 4, top candidates are produced as recombinant proteins and assessed for functional antibodies, T cell responses, and in vivo protection using assays tailored to the relevant correlates of protection. The outcome is a defined antigen set that can be advanced individually, combined into a multivalent formulation, or incorporated into existing vaccine platforms, providing a generalisable framework for pathogens where antigenic diversity or limited immunological understanding has hindered vaccine development.

For pneumococcus, we used various criteria to identify a subset of proteins for further investigation as vaccine candidates, including limited previous data of their utility as vaccines, relative antigenicity in the mouse screen, conservation amongst *S. pneumoniae* strains, and the level of IgG responses in pooled human IgG (selecting those without strong responses to allow boosting by vaccination). Multiple criteria were used as the strength of antigenicity alone may result in selecting proteins that induce high levels of IgG that are poorly protective due to a lack of target antigen on the bacterial surface or poor antibody efficacy at promoting phagocytosis, or for which existing strong IgG responses in humans limit the potential for boosting by vaccination. The down-selection strategy balanced technical feasibility with biological diversity, ensuring inclusion of proteins recognised across different contexts, and led to testing 22 proteins for their immunogenicity individually in mice. This generated a rich comparative dataset linking antibody titres to IgG opsonisation, along with splenocyte Th17 responses measured, testing 10 antigens in each group of mice. Notably, the data showed that high levels of antigen recognition by ELISA did not directly translate to high levels of IgG opsonisation of live bacteria or strong Th17 cellular responses. Emerging evidence emphasises the importance of IL-17A-producing Th17 cells in protection against *S. pneumoniae* colonisation and pneumonia^51^, suggesting that optimal protection may require a combination of antigens that stimulate both humoral and cellular immunity^52^. However, the precise contribution of Th17 responses to protection in humans, particularly relative to antibody-mediated immunity, is still being defined. This rationale guided the design of our quadrivalent formulation, which combines antigens with complementary immunological profiles: SP_0368c and SP_1882, which induced strong Th17 responses despite generating relatively low IgG titres, and SP_0992 and SP_1128, which elicited IgG capable of opsonising S. pneumoniae but induced limited IL-17A responses in splenocytes. This combinatorial approach echoes previous findings with protein-polysaccharide conjugates, where immune synergy enhances efficacy^4,53^. The quadrivalent vaccine (SP_0368c, SP_1882, SP_0992, SP_1128) protected mice in both pneumonia and sepsis models, reducing bacterial burden and improving survival. Importantly, antibodies elicited by this formulation recognised multiple pneumococcal serotypes, supporting the potential for serotype-independent immunity^54^. The available functional data on our antigens is limited. SP_0368 encodes a likely cell surface exo-glycosidase involved in carbon acquisition from host glycans during colonisation, but there are very limited functional data on its role during infection^38^. SP_1128 encodes an enolase that forms octamers, which our epitope mapping suggests could result in the repetitive expression of antigenic epitopes on the *S. pneumoniae* cell surface^37,55^, a property that would be predicted to improve antibody efficacy in providing protective immunity. Little is known about SP_1882 and SP_0992; both these proteins are predicted to have linear structures, which the epitope mapping suggests have terminal antigenic components*.* Previously studied pneumococcal vaccine candidates were intentionally excluded from downstream testing to allow an unbiased evaluation of additional antigens and to demonstrate the capacity of FGV to expand antigen discovery beyond traditional candidates. Further development of our vaccine candidates is likely to include testing with previously characterised candidates to identify the most effective antigen combinations for protective efficacy, direct comparative efficacy of vaccination with antigen combinations to single antigens, and protection against nasopharyngeal colonisation. Epitope mapping using human IgG highlighted the linear epitopes likely to induce IgG responses to each antigen, data that can be used to potentially design a multi-epitope fusion protein^12,56^. However, antigen conformation is likely to be key for the induction of effective immune responses, and integration of structure-based analyses (e.g. using AlphaFold-derived structural conservation) is likely to be an important direction for further refinement of the FGV pipeline.

The major strength of this work is the integration of multiple complementary approaches—protein microarrays, bead-based high-throughput vaccination, recombinant protein immunisation, and functional readouts including IgG binding, opsonisation, and Th17 ELISpot. This multi-layered design provides a robust framework for antigen prioritisation. However, several limitations should be acknowledged. First, murine models, while invaluable, may not fully recapitulate human immune responses, and translation of candidate efficacy to humans remains uncertain^57,58^. Second, the relatively small group sizes limited statistical power, particularly in protection experiments with individual antigens. Third, adjuvant choice can strongly influence Th17 versus antibody skewing^59^, and while Alum was suitable for these proof-of-concept studies, alternative adjuvants may be required for optimal performance in future formulations.

In summary, we report the development of a scalable antigen discovery pipeline applied here to *Streptococcus pneumoniae*. FGV revealed a spectrum of conserved immunogenic proteins and facilitated the design of a multivalent formulation conferring serotype-independent protection. Beyond pneumococcus, the same approach can be adapted to other bacterial pathogens, representing a scalable and generalisable paradigm for next-generation bacterial vaccinology, capable of accelerating the translation of genomic data into clinically relevant vaccines.

## Methods

### Ethics statement

This study complies with the relevant ethical regulations.

#### Human research

Serum samples were obtained from six healthy adult volunteers (aged 24–38 years; mean age 29.5 years) under the UCL Research Ethics Committee protocol number: 3078/001 and conducted in accordance with institutional and national ethical guidelines. The cohort included 3 males and 3 females, determined via participant self-report. Sex was considered in the initial study design by ensuring an equal balance of male and female volunteers. However, data were not disaggregated by sex during downstream protein array analysis due to the small sample size (*n* = 3 per sex), which lacked sufficient statistical power to perform stratified comparative analyses. All participants provided written informed consent prior to sample collection. No vulnerable populations were included, and all samples were anonymised prior to analysis. Participants received no financial compensation for their participation.

#### Animal research

All mouse experiments were carried out in the Biological Services facility at University College London under a UK Home Office project license (PP3031297 Scientific Procedures Act 1986) approved by the local ethical committee and following institutional guidelines for the care and use of laboratory animals. Sample sizes were determined a priori to provide sufficient statistical power for meaningful results while avoiding unnecessary use of animals. Mice were group-housed in individually ventilated cages under specific-pathogen-free conditions at the UCL Biological Services Animal Unit. The holding room was maintained under a controlled 12-h light/dark cycle. Ambient temperature was regulated at 21 ± 2 °C with a relative humidity of 55 ± 10%, and animals had ad libitum access to standard rodent chow and water.

### Bacterial strains and methods

*S. pneumoniae* strains used in this study were TIGR4 (serotype 4), BVJ1JL (serotype 1), 0100993 (serotype 3), BHN418 (serotype 6B), 2896/13 (serotype 35B), JSB23F (serotype 23F) and TIGR4Δ*cps* (unencapsulated). Mutant strains containing complete replacement of SP_0368, SP_0992, and SP_1882 with spectinomycin antibiotic resistance cassette were made in the unencapsulated strain TIGR4Δ*cps* using synthetic DNA constructs and established transformation techniques^60^. *S. pneumoniae* were propagated either in Tryptic Soy Broth (TSB; Becton Dickinson, Wokingham, UK) or on Columbia agar plates (Becton Dickinson) enriched with 5% (v/v) defibrinated horse blood, under incubation conditions of 37 °C with 5% CO₂.

### Construction and probing of protein microarray

A protein microarray comprising 289 *S. pneumoniae* proteins was generated using an in vitro cell-free expression system, following previously established methods^31,45^. The antigens selected were highly conserved across more than 616 *S. pneumoniae* strains and included most of the proteins previously identified as IgG targets in sera from healthy adults (Supplementary Data 1). The array was constructed using genes amplified from the TIGR4 strain genomic DNA for all the genes except PspA and PspC. A phylogenetic tree in 349 clinical isolates^30^ guided the selection of four variants of PspA and PspC to be included in the array from strains D39 (serotype 2), TIGR4 (serotype 4), 10050 2#28 and 10071 4#59 (serotype 7F and 19F, respectively, DNA kindly gifted by Marien de Jonge and Jeroen Langereis)^30^. Genes were cloned into a T7 expression vector and proteins expressed by incubating the plasmids for 16 h in *E. coli*-based in vitro transcription/translation (IVTT) reactions (RTS *E. coli* HY 100 kit, biotech rabbit GmbH, Germany). Oligonucleotides for the cloning were purchased from Sigma Aldrich; their sequences are reported in Supplementary Data 1. Protein expression was tested by Western blotting using antibodies against N-terminal poly-histidine (His) after printing onto nitrocellulose-coated glass AVID slides (Grace Bio-Labs), using an Omni Grid 100 microarray printer (Genomic Solutions). Microarrays were probed as previously described^10,31^. Briefly, *s*erum samples diluted 1:50 in blocking buffer (Maine Manufacturing, Sanford, ME) and supplemented with *E. coli* lysate were applied to microarrays overnight. After a washing step, the staining was completed with a subsequent incubation with 1:200 diluted biotin labelled anti-mouse IgG (405303, lot B253219, Biolegend) and 1:250 diluted AF647 conjugated streptavidin (S21374, lot 2329429, ThermoFisher). Signal detection and data acquisition were carried out using the ArrayCAM® Imaging System (Grace Bio-Labs). Background-subtracted antibody responses were quantified and thresholds established for each protein for significant IgG responses using naïve mouse serum as a negative control^31,61^, defined as median fluorescence intensity exceeding two standard deviations above the negative control values and with a minimum MFI of 3000. Microarray data for *S. pneumoniae* single (*n* = 6)^31^ and double colonised mice (*n* = 6)^10^ were compared to results for healthy human sera (*n* = 6) and two purified pooled human immunoglobulin preparations (IVIG) (Flebogamma, Grifols and Vigam, bpl). This validation step reduced the array’s size to 253 antigens, discarding proteins giving consistently high background levels, uneven spots or weak signals compared to our reference array dataset^23^ (Supplementary Data 1—array validation samples).

### Protein expression and preparation of nickel bead vaccine antigens

222 *S. pneumoniae* proteins expressed using IVTT were captured directly onto Ni-NTA (His-tag Affinity) Magnetic Beads (0.5 μm; EmerTher, cat# AE04002). Briefly, beads were washed with PBST before coupling to His-tag proteins. Crude IVTT reactions (420 μl per protein), containing expressed His-tagged proteins, were incubated with the functionalised beads for 2 h at room temperature (or overnight at 4 °C) to allow antigen capture. Beads were then washed extensively (five times with ice-cold PBS containing 0.02% Triton X-114 and three times with PBST) to remove unbound proteins and residual endotoxin. Since *E. coli* proteins remain bound to beads despite multiple washes, a ‘No DNA’ control (IVTT reaction without plasmid, processed identically to test samples) was included in all assays to measure background reactivity. Successful protein capture was confirmed by silver staining and anti-His western blotting. Beads were stored in PBST at 4 °C and vortexed thoroughly before use in immunisation experiments.

### Genome analyses

The presence and absence of the genes encoding the protein candidates in the GPS database of 20,938 pneumococcal genomes was assessed using Abricate (version 0.8), creating a matrix of coverage and identity in percentages using ≥80% identity and ≥80% coverage to the reference *S. pneumoniae* TIGR4 genome (accession number AE005672). The output was visualised in a heatmap generated by the R package pheatmap (version 1.0.13). A phylogeny containing 20,938 pneumococcal genomes was built upon a single-nucleotide polymorphism (SNP) alignment generated from mapping genome reads to a reference *S. pneumoniae* genome (ATCC700669, accession number FM211187). The phylogeny was visualised on Microreact, along with the prevalence of genes of interest, GPSCs and serotype.

### Mouse models of vaccination and infection

Vaccination experiments used 5-week-old female CD1 mice (Charles River, UK) and established protocols^4,62^. Experimental procedures were not randomised or blinded. For the bead-conjugated protein vaccination model, mice (*n* = 5/group) received two intraperitoneal injections on days 1 and 10, with antigen pools at concentrations of 50, 100, or 400 ng with the addition of Squalene-Oil-in-water adjuvant (Addavax, InvivoGen, used as known to induce robust antibody responses to particulate antigens^63,64^), and sera were obtained on day 24. Intraperitoneal inoculations were used to facilitate consistent vaccination of large numbers of mice with the high vaccine volumes of 200 µl/injection required to standardise the antigen dose for pooled vaccination with multiple bead-linked antigens. For recombinant protein vaccinations, mice (*n* = 5/group) were vaccinated by intraperitoneal injection of 10 µg of protein plus aluminium hydroxide adjuvant (Alhydrogel, InvivoGen) on days 1, 14, and 28, with sera collected on day 49 following previously established methods^4,62^. In the pneumonia with secondary septicaemia model, vaccinated mice (*n* = 8–10/group) were anaesthetised with isoflurane and intranasally inoculated with 1 × 10⁷ CFU of pneumococcal strain TIGR4 in a 50 µl suspension at day 52, while in the sepsis model, mice (*n* = 8–10/group) were injected intraperitoneally with 1 × 10^5^ CFU of TIGR4 pneumococci. Animals were sacrificed either 24 h post-infection or for the survival analysis experiments upon presentation of severe clinical symptoms or at 72 h. Organs were homogenised in 1 ml PBS for both CFU enumeration and flow cytometric analysis. Blood samples were collected via cardiac puncture under terminal anaesthesia and anticoagulated with heparin (100 U/ml; Merck, Gillingham, UK).

### ELISAs and flow cytometry assays

For the protein ELISAs, maxisorp 96-well plates (442404, SLS) were coated with 10 µg/well of individual recombinant proteins diluted in PBS buffer at 4 °C overnight, then after PBS washes were exposed to murine serum diluted 1:100 for 1 h at 37 °C, followed by detection using HRP-conjugated goat anti-mouse IgG (ab6789; Abcam, Cambridge, UK). Antibody recognition of *S. pneumoniae* was assessed using previously described whole cell ELISAs and flow cytometry assays^65^. Briefly, for whole cell ELISAs *S. pneumoniae* were grown to an OD_600_ of approximately 0.4–0.8, washed and resuspended to an OD_600nm_ of 0.4 in PBS, 50 μl/well were added to microtitre plates and incubated overnight at RT before fixation in 4% formaldehyde for 10 min. Plates were washed and incubated with a 1:100 dilution of murine antiserum for 1 h at 37 °C, and using 1: 5000 diluted HRP-conjugated goat anti-mouse IgG (ab6789, lot GR250300-9, abCam) for detection. For the flow cytometry serum deposition assay, live *S. pneumoniae* (1 × 10⁶ CFU) were incubated with 10% mouse serum at 37 °C for 30 min. Antibody binding to the bacterial surface was detected using fluorescently labelled secondary antibodies: anti-mouse IgG-APC (405308, lot B282013, BioLegend). Samples were analysed on a Becton Dickinson FACSVerse cytometer.

### ELISPOT assay for detection of IL-17A-secreting cells

Th17 responses for mice vaccinated with the 22 shortlisted vaccine antigens were assessed using an ELISpot Plus: Mouse IL-17A (HRP) kit (3521-4HPW-2, Mabtech, Sweden) following the manufacturer’s protocol. Briefly, splenocytes were isolated from previously vaccinated mice and resuspended in complete RPMI medium. Cells were seeded into pre-coated ELISPOT plates at a 2 × 10^5^ cells/well density and stimulated with the relevant antigens (10 μg/ml), with medium-only wells serving as negative controls and Concanavalin A (5 μg/ml) as a positive control. After incubation, secreted IL-17A was detected using the kit-supplied biotinylated detection antibody and streptavidin-HRP conjugate, followed by substrate development. Spots corresponding to individual IL-17A–secreting cells were visualised and enumerated using an automated ELISPOT reader (ImmunoSpot). Results were expressed as spot-forming units per 10⁶ splenocytes after background subtraction, and then a ratio between the vaccinated and unvaccinated mice was calculated.

### Confocal microscopy

Fluorescent microscopy was performed using formaldehyde-fixed pneumococci incubated with a 1:100 dilution of vaccinated mouse antiserum and a 1:500 dilution of pneumococcal Omni serum (2438, lot POM1E1, Statens Serum Institut) for 30 min at RT. PBS-washed slides were incubated with a 1:1000 dilution of Alexafluor488-conjugated anti-mouse IgG (A11001, lot 1858182, ThermoScientific) and Alexafluor546-conjugated anti-rabbit IgG (A11010, lot 1811305, ThermoScientific) for 15 min, incubated with a 1:10,000 dilution of 4′,6-diamidino-2-phenylindole (DAPI) in PBS and mounted in DAKO mounting medium (Agilent). Fluorescent images were produced using a Zeiss LSM800 and Zeiss Zen software.

### Epitope mapping and structural model prediction of IgG binding

Peptide microarrays of the antigens included in the vaccine combination were produced by PEPperPRINT (Germany) using the PEPperCHIP® platform technology. Overlapping linear peptides derived from the target antigen with a maximum peptide overlap of shift +1 were synthesised and directly printed onto glass slides, then incubated with human IVIG (Privigen, CSL Behring) or mouse anti-sera, followed by incubation with appropriate secondary antibodies. Resulting spot patterns were analysed to identify linear and conformational epitopes corresponding to the tested samples. Three-dimensional structures of the target proteins were predicted using the AlphaFold2 algorithm implemented in the Benchling software platform using default parameters. The resulting models were examined to identify surface-exposed and accessible regions, and integrated with the epitope mapping data. Regions yielding positive signals in both naturally immunised human and murine serum samples were highlighted on the structural models, corresponding to putative IgG-binding epitopes.

### Statistical analysis

Statistical analyses were conducted using Prism 8 (Graph Pad, San Diego, USA). Unless otherwise stated, data are presented as means, and error bars represent standard deviations. Due to the relatively small sample size, all the data have been analysed as non-parametric. Depending on the number of groups compared, either the Mann–Whitney *U* test or the Kruskal–Wallis test with Dunn’s correction has been used to compare ELISA, Elispot, serum deposition assay data and CFU after infection data. All statistical analyses were performed using two-tailed tests.

Mouse survival data were analysed using Kaplan–Meier survival curves, and statistical differences between groups were assessed using the Log-rank (Mantel–Cox) test. Correlation analyses were performed using Spearman’s rank correlation to calculate the correlation coefficient (*R*) and *P* values.

### Reporting summary

Further information on research design is available in the Nature Portfolio Reporting Summary linked to this article.

## Supplementary information

Supplementary Information

Description of Additional Supplementary File

Supplementary Data 1

Supplementary Data 2

Supplementary Data 3

Supplementary Data 4

Supplementary Data 5

Reporting Summary

Transparent Peer Review file

## Source data

Source data

## Data Availability

All data generated in this study have been deposited in the Figshare database under accession code 10.6084/m9.figshare.31843885. All raw data are provided in the Supplementary Data and Source data files. Source data are provided with this paper.
