# Supplementary material for: High-throughput antigen discovery using Functional Genomic Vaccinology (FGV) identifies protective Streptococcus pneumoniae vaccine candidates
Source: Nat Commun. 2026 Jul 21;17:8867. doi: 10.1038/s41467-026-75848-2 (PMC13500534; doi:10.1038/s41467-026-75848-2)
Supplement: Supplementary file 1 [file 41467_2026_75848_MOESM1_ESM.pdf]

Supplementary figures

PspA

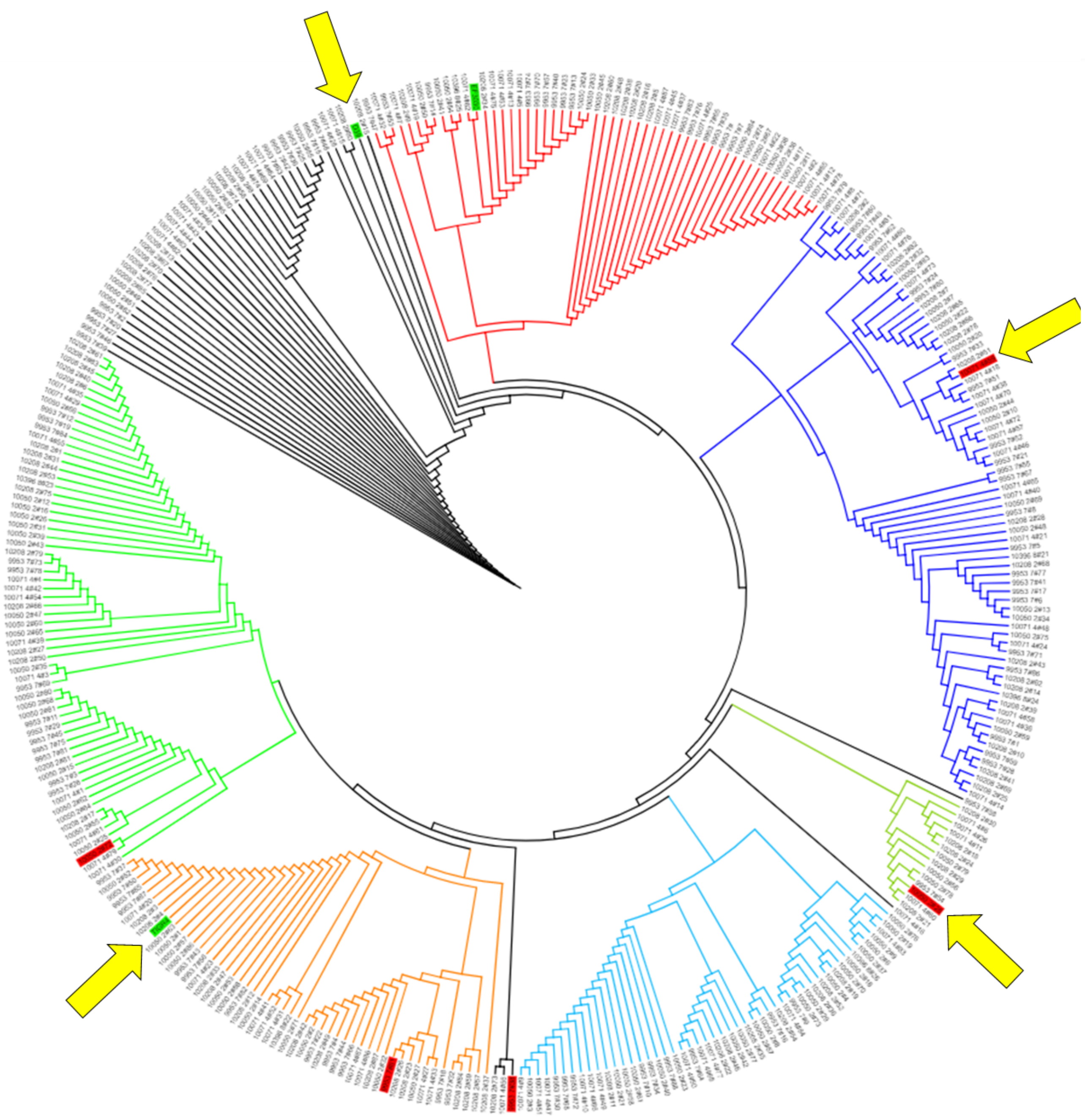

PspC

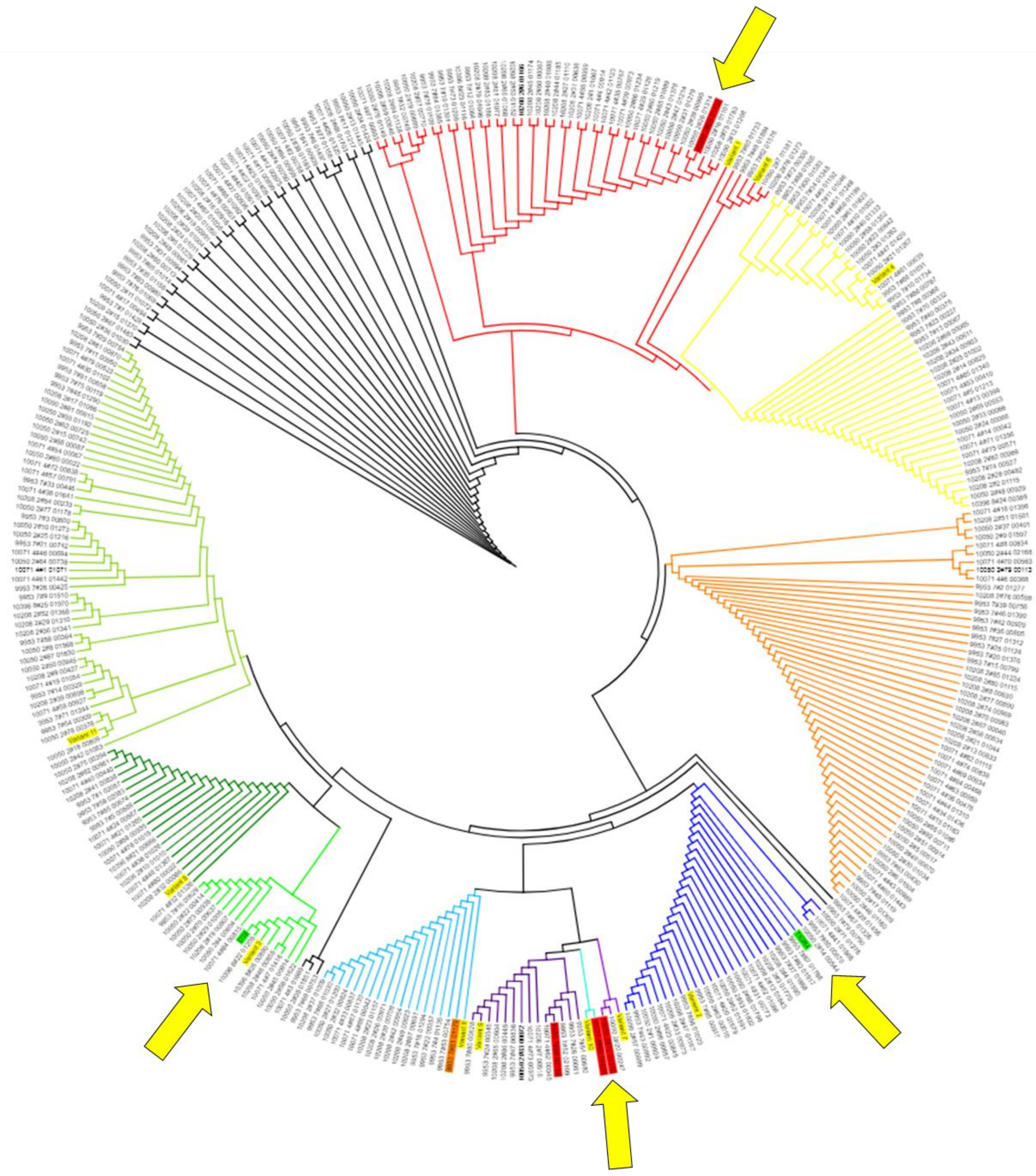

Fig.S1: Phylogenetic tree of PspA and PspC protein sequences. Diversity of protein sequences across a collection of 349 clinical isolates have been shown as phylogenetic trees. Four strains have been selected to try to represent the antigens' variability. Strain selected were TIGR4, D39, 10050 2#28 and 10071 4#59 (indicated by yellow arrows).

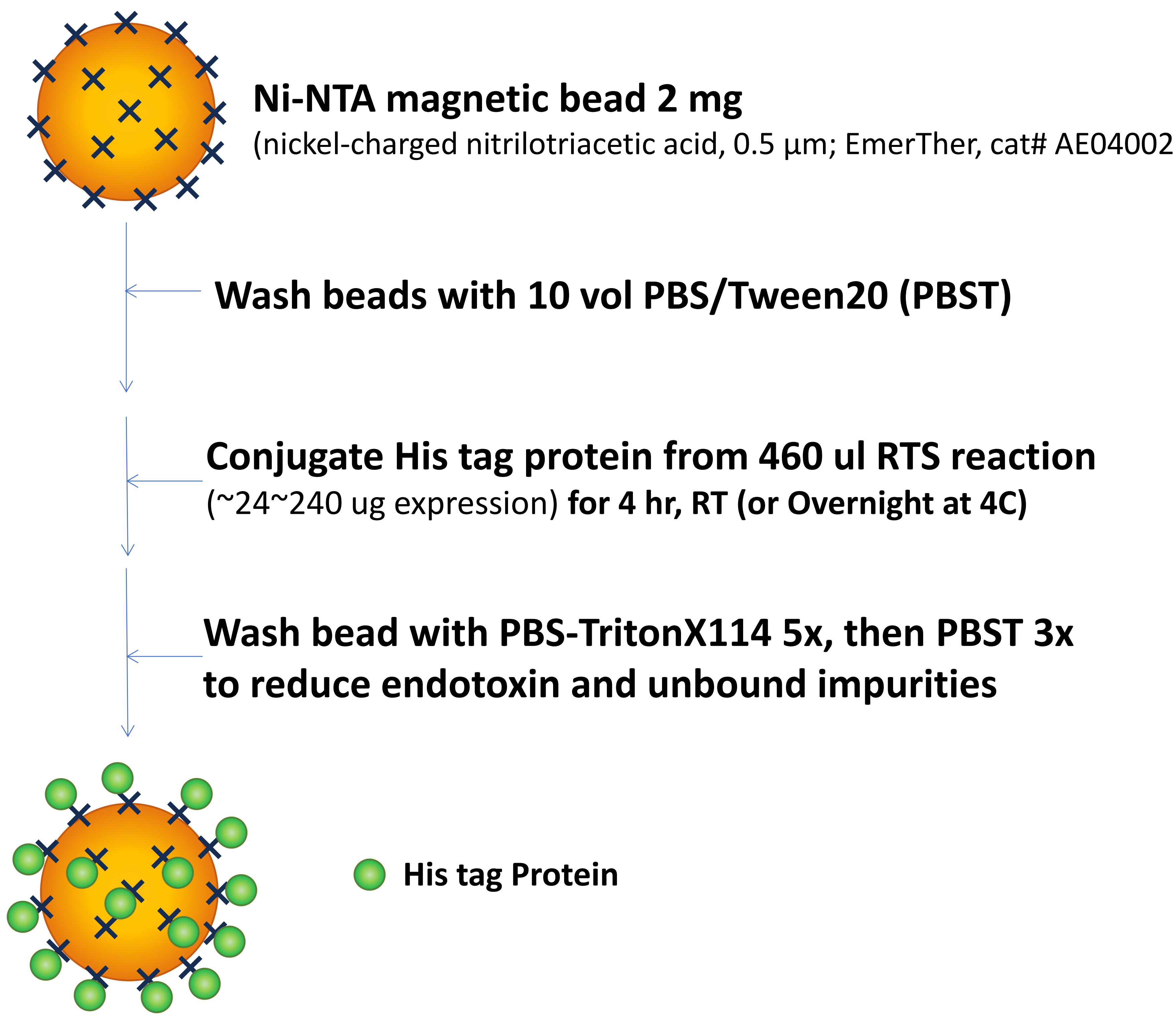

Fig.S2: Antigen capture on beads schematic protocol and binding mechanism

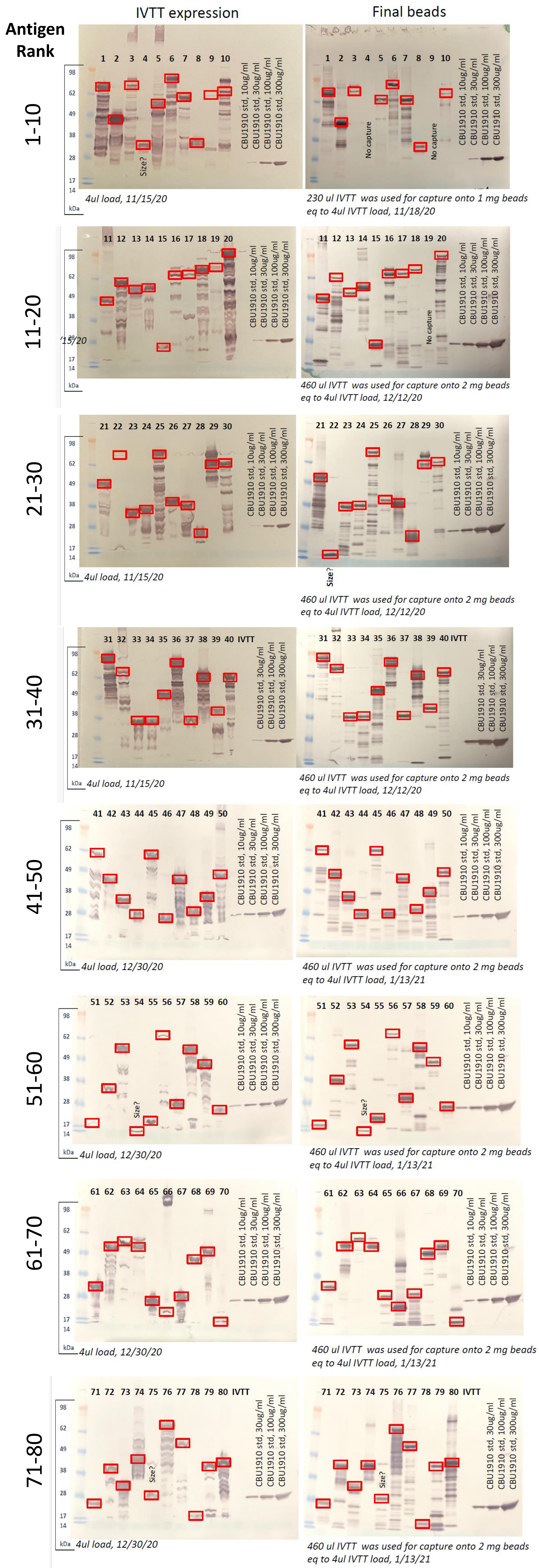

Rank	Ag name	Size [kDa]	ug/ml	vol (ml)	ug
1	SP_0641a	81.6	117	0.46	54
2	SP_2216	40.2	373	0.46	172
3	pspC-D39	73.0	49	0.46	23
4	pspA-10050-2#28	70.8	low expression, no capture		
5	pspA-10071-4#59	62.4	92	0.46	42
6	SP_1174	88.1	161	0.46	74
7	SP_1732	72.5	202	0.46	93
8	SP_0107	18.3	79	0.46	18
9	SP_1518	60.6	low expression, no capture		
10	pspC-10071-4#59	73.3	39	0.46	21
11	SP_0785	43.9	81	0.92	74
12	SP_1527	68.3	18	0.92	16
13	SP_2063	76.5	25	0.92	23
14	SP_0374	51.0	176	0.92	162
15	SP_1604	17.4	171	0.92	157
16	SP_0641c	80.2	36	0.92	33
17	SP_0641b	70.6	23	0.92	21
18	SP_0498b	95.9	21	0.92	20
19	pspA-TIGR4	78.4	low expression, no capture		
20	SP_0648c	102.6	19	0.92	18
21	SP_1923	51.8	106	0.92	98
22	pspC-TIGR4	72.2	135	0.92	124
23	SP_1942	34.5	60	0.92	55
24	SP_1650	31.7	52	0.92	47
25	SP_0057b	79.2	45	0.92	41
26	SP_1032	34.7	38	0.92	35
27	SP_1937	35.0	360	0.92	332
28	SP_0446	17.4	425	0.92	391
29	SP_0057a	61.4	52	0.92	48
30	SP_1891	69.0	41	0.92	38
31	SP_0498a	82.5	59	0.92	54
32	SP_0368c	78.9	66	0.92	61
33	SP_1872	31.8	55	0.92	51
34	SP_0845	35.2	45	0.92	42
35	SP_1479	50.9	150	0.92	138
36	SP_0648a	81.7	81	0.92	74
37	SP_0981	31.8	97	0.92	89
38	SP_0268a	61.2	84	0.92	77
39	SP_2239	39.8	56	0.92	52
40	SP_0366	69.5	72	0.92	66
IVTT Ctrl	none	na	na	0.92	na

Rank	Ag name	Size [kDa]	ug/ml	vol (ml)	ug
41	SP_0930	66.2	20	0.92	19
42	SP_1368	46.6	167	0.92	154
43	SP_2223	30.4	229	0.92	210
44	SP_0423_A5	17.7	163	0.92	150
45	SP_0807	63.3	51	0.92	47
46	SP_0423_B5	17.7	293	0.92	270
47	SP_0749	39.4	149	0.92	137
48	SP_0629	23.8	124	0.92	114
49	SP_1854	36.9	328	0.92	301
50	SP_0453	54.7	88	0.92	81
51	SP_2102	11.3	25	0.92	23
52	SP_2112	36.1	183	0.92	169
53	3_G7	59.4	33	0.92	30
54	pspC-10050-2#28*	70.8	14	0.92	12
55	SP_0053	17.8	294	0.92	270
56	SP_2099	81.3	14	0.92	12
57	SP_2218	27.2	302	0.92	278
58	SP_0648b	57.2	116	0.92	107
59	SP_2108	43.3	36	0.92	33
60	SP_0191	18.5	96	0.92	88
61	SP_1560	24.9	38	0.92	35
62	SP_1954	49.0	120	0.92	111
63	SP_1161	62.4	19	0.92	17
64	SP_0368a	52.5	135	0.92	124
65	SP_1394	26.4	29	0.92	27
66	SP_1572	19.6	309	0.92	284
67	SP_1500	27.8	43	0.92	40
68	SP_0043	49.4	206	0.92	190
69	SP_0877	71.5	105	0.92	96
70	SP_0928	14.3	83	0.92	76
71	SP_0659	18.0	35	0.92	32
72	SP_0054	39.9	114	0.92	105
73	SP_1400	29.3	57	0.92	53
74	SP_0097	38.9	197	0.92	181
75	SP_1890*	53.7	42	0.92	39
76	SP_0369	75.5	110	0.92	101
77	SP_0368b	58.9	87	0.92	80
78	SP_1545	12.7	28	0.92	26
79	SP_0616	45.1	48	0.92	44
80	SP_1981	43.5	310	0.92	285
IVTT	none				

size questionable possibly due to degradation or incomplete expression

Fig.S3: Antigen capture on beads assessment and quantification

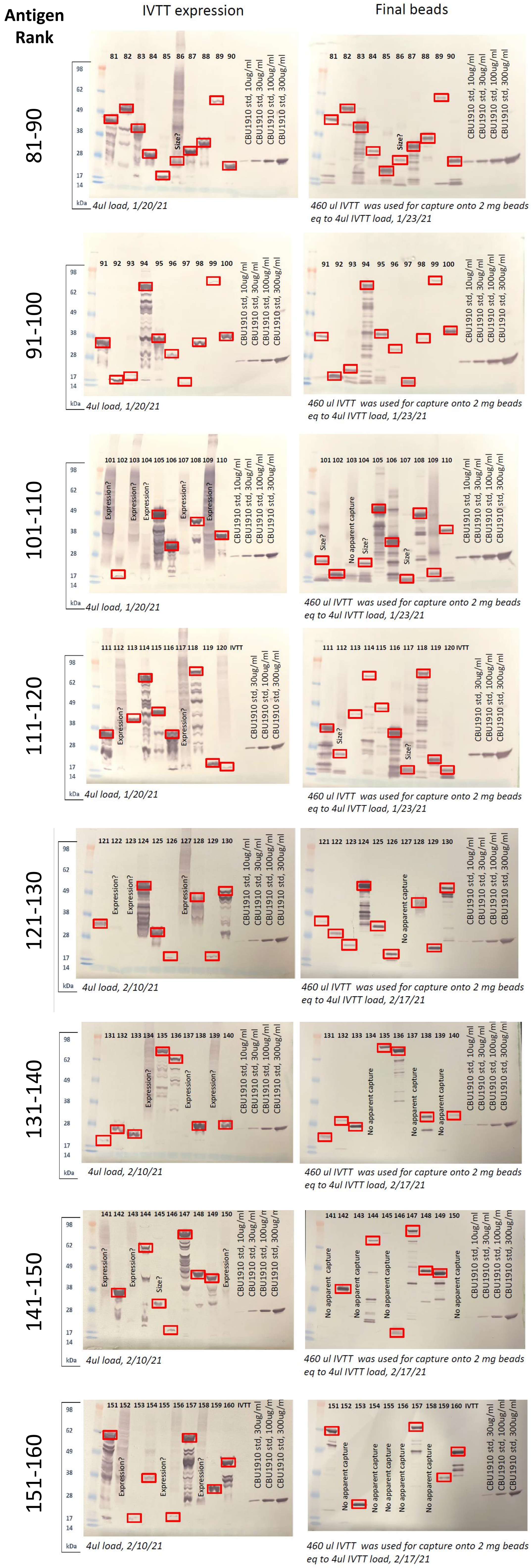

Fig.S3 (continued)

Rank	Ag name	Size [kDa]	ug/ml	vol (ml)	ug
81	SP_0872	43.0	45	0.92	42
82	SP_2228	54.1	135	0.92	124
83	SP_2096	41.4	333	0.92	307
84	SP_0148	26.6	23	0.92	21
85	SP_1680	16.5	256	0.92	236
86*	SP_1689	32.3	25	0.92	23
87	SP_1363	33.4	275	0.92	253
88	SP_1045	32.3	67	0.92	62
89	SP_0042	78.9	16	0.92	15
90	SP_0746	21.6	134	0.92	123
91	SP_1069	33.3	18	0.92	17
92	SP_1714	13.3	205	0.92	188
93	SP_1802	20.9	43	0.92	40
94	SP_0894	83.3	48	0.92	44
95	SP_0660	34.2	34	0.92	31
96	SP_2084	29.0	12	0.92	11
97	SP_1411	14.0	43	0.92	40
98	SP_1002	30.7	10	0.92	9
99	SP_1219	90.4	10	0.92	9
100	SP_1999	37.0	120	0.92	110
101*	SP_2110	30.8	19	0.92	18
102	SP_1558	14.1	98	0.92	90
103	SP_1649	31.0	No apparent capture		
104*	SP_2085	31.6	20	0.92	19
105	SP_1213	46.6	464	0.92	427
106	SP_2034	31.6	374	0.92	344
107*	SP_0873	40.6	41	0.92	37
108	SP_1083	46.8	119	0.92	109
109	SP_1012	18.6	20	0.92	18
110	SP_0284	36.5	30	0.92	28
111	SP_2188	31.9	237	0.92	218
112*	SP_1398	32.3	14	0.92	13
113	SP_1683	45.1	8	0.92	7
114	SP_0585	82.4	11	0.92	10
115	SP_1128	47.7	13	0.92	12
116	SP_2016	31.9	248	0.92	228
117*	SP_1893	43.7	55	0.92	51
118	SP_1152b	88.9	42	0.92	38
119	SP_0409	20.0	78	0.92	72
120	SP_0049	18.6	45	0.92	42
IVTT Ctrl	none				

Rank	Ag name	Size [kDa]	ug/ml	vol (ml)	ug
121	SP_0771	26.8	10	0.92	9
122	SP_0198	13.6	34	0.92	31
123	SP_1786	16.5	20	0.92	19
124	SP_0690	43.9	635	0.92	584
125	SP_0149	27.9	95	0.92	87
126	SP_1218	13.5	59	0.92	55
127*	SP_1033	37.3	no capture		
128	SP_0084	38.5	28	0.92	25
129	SP_1519	18.0	199	0.92	183
130	SP_1489	43.8	97	0.92	89
131	SP_0744	17.1	54	0.92	50
132	SP_1648	26.4	30	0.92	28
133	SP_0745	23.0	195	0.92	179
134*	SP_1034	36.9	no capture		
135	SP_0878	84.4	106	0.92	97
136	SP_1247a	75.9	302	0.92	278
137*	SP_2052	31.9	no capture		
138	SP_0987	26.5	110	0.92	101
139*	SP_1945	22.7	no capture		
140	SP_0282	33.3	12	0.92	11
141*	SP_0547	22.3	no capture		
142	SP_0285	37.3	270	0.92	248
143*	SP_1412	28.8	no capture		
144	SP_0908	78.0	35	0.92	32
145*	SP_0274a	82.5	no capture		
146	SP_1223	9.2	40	0.92	37
147	SP_0568	97.1	134	0.92	123
148	SP_0092	48.7	174	0.92	160
149	SP_1226	49.4	328	0.92	302
150*	SP_1111	27.9	no capture		
151	SP_0045b	68.9	225	0.92	207
152*	SP_0324	28.5	no capture		
153	SP_1996	16.5	287	0.92	264
154*	SP_1688	30.5	no capture		
155*	SP_1116	43.1	no capture		
156*	SP_1677	23.5	no capture		
157	SP_0045a	67.7	135	0.92	125
158*	SP_1870	35.0	no capture		
159	SP_1084	31.5	41	0.92	37
160	SP_2121	47.2	323	0.92	298
IVTT Ctrl	none				

**size questionable possibly due to degradation or incomplete expression*

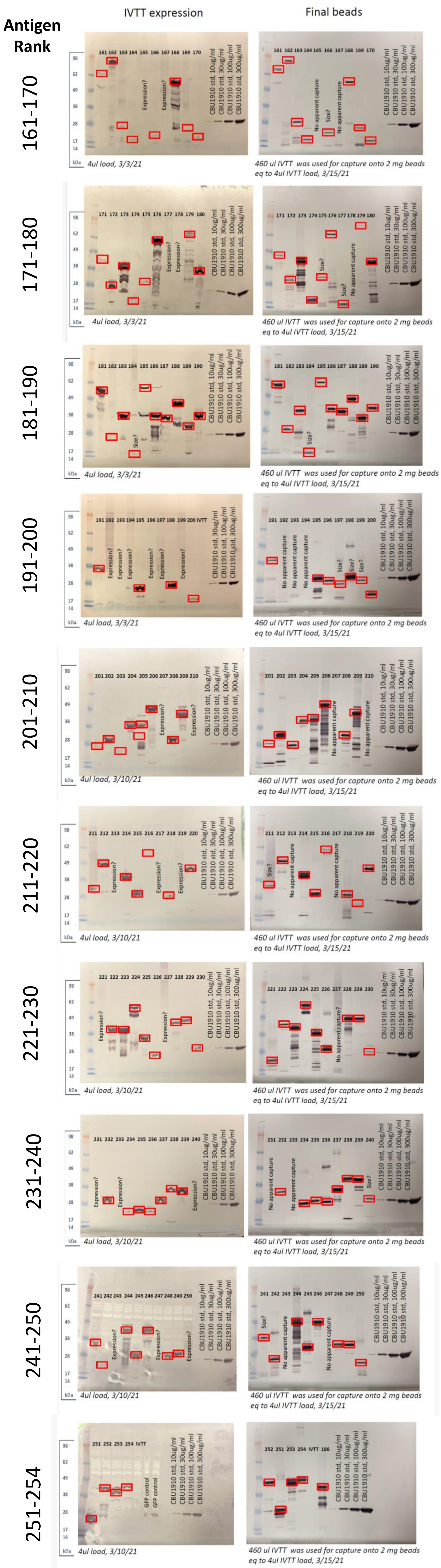

Fig.S3 (continued)

Rank	Ag name	Size [kDa]	ug/ml	vol (ml)	ug
161	SP_0274b	78.4	42	0.92	38
162	SP_2143	97.5	45	0.92	41
163	SP_1399	33.6	50	0.92	46
164	SP_2086	29.8	43	0.92	39
165	SP_1533	18.7	no capture		
166*	SP_1625	22.4	36	0.92	33
167	SP_1067	45.0	no capture		
168	SP_2169	53.1	59	0.92	55
169	SP_1388	29.0	35	0.92	32
170	SP_2029	10.9	40	0.92	37
171	SP_0799	48.8	14	0.92	13
172	SP_0963	27.5	22	0.92	20
173	SP_1888	39.1	266	0.92	245
174	SP_1882	9.0	108	0.92	100
175*	SP_0159	46.2	20	0.92	19
176	SP_0517	66.8	40	0.92	37
177*	SP_0638	28.7	45	0.92	41
178	SP_0288	25.3	no capture		
179	SP_1833	74.1	10	0.92	9
180	SP_1025	36.9	293	0.92	270
181	SP_1739	58.7	129	0.92	118
182	SP_1889	33.9	57	0.92	53
183	SP_0856	37.4	114	0.92	105
184*	SP_0704	27.9	53	0.92	48
185	SP_0730	65.0	59	0.92	55
186	SP_0095	36.1	101	0.92	93
187	SP_0927	33.2	202	0.92	186
188	SP_0762	43.6	195	0.92	180
189	SP_1946	31.6	232	0.92	213
190	SP_0047	37.4	123	0.92	113
191	SP_0647	54.0	19	0.92	18
192	SP_2170	29.5	no capture		
193	SP_1522	22.6	no capture		
194	SP_1859	29.6	no capture		
195	SP_1638	23.8	167	0.92	153
196	SP_1903	19.5	44	0.92	40
197*	SP_0858	25.2	33	0.92	30
198*	SP_1664	19.7	272	0.92	250
199*	SP_0859	33.8	34	0.92	32
200	SP_1626	9.8	112	0.92	103
201	SP_2171	25.7	13	0.92	12
202	SP_1266	31.5	244	0.92	225
203	SP_1041	22.3	33	0.92	31
204	SP_0447	37.0	33	0.92	31
205	SP_0920	41.3	115	0.92	106
206	SP_1852	54.2	174	0.92	160
207	pspA-D39	64.7	no capture		
208	SP_1112	30.7	54	0.92	50
209	SP_1389	42.4	186	0.92	171
210	SP_0751	35.0	no capture		
211	SP_2109	47.9	22	0.92	20
212	SP_0150	50.3	63	0.92	58
213	SP_1529	58.2	no capture		
214	SP_2012	36.9	164	0.92	151
215	SP_1804	22.2	130	0.92	120
216	SP_0729	72.6	30	0.92	28
217	SP_0848	35.0	no capture		
218	SP_0090	35.1	58	0.92	53
219	SP_0283	29.4	17	0.92	15
220	SP_0422	45.2	124	0.92	114
221	SP_1808	20.5	39	0.92	36
222	SP_1152a	44.9	34	0.92	31
223	SP_1386	36.7	195	0.92	179
224	SP_1247b	53.8	92	0.92	85
225	SP_1821	36.6	146	0.92	134
226	SP_0860	23.5	124	0.92	114
227	SP_2236	48.5	no capture		
228	SP_2224	47.0	137	0.92	126
229	SP_1472	45.5	109	0.92	100
230	SP_1731	27.7	16	0.92	15
231	SP_0152	25.3	no capture		
232	SP_0576	30.7	31	0.92	29
233	SP_0719	20.5	no capture		
234	SP_1583	21.0	81	0.92	74
235	SP_0766	22.1	50	0.92	46
236	SP_0992	17.9	74	0.92	68
237	SP_1396	27.7	203	0.92	187
238	SP_0027	35.4	122	0.92	113
239	SP_1887	33.9	151	0.92	139
240*	SP_0091	33.8	34	0.92	31
241*	SP_0230	48.0	33	0.92	30
242	SP_2219	29.0	64	0.92	59
243	SP_1502	23.4	no capture		
244	SP_1978	45.8	296	0.92	273
245	SP_0058	26.2	302	0.92	278
246	SP_0932	46.2	157	0.92	145
247	SP_2174	8.7	no capture		
248	SP_1035	29.0	80	0.92	73
249	SP_1584	28.8	104	0.92	95
250	SP_1720	22.6	36	0.92	33
251	SP_1871	27.5	119	0.92	110
252	SP_1521	45.4	101	0.92	93
253	SP_1081	46.1	216	0.92	198
254	SP_0450	45.8	91	0.92	84
IVTT Ctrl	none				

size questionable possibly due to degradation or incomplete expression

A)

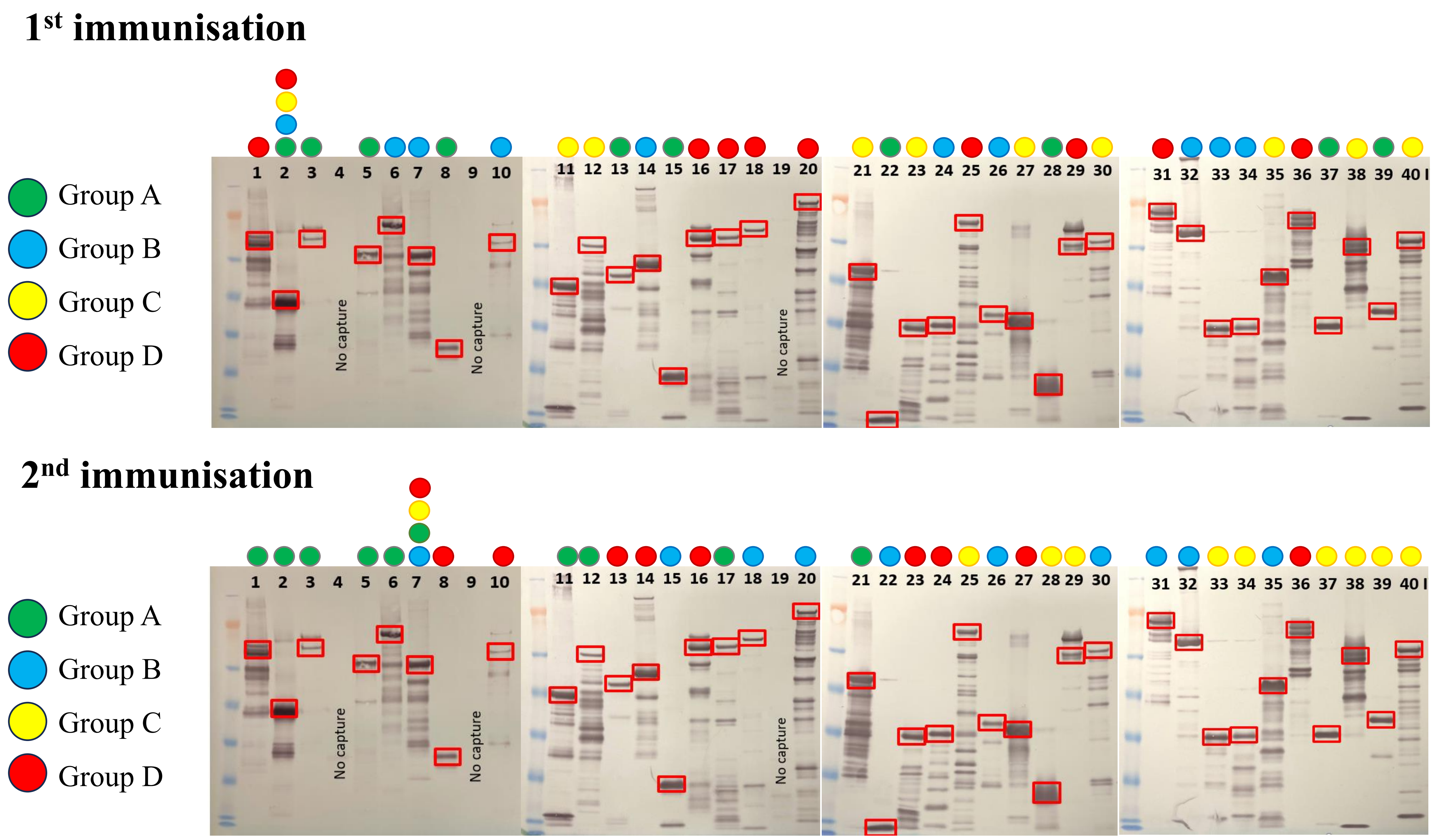

B)

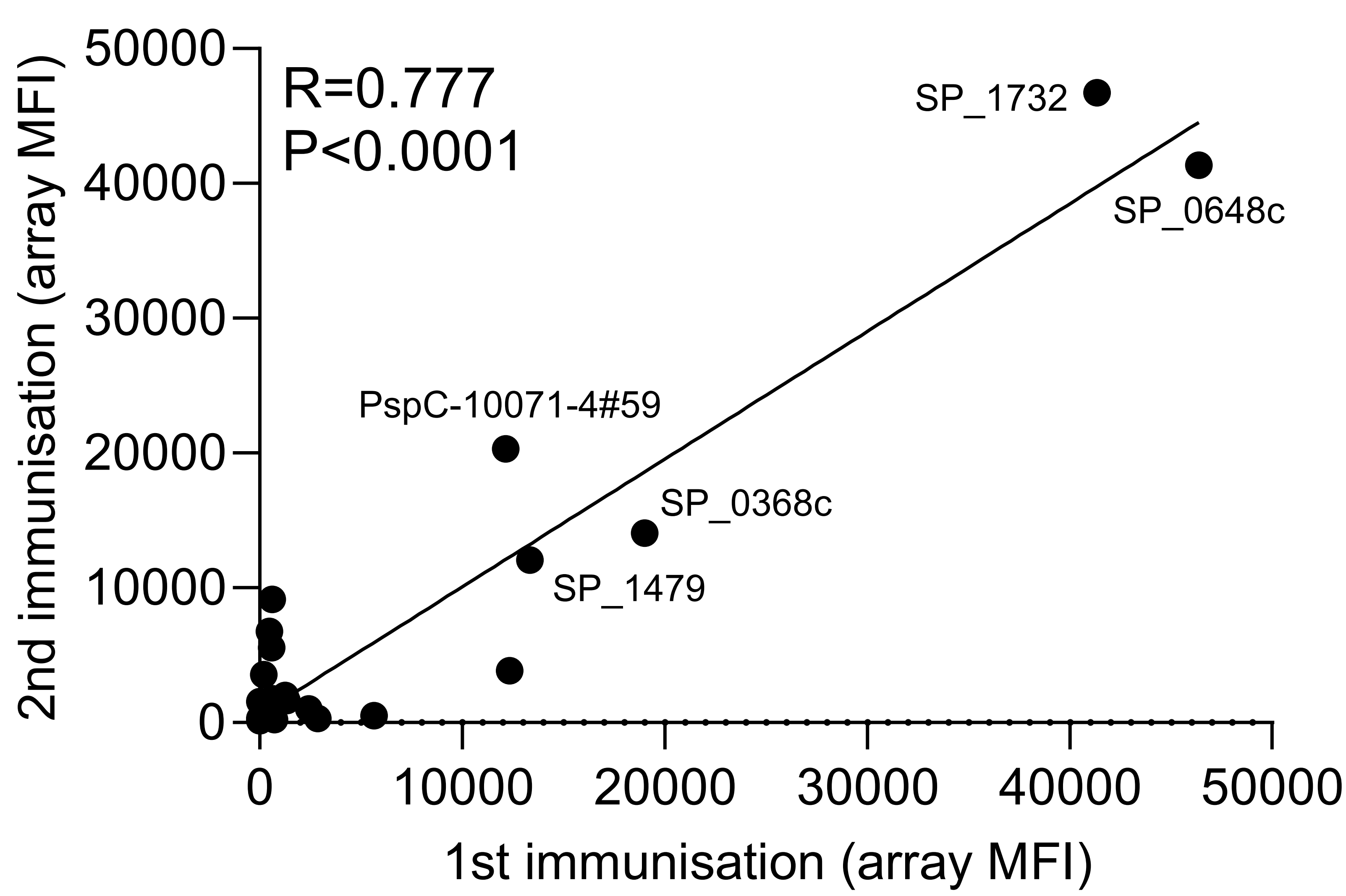

Fig.S4:. (A) Individual protein-beads antigens used for mouse immunisation are shown by His-tag western blot. Two independent vaccination experiments each including four mice groups (Group A to D) have been performed using the same 37 antigens. 9-10 antigens have included in each group and the protein combinations used have been shuffled in the second experiment (see coloured dots indicating what each group contains). (B) A linear correlation between the response observed in the two experiments is shown. Each dot represents a single protein; linear regression was calculated for all correlations and shown as a black dotted line; R and P-values calculated with Spearman correlation (two-tailed) are reported in the top left corner.

A)

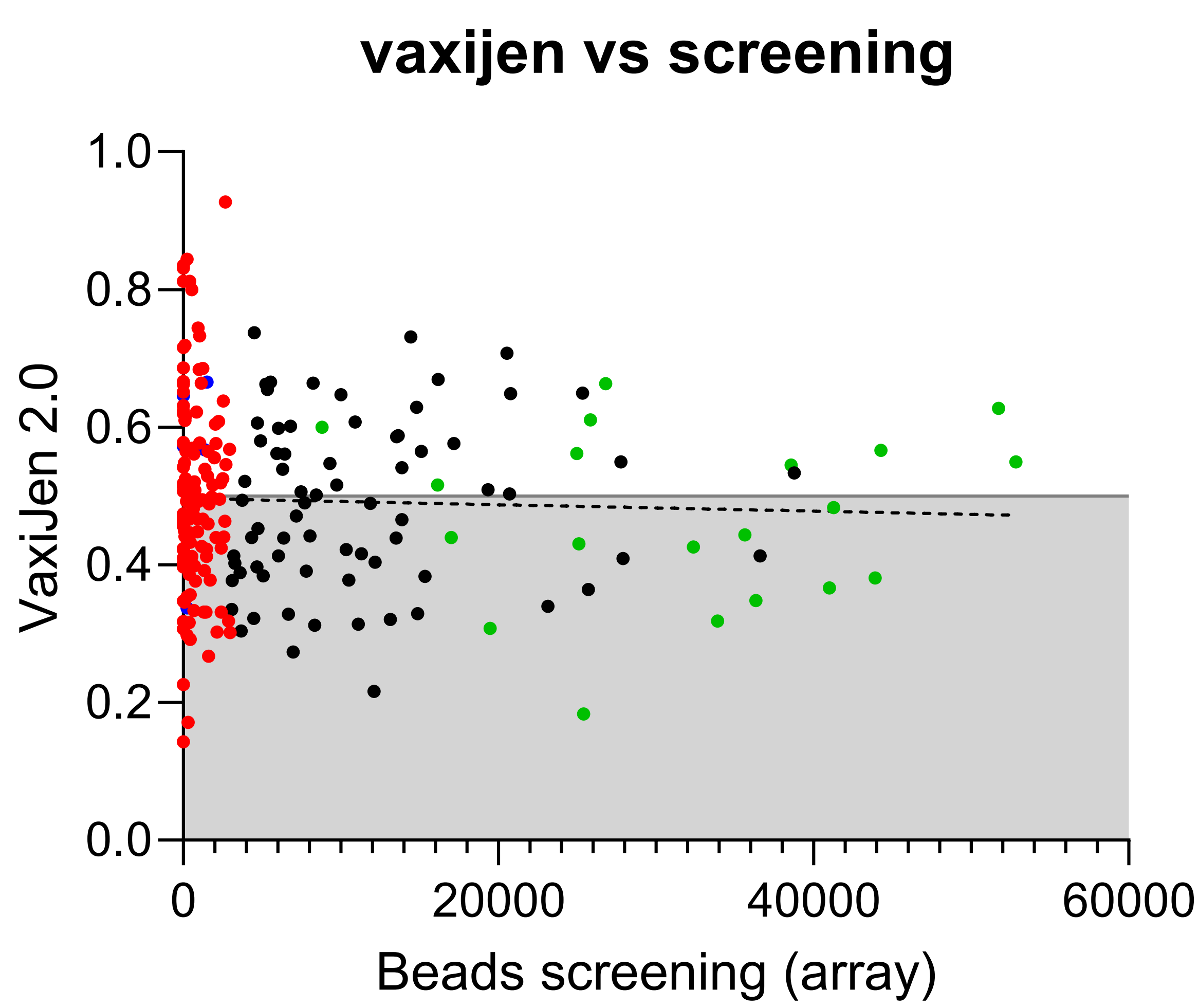

B)

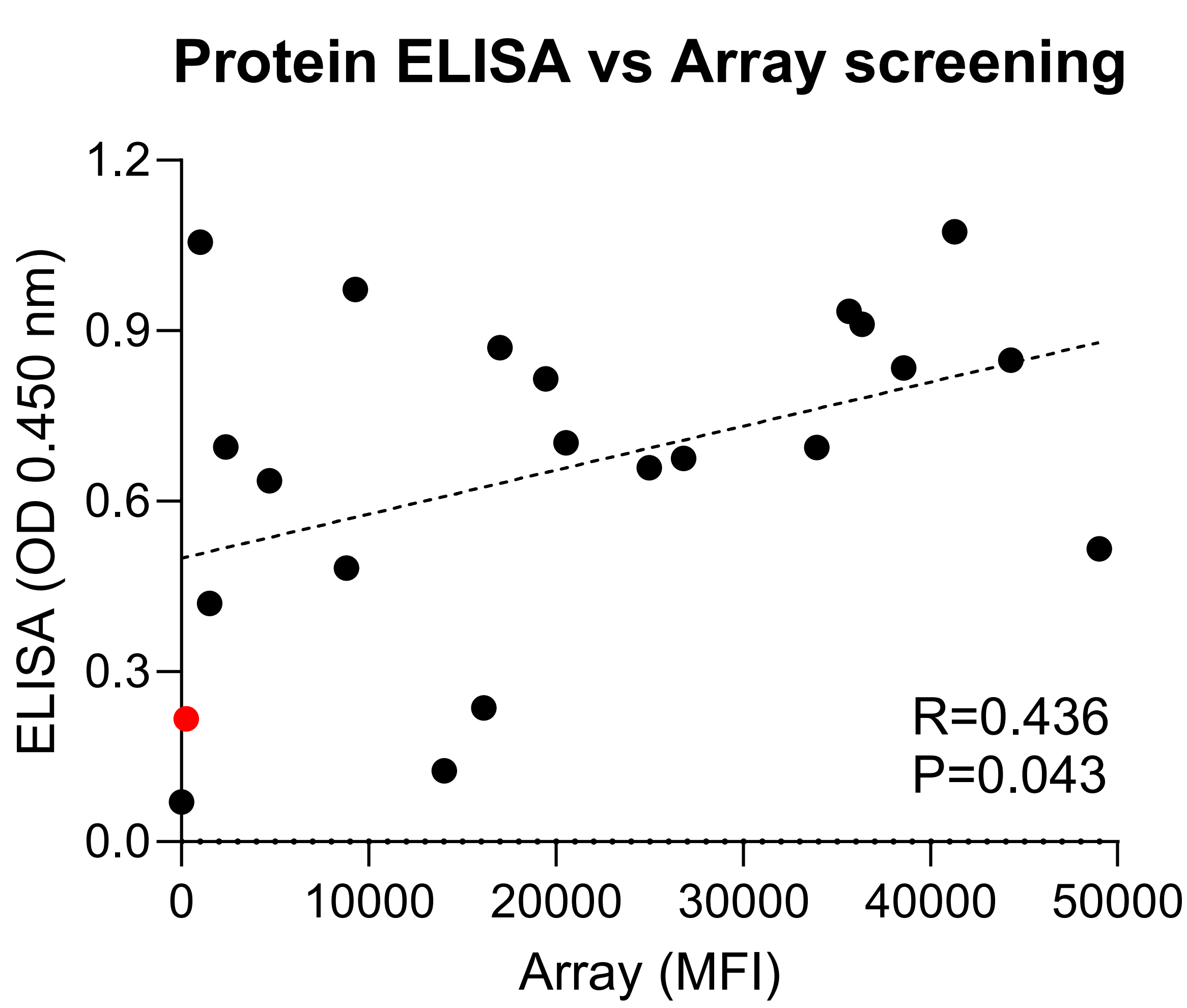

Fig.S5: Correlations of array screening data. (A) Correlation between array screening data and Vaxijen 2.0 antigenicity prediction scores is shown. Antigens shortlisted for recombinant proteins production are shown in green and screening negative proteins in red. (B) Array screening data have been also correlated to level of IgG produced from the shortlisted antigens produced as recombinant proteins and tested in immunisations (measured by protein ELISA) . Negative control protein is shown in red (SP_0027). Each dot represents a single protein; linear regression was calculated for all correlations and shown as a black dotted line; R and P-values are reported in the bottom right corner.

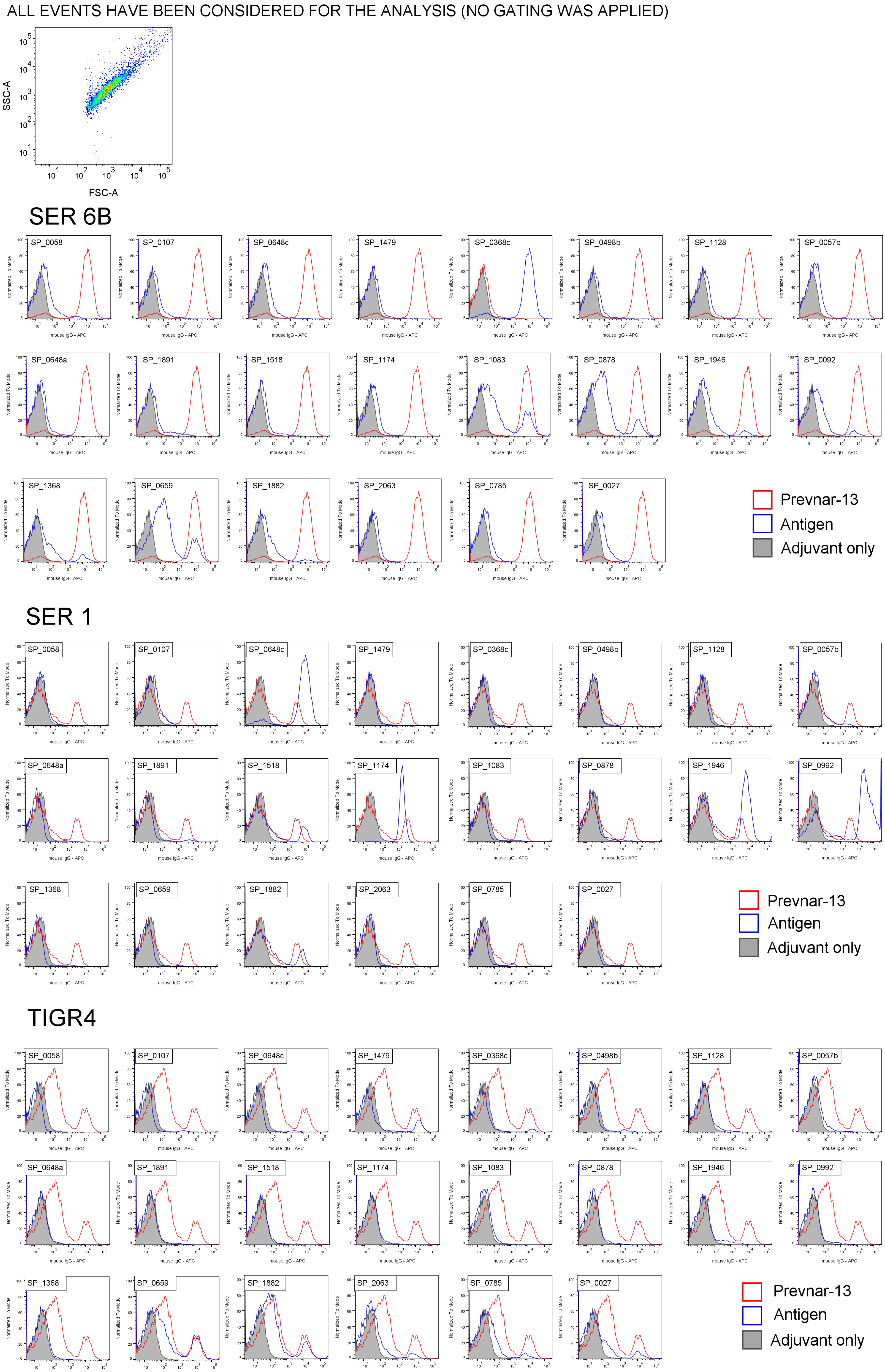

Fig.S6: Serum deposition measured by flow cytometry on pneumococcal surface. Individual sera representative data are shown, the assay has been performed twice with two technical replicas per sample tested.

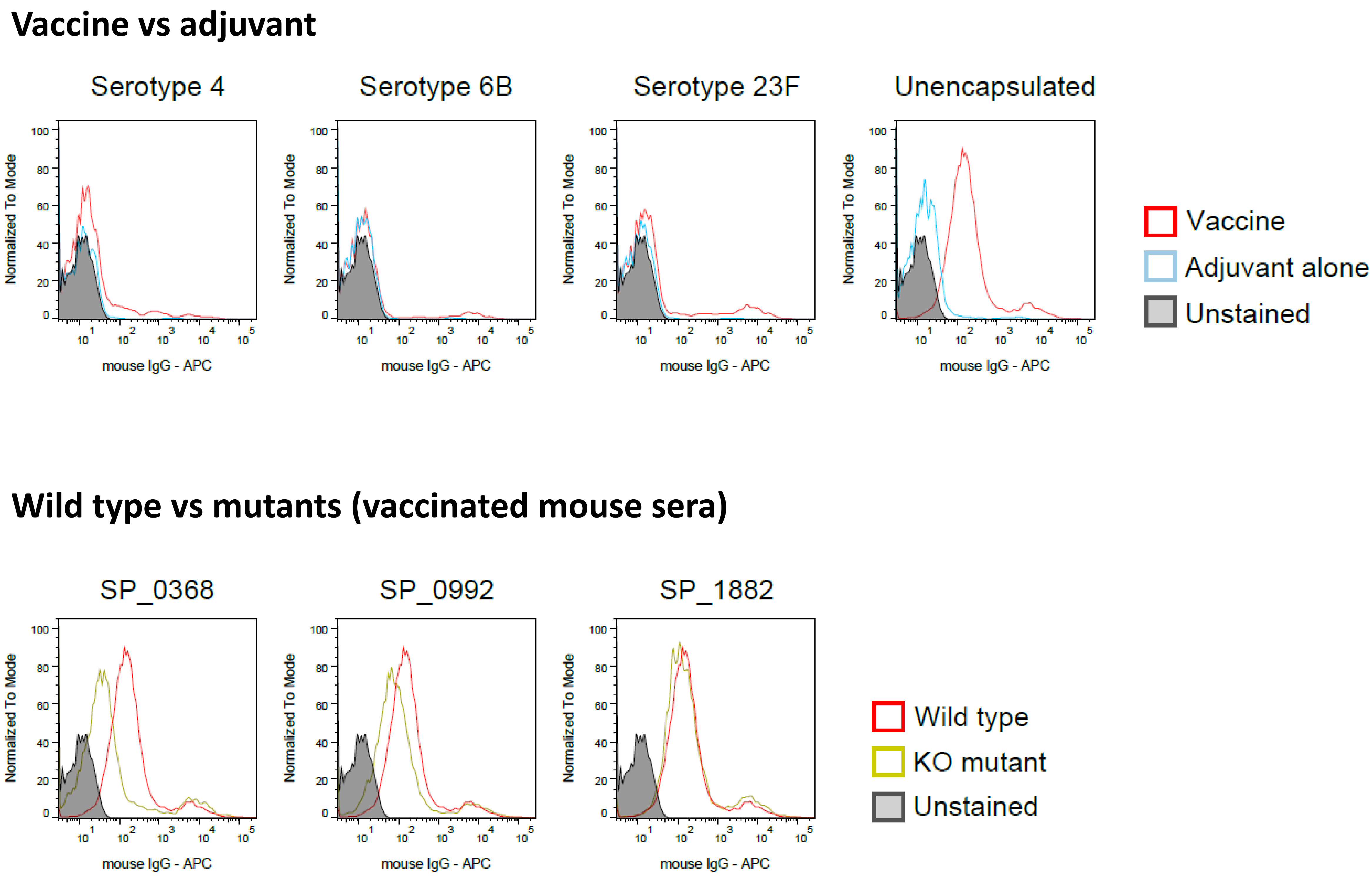

Fig.S7: IgG raised by combination vaccine binding to pneumococcus. Serum deposition measured by flow cytometry on pneumococcal surface. Pooled mouse sera representative data are shown, the assay has as been performed twice with two technical replicas per sample tested.
