# Supplementary material for: High-throughput antigen discovery using Functional Genomic Vaccinology (FGV) identifies protective Streptococcus pneumoniae vaccine candidates
Source: Nat Commun. 2026 Jul 21;17:8867. doi: 10.1038/s41467-026-75848-2 (PMC13500534; doi:10.1038/s41467-026-75848-2)
Supplement: Supplementary file 2 [file 41467_2026_75848_MOESM2_ESM.pdf]

Supplementary figures

Supplementary data files

Supplementary data 1

Sheet 1: Pneumococcal pangenome
2048 pneumococcal proteins are listed with info about IgG binding measured in humans sera and sequence conservation analysis. Red highlighted cells indicate the values that are matching our criteria for antigen selection.

Sheet 2: Array selection
All the proteins included in the array are listed and the criteria for selection highlighted. Big antigens divided in multiple domains are listed, as well as the proteins identified from other studies and the ones with multple variants (PspA,PspC).

Sheet 3: Primers
Primers designed for the polypeptides included in the array.

Sheet 4: Screening steps
The table summarises the different screening steps.

Sheet 5: Array validation samples
The heatmap shows the IgG signal measured using the array against control samples (Healthy human sera and IVIG) and previously published data (colonised mouse sera)

Supplementary data 2

Beads-linked antigens immunisation doses
The estimated concentration, doses and antigen combinations used for immunisaton of the first 4 groups are reported (37 antigens). 3 different doses have been tested: 50, 100 and 400 ng. Colour groups indicate antigens used in combination.

Supplementary data 3

Screening immunisation doses
Protein concentration and combinations for the immunisations of the remaining screening candidates.

Supplementary data 4

Sheet 1: Screening array results
Heatmap showing the IgG response measured by protein array for the antigens used in the screening. Green cells indicate immunisation groups where less than 5 mice were available for the array test.

Sheet 2: Candidates features
The table lists the antigens included in all the different steps of the screeing and the data used for the selection, obtained both from the array and other methodologies (ELISA, Serum deposition, Elispot).

Supplementary data 5

Sheet 1: Summary of epitope array results
A report of the hotspot binding regions identified on the epitope array probed with human IVIG for the top four protein vaccine candidates

Sheet 2: Epitope mapping results

Median fluorescence intensity measured for the Streptococcus Pneumoniae Peptide Microarray probed with Human IVIG. The intensity plot is based on the corrected intensity values and sorted from the N-terminus of >SP_0368c to the C-terminus of >SP_1882.
