# Supplementary material for: High-throughput antigen discovery using Functional Genomic Vaccinology (FGV) identifies protective Streptococcus pneumoniae vaccine candidates
Source: Nat Commun. 2026 Jul 21;17:8867. doi: 10.1038/s41467-026-75848-2 (PMC13500534; doi:10.1038/s41467-026-75848-2)
Supplement: Supplementary file 3 [file 41467_2026_75848_MOESM8_ESM.pdf]

nature portfolio

Corresponding author(s): Giuseppe Ercoli

Last updated by author(s): Nov 28, 2025

Reporting Summary

Nature Portfolio wishes to improve the reproducibility of the work that we publish. This form provides structure for consistency and transparency in reporting. For further information on Nature Portfolio policies, see our Editorial Policies and the Editorial Policy Checklist.

Statistics

For all statistical analyses, confirm that the following items are present in the figure legend, table legend, main text, or Methods section.

n/a	Confirmed	
☐	☒	The exact sample size (*n*) for each experimental group/condition, given as a discrete number and unit of measurement
☐	☒	A statement on whether measurements were taken from distinct samples or whether the same sample was measured repeatedly
☐	☒	The statistical test(s) used AND whether they are one- or two-sided<br>*Only common tests should be described solely by name; describe more complex techniques in the Methods section.*
☒	☐	A description of all covariates tested
☐	☒	A description of any assumptions or corrections, such as tests of normality and adjustment for multiple comparisons
☐	☒	A full description of the statistical parameters including central tendency (e.g. means) or other basic estimates (e.g. regression coefficient) AND variation (e.g. standard deviation) or associated estimates of uncertainty (e.g. confidence intervals)
☐	☒	For null hypothesis testing, the test statistic (e.g. *F*, *t*, *r*) with confidence intervals, effect sizes, degrees of freedom and *P* value noted<br>*Give P values as exact values whenever suitable.*
☒	☐	For Bayesian analysis, information on the choice of priors and Markov chain Monte Carlo settings
☒	☐	For hierarchical and complex designs, identification of the appropriate level for tests and full reporting of outcomes
☐	☒	Estimates of effect sizes (e.g. Cohen's *d*, Pearson's *r*), indicating how they were calculated

Our web collection on statistics for biologists contains articles on many of the points above.

Software and code

Policy information about availability of computer code

Data collection	Mapix was used to scan the protein array
Data analysis	Mapix was used to quantify the protein array scans, FlowJo has been used to analyse flow cytometry data, Benchling has been used to create the alphafold models, GraphPad PRISM has been used to create graphs.

For manuscripts utilizing custom algorithms or software that are central to the research but not yet described in published literature, software must be made available to editors and reviewers. We strongly encourage code deposition in a community repository (e.g. GitHub). See the Nature Portfolio guidelines for submitting code & software for further information.

Data

Policy information about availability of data

All manuscripts must include a data availability statement. This statement should provide the following information, where applicable:

- Accession codes, unique identifiers, or web links for publicly available datasets
- A description of any restrictions on data availability
- For clinical datasets or third party data, please ensure that the statement adheres to our policy

All data generated in this study have been deposited in the Figshare database under accession code https://doi.org/10.6084/m9.figshare.31843885. All raw data are provided in the Supplementary data and Source Data files.

Research involving human participants, their data, or biological material

Policy information about studies with human participants or human data. See also policy information about sex, gender (identity/presentation), and sexual orientation and race, ethnicity and racism.

Reporting on sex and gender	The study used serum samples from six healthy adult volunteers, consisting of three males and three females (self-reported). Sex was recorded to ensure balanced representation; no sex-based analyses were performed.
Reporting on race, ethnicity, or other socially relevant groupings	Race, ethnicity, and other socially relevant groupings were not collected as they were not relevant to the study objectives and were not required for the analyses performed.
Population characteristics	All participants were healthy adults with no reported chronic illnesses, immunological conditions, or acute infections at the time of sampling. No vulnerable populations were included.
Recruitment	Participants were healthy volunteers recruited through the University College London (UCL) community. Participation was voluntary, and individuals provided written informed consent. No incentives were provided apart from standard, optional travel reimbursement.
Ethics oversight	All human sample collection and use were conducted under approval from the UCL Research Ethics Committee (Protocol 3078/001). All procedures complied with institutional and national ethical guidelines.

Note that full information on the approval of the study protocol must also be provided in the manuscript.

Field-specific reporting

Please select the one below that is the best fit for your research. If you are not sure, read the appropriate sections before making your selection.

☒ Life sciences ☐ Behavioural & social sciences ☐ Ecological, evolutionary & environmental sciences

For a reference copy of the document with all sections, see nature.com/documents/nr-reporting-summary-flat.pdf

Life sciences study design

All studies must disclose on these points even when the disclosure is negative.

Sample size	Sample sizes were based on power calculations made with data from previous vaccination experiments performed in our lab. Data generated, due to the small sample size, were compared using non-parametric tests. Mann-Whitney was used when 2 groups were compared while Kruskal-Wallis was used for multiple comparison using the mean rank of the unvaccinated group as negative control.<br>For vaccination only experiments (array protein-beads screening), we measured the antibody response mounted upon immunisation including 5 mice/group. Our primary outcome was to measure and compare IgG levels generated in 2 conditions (eg. vaccine vs adjuvant alone). The sample size of 5 mice/group has been calculated using serum deposition data, assuming an average of 5 x10+E03 MFI with 2x10+E03 standard deviation in order to measure at least 5-fold increase of MFI in vaccinated samples with 80% power and 0.05 probability of error.<br>For vaccination followed by challenge experiments, we measured protection induced by vaccine and compared it to unvaccinated controls including 6 mice/group. Our primary outcome was to measure and compare CFU number between 2 conditions (vaccine vs adjuvant alone). The sample size of 6 mice/group has been calculated assuming an average of 1 x10+EO6 CFU with 5 x10+E05 standard deviation in order measure at least 90% decrease of CFUs number in vaccinated samples with 85% power and 0.05 probability of error.<br>For the survival study A group size of 10 mice per condition was considered sufficient based on expected survival outcomes. Previous studies and pilot data indicate that >90% of the adjuvant-only control mice succumb within 72 hours, whereas vaccination is expected to confer substantial protection. Using a two-sided log-rank test, this sample size provides over 90% power to detect a significant difference in survival between vaccinated and control groups at a significance level of $\alpha = 0.05$, while minimizing animal use in line with the 3Rs principles.
Data exclusions	Due to the nature and length of the study, a minor proportion of the mice died of natural causes, sometimes before they received any treatment and therefore they were excluded from the analysis. For this reason there are some groups with 5 instead of 6 mice, or 4 instead of 5 mice.
Replication	We replicated the pilot beads-antigen immunisation reshuffling the antigen pools to confirm the validity of the method. We have also performed the full screening with a higher dose of antigens to assess and confirm the quality of the response for the selected antigens. All the other animal immunisations and challenge have not been repeated In line with the 3Rs principles. Instead, we conducted careful a priori sample size calculations to ensure that each experiment was sufficiently powered while minimizing animal use.
Randomization	No randomisation has been applied.
Blinding	No blinding has been applied

Reporting for specific materials, systems and methods

We require information from authors about some types of materials, experimental systems and methods used in many studies. Here, indicate whether each material, system or method listed is relevant to your study. If you are not sure if a list item applies to your research, read the appropriate section before selecting a response.

Materials & experimental systems

n/a	Involved in the study
☐	☒ Antibodies
☒	☐ Eukaryotic cell lines
☒	☐ Palaeontology and archaeology
☐	☒ Animals and other organisms
☒	☐ Clinical data
☒	☐ Dual use research of concern
☒	☐ Plants

Methods

n/a	Involved in the study
☒	☐ ChIP-seq
☐	☒ Flow cytometry
☒	☐ MRI-based neuroimaging

Antibodies

Antibodies used	Anti-mouse IgG AF488 conjugated (A11001, lot 1858182, Thermoscientific), Antimouse IgG HRP conjugate (ab6789; lot GR250300-9, Abcam), anti-mouse IgG-APC (405308, lot B282013, BioLegend), Alexafluor546-conjugated anti-rabbit IgG (A11010, lot 1811305, ThermoFisher), Biotin labelled anti-mouse IgG (405303, lot B253219, Biolegend), AF647 conjugated streptavidin (S21374, lot 2329429, ThermoFisher), Omniserum (2438, lot POM1E1, Statens serum institut).
Validation	*Describe the validation of each primary antibody for the species and application, noting any validation statements on the manufacturer's website, relevant citations, antibody profiles in online databases, or data provided in the manuscript.*

Animals and other research organisms

Policy information about studies involving animals; ARRIVE guidelines recommended for reporting animal research, and Sex and Gender in Research

Laboratory animals	CD1 outbred mice
Wild animals	The study do not involve live animals
Reporting on sex	Only female mice have been used. This is because there are several previous publication indicating differential susceptibility to pneumococcal infection in male and female mice.
Field-collected samples	The study did not involve samples collected form the field
Ethics oversight	Animal research have been conducted at UCL Biological services animal unit working on the UK Home Office approved project license PP3031297

Note that full information on the approval of the study protocol must also be provided in the manuscript.

Plants

Seed stocks	N/A
Novel plant genotypes	N/A
Authentication	N/A

Flow Cytometry

Plots

Confirm that:

- [x] The axis labels state the marker and fluorochrome used (e.g. CD4-FITC).
- [x] The axis scales are clearly visible. Include numbers along axes only for bottom left plot of group (a 'group' is an analysis of identical markers).
- [ ] All plots are contour plots with outliers or pseudocolor plots.
- [ ] A numerical value for number of cells or percentage (with statistics) is provided.

Methodology

Sample preparation	Pneumococcal cells from different strains have been incubated with mouse vaccinated sera and surface binding was revealed using FITC fluorescent anti mouse IgG antibody. A single fluorescent marker is used.
Instrument	BD FACS Verse
Software	FlowJo
Cell population abundance	Only bacteria are present in the samples tested, therefore their abundance would be 100%
Gating strategy	No gating was necessary since only bacteria were used in the analysis

- [ ] Tick this box to confirm that a figure exemplifying the gating strategy is provided in the Supplementary Information.
