# Supplementary material for: High-throughput antigen discovery using Functional Genomic Vaccinology (FGV) identifies protective Streptococcus pneumoniae vaccine candidates
Source: Nat Commun. 2026 Jul 21;17:8867. doi: 10.1038/s41467-026-75848-2 (PMC13500534; doi:10.1038/s41467-026-75848-2)
Supplement: Supplementary file 4 [file 41467_2026_75848_MOESM9_ESM.pdf]

nature portfolio

Peer Review File

High-throughput antigen discovery using Functional Genomic Vaccinology (FGV) identifies protective Streptococcus pneumoniae vaccine candidates

Corresponding Author: Dr Giuseppe Ercoli

This file contains all reviewer reports in order by version, followed by all author rebuttals in order by version.

Version 1:

Reviewer comments:

Reviewer #1

(Remarks to the Author)
In this manuscript by Ercoli et al the authors have developed a new high-throughput platform for antigen discovery of bacterial vaccine candidates, Functional Genomic Vaccinology (FGV). They target a new pneumococcal vaccine and identify 253 conserved pneumococcal proteins of which 222 were expressed in vitro and then incorporated into a protein microarray and coupled to magnetic beads for mouse vaccination. With the protein array, about 40% of the proteins gave a significant IgG response. Using human sera, they selected 22 candidates that were further studied for serological and Th17 responses. 4 antigens were combined and used for vaccination and gave protection in models of pneumococcal pneumonia and sepsis. After intraperitoneal or intranasal pneumococcal challenge, the survival was around 50%. Epitope mapping revealed potential protective sites for each protein. The authors suggest that FGV could be used as a scalable platform for bacterial vaccine discovery.

This is an interesting approach with the possibility for new vaccine discoveries. Particularly, the IL17 data represent a novel tool and are important.

Major comments:

A protein microarray of 253 proteins was developed using reference genome data from 2048 conserved genes across 616 pneumococcal strains. How were the 616 strains selected? How representative are they of the pneumococcal population including more than 107 serotypes and many genotypes?

Healthy human sera (line 95) were used to probe the array. How many sera were used and had these individuals had pneumococcal infections or were vaccinated against pneumococcal infections?

To develop the microarray platform, in total, 268 (104 IgG response+127 conserved+37 virulence associated) pneumococcal proteins were included that were then reduced to 253 depending on further validation using healthy human sera (had these individuals had a prior pneumococcal infection or were vaccinated against pneumococcal infections?), IVIG, and mouse sera from colonized mice. For this platform to be useful for other bacterial species, can you outline what would be the general process for selection of the bacterial antigens to be included?

In Figure 1C, it is not clear how the 37 beads-linked antigens were selected and how many were included. Also, were the mice vaccinated 3 times with either 50, 100 or 400 ng/antigen? As is stated in the figure, you would think that the first immunization was with 50 then with 100 and then with 400, please rephrase to clarify.

Why was intraperitoneal immunization selected and not intranasal or subcutaneous/ intramuscular immunization? Different adjuvants were used in the in vivo experiments: Addavax or Alum. Why did the authors use different adjuvants?

Did the authors, using the FGV platform, identify previously known pneumococcal proteins that have been suggested as major vaccine candidates? Which proteins, all or only a few?

In Figure 2D, the protein PspC10071 4#59 showed a much higher MFI for the low dose as compared to the high dose. Do you have any explanation for this?

In Figure 3, they used pooled human IgG and state natural colonization. What do they mean here with natural colonization?

On line 239 it is stated that 24 proteins were selected for expression as recombinant proteins, and on line 264, 22 proteins. Clarify why it is only 22, ie which ones were excluded.

Figure 4. How many times were the mice vaccinated with the different proteins and with what dose? What was the dose for the intranasal challenge? Do you have survival data after the intranasal challenge of immunized mice for the different proteins?

In Figure 4A IgG levels to recombinant enolase (SP_1128) are shown to be very high. In Figure 4B enolase specific IgG deposition to pneumococci having enolase on its surface is relatively low. One possible explanation to this difference might be that enolase forms an octameric complex on the bacterial surface. It would be important to show whether the generated antibodies primarily recognize linear epitopes that are localized to the interacting regions within the enolase octameric complex. This should be possible to demonstrate by epitope mapping of the enolase octamer and not only with the monomer in a revised Figure 5.

Figure 4B. SP1946 has a high IgG deposition. You did not include this protein in figure 4E. Why?

Figure 4D. Why do you use different colors for the selected proteins? Maybe just use one color for the selected proteins and grey for the non-selected proteins and then state this in the figure legend.

In Figure 4E CFU in blood and lungs of mice vaccinated with 8 individual protein antigen is shown. What was the dose, infection route, and time point for sampling? What about survival? Is the experiment set up the same or different from Figure 7?

In Figure 6C, the capability of antibodies, induced by vaccination with the four selected antigens to bind to different pneumococcal strains, is shown by confocal microscopy. The images are not very convincing. Quantification of antibody-binding, IgG-deposition to intact pneumococcal strains, is important to include. An ideal negative control would be a mutant strain lacking these four antigens but might be difficult to create if some of them represent essential proteins. However, it should be possible to delete at least SP_1128, and SP_0368 and see whether IgG deposition, using vaccinated mouse serum, gets reduced in this double mutant.

In Figure 7, you show protection in mice after immunization intraperitoneally with 4 combined proteins and get around 50% protection after IP (sepsis model, 104 bacteria) and IN (pneumonia model, 2x107 bacteria) challenge, which is good but not great and there was no complete clearance from the blood or spleen in any of the 10 infected mice. How many times did you repeat this experiment? It should have been done at least 2 times. Did you study survival for the individual proteins? A repetition of the IP challenge experiment using the same set up, but prolonging the time to sacrifice, to determine whether the vaccine can generate sterility in blood and spleen would be important. Did you also study protection against colonization?

Table 1 needs to be revised and updated. Please,find below comments on different proteins:

SP_0027 should be named RPPK, and not PrsA which is a lipoprotein and therefore not cytosolic.

SP0368 (Eng) is a 1771 aa long protein that has one C-terminal cell wall anchor sequence but also a trans-membrane sequence. It is present in pneumococcal membrane vesicles, suggesting that it can be membrane associated. Whether or not some part of the Eng protein is also cell wall associated is not known. SP0368c is not the full protein, but the 76 KDa expression product from the C-terminal portion of the gene, that in the living cell, at least partially, must be localized in the bacterial cytosol and not exposed on the bacterial cell surface. The exact prediction of where the trans-membrane motif is localized is needed to estimate the potential surface exposure of the SP0-368c region. SP0368c did not elicit IgG antibodies that deposited on the pneumococcal surface, but a strong IL-17 release. The entire SP0368 protein had perhaps been a better choice as a vaccine candidate than a C-terminal fragment.

SP_1882 has been predicted to be a membrane bound protein since it has a trans-membrane motif and is also present in membrane vesicles. It is annotated as a cytosolic protein in Table 1.

SP_1083 is probably a cytosolic transcription factor protein (SpxR) and likely not a trans-membrane transporter protein as given in Table 1.

SP_1479 contains a trans-membrane motif and is present in membrane vesicles. It is therefore likely not a cytosolic protein as given in Table 1. Its role as a peptidoglycan deacetylase suggests that at least the enzymatic portion of the protein could be exposed to the surface.

SP_ 0992 is a highly interesting potential vaccine target. This DNA-binding protein is called RocS and is a driver in chromosome segregation and nucleoid protection (Mercy et al Nature Microbiology, 2019). It is highly exposed on the pneumococcal surface as shown in Figure 4B suggesting that it is a transmembrane protein. It is not detected in membrane

vesicles suggesting that it is spatially localized in the membrane and/or produced at low levels. This is likely an essential protein.

SP_1518 is an essential trans-membrane protein. Its enzymatic region must be exposed on the outer leaflet of the membrane since it acts as an endolytic murein transglycosylase.

SP_0498b is an expressed part (indicate start and stop of this region) of SP_0498 that is a cell wall anchored surface protein not an exported lipoprotein.

SP_0648 a and c represent different parts of SP_0648 (indicate start and stop for these protein parts. SP_0648 is a typical cell wall-anchored surface exposed protein.

SP_1881 is an oligopeptide binding lipoprotein in an ABC transporter complex and most likely not secreted.

Reviewer #2

(Remarks to the Author)
This study has several areas of high impact. This work would be suitable for a commentary to set the stage.

Impact of this study on the process of vaccine discovery: Vaccine design has undergone several generational improvements over recent time. The fundamental new process, called 'reverse vaccinology', was initiated by whole genome sequencing and advanced through monoclonal antibody development for protective epitopes and now applies structure-based design with Alpha Fold. This manuscript describes the application of these technologies in iterative sequence, a process they call 'Functional Genomic Vaccinology', to find vaccine-suitable protein antigens against pneumococcal infection.

Medical impact of this study: The target of this study is the pneumococcus, which is a major highly invasive pathogen across the entire age spectrum. Many capsule based vaccines are currently in use worldwide but suffer from vaccine escape due to capsule switching to serotypes not in the vaccine formulations. This places a finite number of <30 of the >100 serotypes into any given vaccine formulation and therefore a gap in vaccine coverage that can't be overcome with existing approaches. The alternative has been to find protein antigens that are more universal. This search has been ongoing for decades with varied success leading to a small number of virulence determinants showing promise. However, further development has been difficult due to lack of interest at the big pharma level given the success of current vaccines. One of the important implications of the current study is the hope that a multi-component protein vaccine, as described in this work, will finally be produced and tested at large scale and successfully prevent pneumococcal infection more broadly.

Comments:
1) The structure of the investigation is clearly presented as is the reasoning behind their choices at the various stages of the willowing down of candidates from one step to the next. The final result is a vaccine comprised of a combination of 4 proteins of undisclosed function that together show protective efficacy in rodent models of pneumococcal sepsis and colonization. This study can serve as an excellent example of the application of what is currently the most advanced reverse vaccinology approach to vaccine design.

2) The investigative team is world class. The methods are clearly described and the attention to statistical rigor is outstanding. The conclusions are supported by the data.

3) Why is it that the final 4 proteins in the vaccine are not discussed as to potential function?

4) Given that the study is one of the first of a new generation of methods for vaccine discovery, it is important to reveal the limitations of each step in the process. There are several important assumptions that are glossed over.
a) Immunogenicity of the initial protein array. It is important to point out that strong immunogenicity does not equate with protective activity. Using both high and low antigenicity proteins in the next step is wise but it is not well described how they picked a variety from the pile.
b) Using conservation of sequence as a criterion for a vaccine has limitations. Three D structure/shape is more important than sequence in determining antibody binding. The use of covalescent sera as a screen can address this but the authors could more clearly discuss what is lost using sequence over serum binding. This will be more important in the future as Alpha Fold will show conservations of shape currently being missed.
c) After Step 2, the authors made the arbitrary decision to discard a set of major virulence determinants from moving forward. This is a serious point of controversy. This unfortunately divorces their final vaccine candidate from the extensive experience in the field for no apparent reason. Several of these previously discovered candidates might be very important additions to the final list of 4 they generated. An opportunity has been lost in this decision and it would be very important to add at least some of these previous candidates as positive controls in the mouse vaccination step to compare against the quadrivalent candidate they finally chose.
d) The authors indicate that Th17 activity is a positive result they are in favor of. This is a point of controversy in the field and has been a point of exclusion in the strategies of several companies in choosing their candidates. This should be discussed as pro and con.

Version 2:

Reviewer comments:

Reviewer #1

(Remarks to the Author)
The authors have carefully responded to my comments and revised the manuscript accordingly.

Reviewer #2

(Remarks to the Author)

Open Access This Peer Review File is licensed under a Creative Commons Attribution 4.0 International License, which permits use, sharing, adaptation, distribution and reproduction in any medium or format, as long as you give appropriate credit to the original author(s) and the source, provide a link to the Creative Commons license, and indicate if changes were made.
In cases where reviewers are anonymous, credit should be given to 'Anonymous Referee' and the source.
The images or other third party material in this Peer Review File are included in the article's Creative Commons license, unless indicated otherwise in a credit line to the material. If material is not included in the article's Creative Commons license and your intended use is not permitted by statutory regulation or exceeds the permitted use, you will need to obtain permission directly from the copyright holder.
To view a copy of this license, visit https://creativecommons.org/licenses/by/4.0/

Dear Editors

Thank you for the reviews and the opportunity to resubmit our manuscript after revisions. Overall, we have found the reviews very helpful, and we feel having responded to the points made this will improve the manuscript substantially. A point-by-point answer to their comments is outlined below (in red):

REVIEWER COMMENTS

Reviewer #1 (Remarks to the Author):

In this manuscript by Ercoli et al the authors have developed a new high-throughput platform for antigen discovery of bacterial vaccine candidates, Functional Genomic Vaccinology (FGV). They target a new pneumococcal vaccine and identify 253 conserved pneumococcal proteins of which 222 were expressed in vitro and then incorporated into a protein microarray and coupled to magnetic beads for mouse vaccination. With the protein array, about 40% of the proteins gave a significant IgG response. Using human sera, they selected 22 candidates that were further studied for serological and Th17 responses. 4 antigens were combined and used for vaccination and gave protection in models of pneumococcal pneumonia and sepsis. After intraperitoneal or intranasal pneumococcal challenge, the survival was around 50%. Epitope mapping revealed potential protective sites for each protein. The authors suggest that FGV could be used as a scalable platform for bacterial vaccine discovery.

This is an interesting approach with the possibility for new vaccine discoveries. Particularly, the IL17 data represent a novel tool and are important.

Major comments:

1. A protein microarray of 253 proteins was developed using reference genome data from 2048 conserved genes across 616 pneumococcal strains. How were the 616 strains selected? How representative are they of the pneumococcal population including more than 107 serotypes and many genotypes?

The 616 isolates (133 from 2001, 203 from 2004 and 280 from 2007) used in this analysis were sequenced for a US study evaluating the impact of the 7-valent pneumococcal conjugate vaccine (see reference below). Hence these strains are representative of circulating *S. pneumoniae* strains in a Western population spanning the point at which PCV7 was introduced, and therefore include the increased strain diversity following the introduction of the vaccine. These strains belong to 15 sequence clusters and 31 of the existing 107 capsule serotypes, representing a wide range of the common strains. We have inserted text and reference (24) on this coverage in **lines *92-93*** of the resubmitted manuscript.

24) Croucher NJ, Finkelstein JA, Pelton SI, Mitchell PK, Lee GM, Parkhill J, Bentley SD, Hanage WP, Lipsitch M. Population genomics of post-vaccine changes in pneumococcal epidemiology. Nat Genet. 2013 Jun;45(6):656-63. doi: 10.1038/ng.2625. Epub 2013 May 5. PMID: 23644493; PMCID: PMC3725542.

Healthy human sera (line 95) were used to probe the array. How many sera were used and

had these individuals had pneumococcal infections or were vaccinated against pneumococcal infections?

Line 126: 6 sera from young healthy humans (unvaccinated, no clear history of proven pneumococcal infection) and two sets of IVIG (pooled immunoglobulin from US) were tested. This has been added to the text in **lines 96-99**.

To develop the microarray platform, in total, 268 (104 IgG response+127 conserved+37 virulence associated) pneumococcal proteins were included that were then reduced to 253 depending on further validation using healthy human sera (had these individuals had a prior pneumococcal infection or were vaccinated against pneumococcal infections?) (this point is answered above), IVIG, and mouse sera from colonized mice. For this platform to be useful for other bacterial species, can you outline what would be the general process for selection of the bacterial antigens to be included?

The background rationale for using healthy human sera to validate the array was that each individual will have a significant antibody response to multiple pneumococcal protein antigens induced by prior natural colonisation events. This concept has been added to the text (**lines 96-99** and ***reference 25, lines 638-641***). The validation step is not essential; it just improves the quality of the array as it identifies the antigens to which humans often make a response. We have now included text on how the approach could be made generalisable for other pathogens (discussion ***lines 500-513***) and an extra figure (**Fig.8**) describing the FGV pipeline.

25) Wilson, R. *et al.* Naturally Acquired Human Immunity to Pneumococcus Is Dependent on Antibody to Protein Antigens. *PLoS Pathog* **13**, e1006137 (2017). https://doi.org/10.1371/journal.ppat.1006137

In Figure 1C, it is not clear how the 37 beads-linked antigens were selected and how many were included. Also, were the mice vaccinated 3 times with either 50, 100 or 400 ng/antigen? As is stated in the figure, you would think that the first immunization was with 50 then with 100 and then with 400, please rephrase to clarify.

The 37 antigens were randomly selected, and we have now made this clear in the text and legend (***line 150-152 and Figure 1 legend***). All these antigens were included in the preliminary dose testing study using 9-10 antigen per mouse group (n=5), and 4 groups of mice per dose tested (as stated in Fig. 1 legend). Yes, the reviewer is correct - separate mouse groups were vaccinated 3 times with either 50, 100 or 400 ng of protein per antigen; we have altered the figure legend to make this clearer (***lines 150-152 figure 1 legend***).

Why was intraperitoneal immunization selected and not intranasal or subcutaneous/ intramuscular immunization? Different adjuvants were used in the in vivo experiments: Addavax or Alum. Why did the authors use different adjuvants?

IP testing was used because this route gives robust reproducible responses and was the simplest and quickest injection route possible – this was essential to allow the high number of immunisations to be performed rapidly and consistently. In addition, the IP route allows injection of the higher volumes (200 µl) needed to test multiple proteins simultaneously; this volume is higher than allowed for intranasal and intramuscular vaccinations under the terms of our animal licence.

We selected oil-in-water (O/W) emulsions (Addavax) for the bead-linked protein vaccination study and Alum (Alhydrogel) for the soluble recombinant proteins based on the following justifications:

Bead-Linked Proteins: O/W emulsions were chosen to optimize the delivery of particulate antigens as this adjuvant has previously been shown to induce robust antibody response to multicomponent antigens.

Recombinant Proteins: Alum was selected for soluble recombinant proteins due to its superior ability to adsorb and stabilize antigens. By creating a localized concentration of protein, alum enhances uptake by antigen-presenting cells and reliably triggers the high humoral (antibody-mediated) titers established as the clinical gold standard for subunit vaccines.

Text in the methods has been amended addressing these points in **lines 673-677**, and the references below have been also added.

62) Hernandez-Davies, J. E. *et al.* Magnitude and breadth of antibody cross-reactivity induced by recombinant influenza hemagglutinin trimer vaccine is enhanced by combination adjuvants. *Scientific Reports* **12**, 9198 (2022). https://doi.org/10.1038/s41598-022-12727-y

63) Ramirez, A. *et al.* Co-Delivery of Multiple Toll-Like Receptor Agonists and Avian Influenza Hemagglutinin on Protein Nanoparticles Enhances Vaccine Immunogenicity and Efficacy. *Adv Healthc Mater* **14**, e2404335 (2025).

Did the authors, using the FGV platform, identify previously known pneumococcal proteins that have been suggested as major vaccine candidates? Which proteins, all or only a few?

Most of the antigens previously used as vaccines have been identified by the screening such as: PspA (SP_0017), PspC (SP_2190), PsaA (SP_1650), StkP (SP_1732), PiuA (SP_1872). The exceptions were the previous potential vaccine antigens Ply (SP_1923) and PiaA (SP_1032) which were not identified by the screening. Other previous vaccine antigens were not included in our selection process for the array (eg PhtD SP_1003). We have now added text to the discussion on this (**lines *458-461***).

In Figure 2D, the protein PspC10071 4#59 showed a much higher MFI for the low dose as compared to the high dose. Do you have any explanation for this?

We do not have a clear explanation; we speculate that the high-dose antigen exposure may induce immune tolerance or exhaustion, or result in or epitope masking for that specific protein. Due to space, we have not added text to the resubmitted text on this point.

In Figure 3, they used pooled human IgG and state natural colonization. What do they mean here with natural colonization?

"Natural colonization" refers to asymptomatic pneumococcal carriage that we now know induces significant systemic antibody responses even in the absence of clinical disease[23-25]; human immunoglobulin will contain these pooled antibody responses for the population used as donors.

The text in the beginning of the results section has been amended to make this clearer (***lines 96-99***).

On line 239 it is stated that 24 proteins were selected for expression as recombinant proteins, and on line 264, 22 proteins. Clarify why it is only 22, ie which ones were excluded.

The 2 proteins excluded (SP_1732 and SP_1012) failed at the recombinant protein purification step. The list of all proteins selected at each step is shown in the supplementary materials. The text has been amended to improve clarity (***lines 246-247***).

Figure 4. How many times were the mice vaccinated with the different proteins and with what dose? What was the dose for the intranasal challenge? Do you have survival data after the intranasal challenge of immunized mice for the different proteins?

Mice were vaccinated with 3 doses of recombinant proteins and the dose used was 10 µg per antigen per dose (specified in the methods section **lines *678-679, and now added to the figure 4 legend***). Intranasal challenge dose was 1 × 10^7 CFU of pneumococcal strain TIGR4 in a 50 µl suspension (specified in the methods **lines 682-685 and in the legend for Figure 4**). For the experiment described in Figure 4 we only assessed the effect of vaccination on target organ CFU 24 hours after infection; due to the higher severity, our licence for animal work requires us to limit the number of survival experiments we perform and for the screening process described in this part of the study target organ CFU was just as able to identify potential good vaccine candidates. Due to space, we have not added text to the resubmitted text on this point.

In Figure 4A IgG levels to recombinant enolase (SP_1128) are shown to be very high. In Figure 4B enolase specific IgG deposition to pneumococci having enolase on its surface is relatively low. One possible explanation to this difference might be that enolase forms an octameric complex on the bacterial surface. It would be important to show whether the generated antibodies primarily recognize linear epitopes that are localized to the interacting regions within the enolase octameric complex. This should be possible to demonstrate by epitope mapping of the enolase octamer and not only with the monomer in a revised Figure 5.

Yes, we agree with Reviewer 1 that the level of antibodies produced against SP_1128 were very high, but we disagree on considering the binding to the bacterial surface measured by flow cytometry to be low. Binding of IgG to SP_1128 in the flow cytometry assay was relatively high (4th highest of those tested, and the scale used is exponential). We are grateful to the reviewer pointing out that SP_1128 forms an octamer, and we have now included a figure mapping the linear epitopes to this octameric structure (Figure 5). For SP_1128, human IgG recognition is restricted to epitopes exposed on the surface of the assembled octamer; the inter-monomer contact interfaces, which are buried upon oligomerisation, were not recognised by IVIG (discussed in the results, **lines 360-367**).

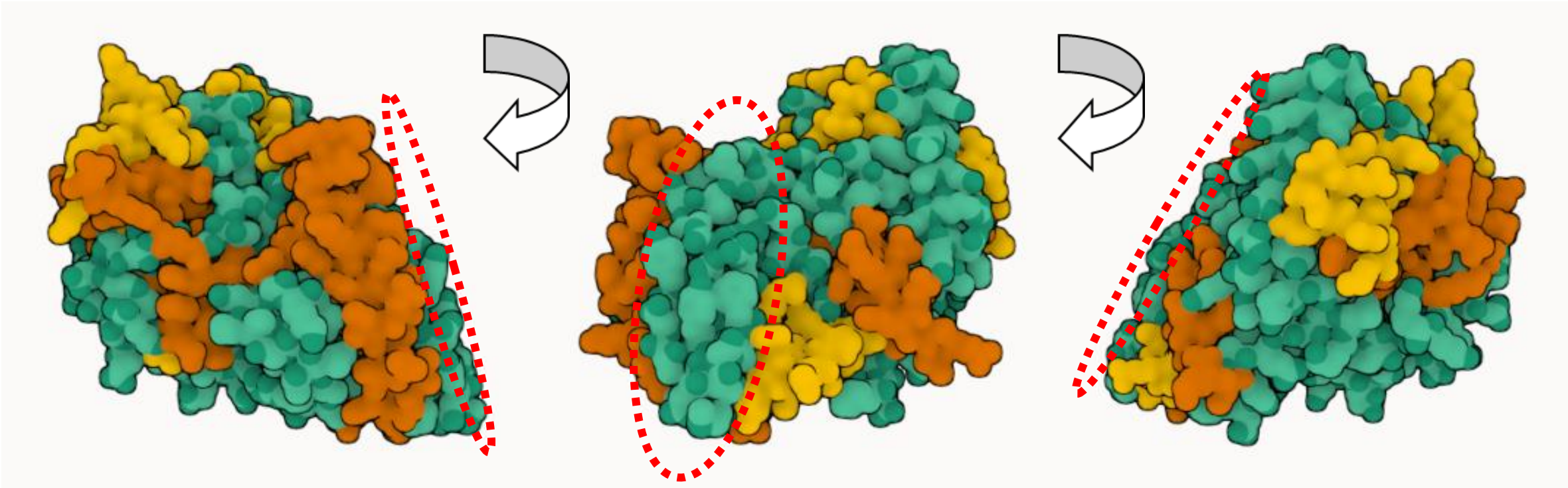

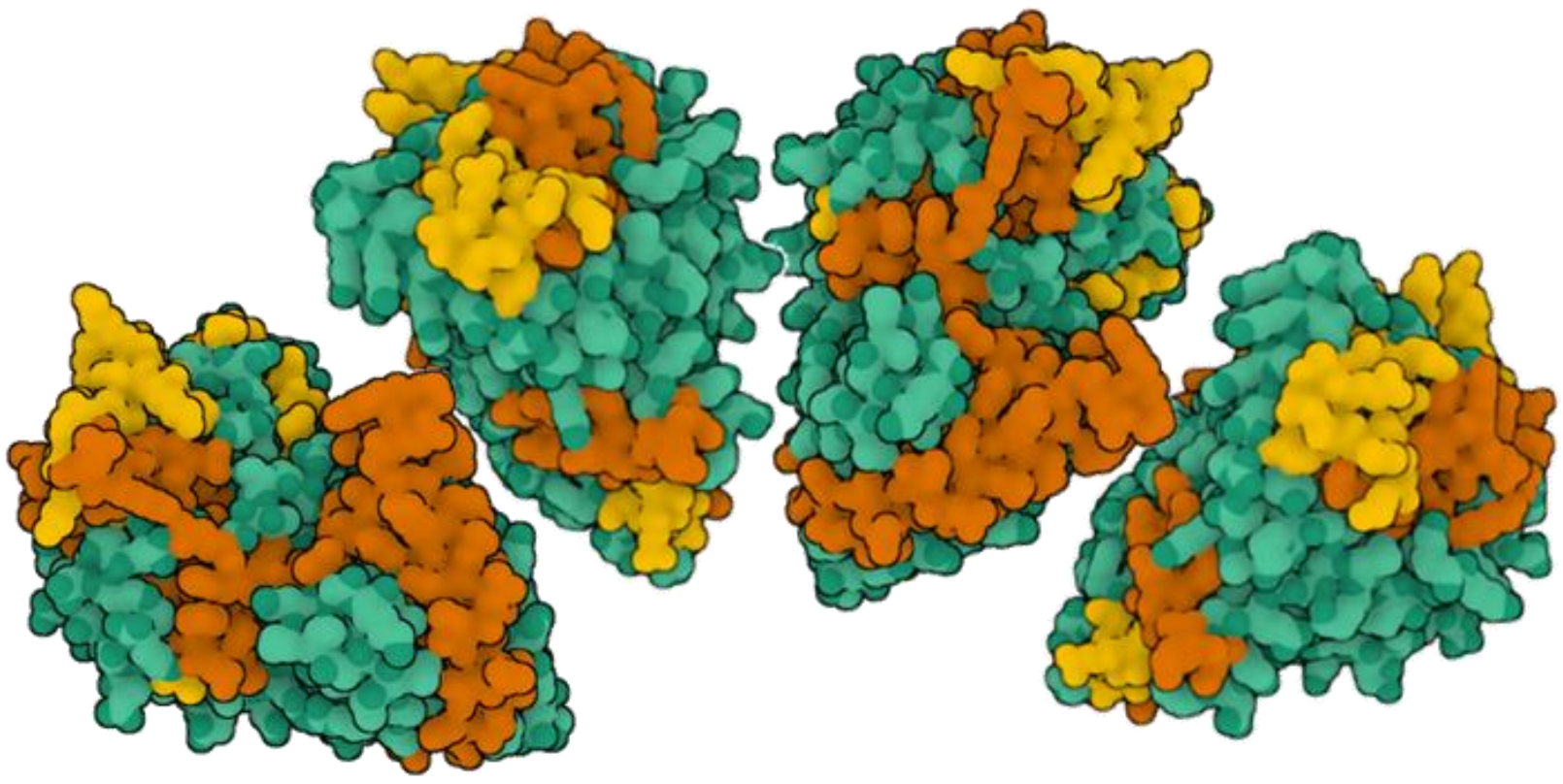

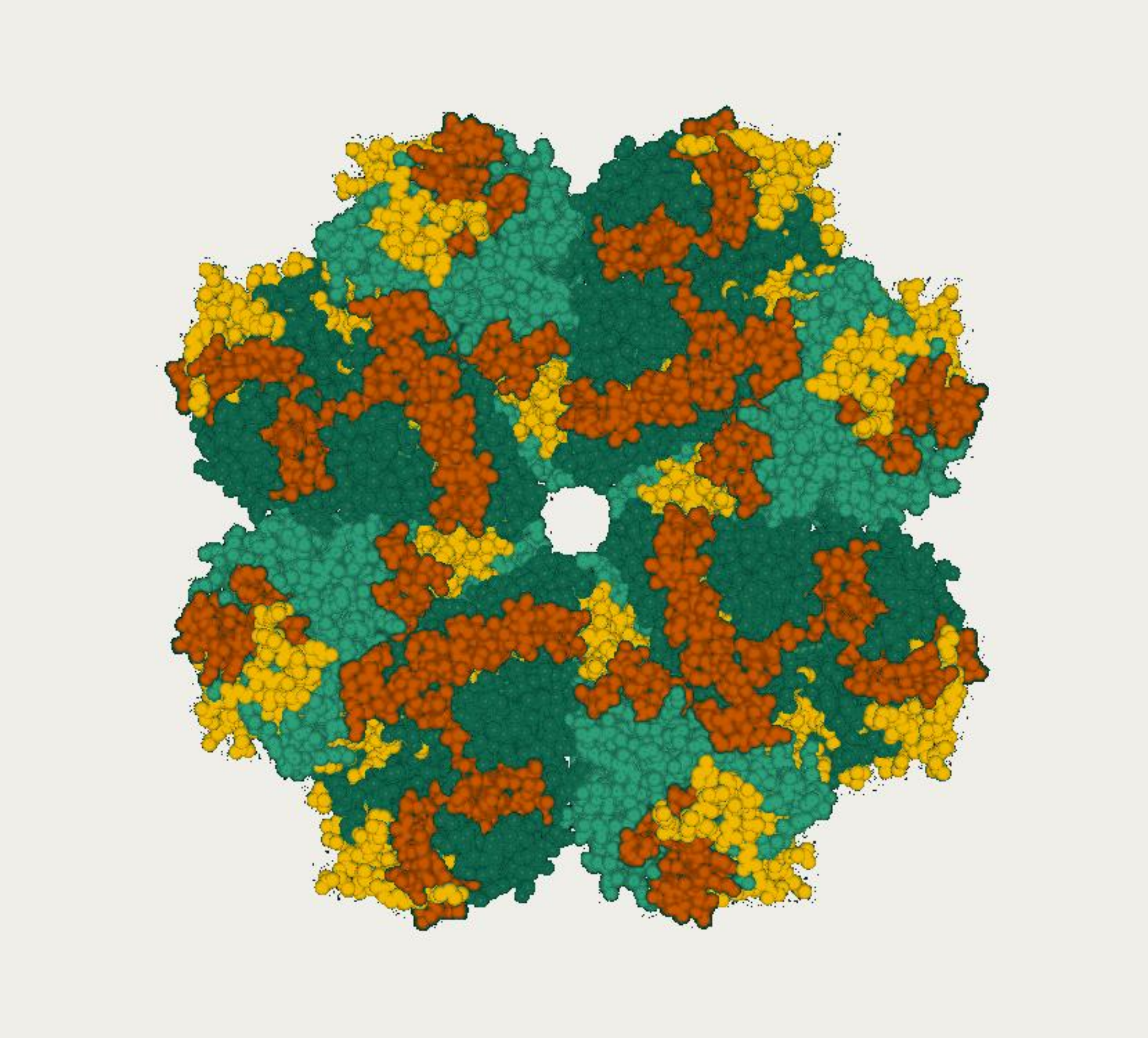

Figure 4B. SP1946 has a high IgG deposition. You did not include this protein in figure 4E. Why?

Despite a strong IgG response generated upon vaccination and a good antibody binding to the bacterial surface, SP_1946 was not selected for protection studies as it only achieved an overall low total antigen score (Fig. 4D), mainly because normal human sera often has very high levels of circulating IgG to this antigen making vaccination unlikely to provide much additional benefit. Furthermore, SP_1946 did not induce Th17 responses and was not predicted to be surface exposed. Due to space we have not added text to the resubmitted text on this point.

Figure 4D. Why do you use different colors for the selected proteins? Maybe just use one color for the selected proteins and grey for the non-selected proteins and then state this in the figure legend.

We apologise for the confusion. We have amended the table to make it easier to interpret, we used just one colour. We used green for the all the positive features in the shortlisted antigens and grey for the positive categories in the not shortlisted **as now described in the legend to Figure 4.**

In Figure 4E CFU in blood and lungs of mice vaccinated with 8 individual protein antigen is

shown. What was the dose, infection route, and time point for sampling? What about survival? Is the experiment set up the same or different from Figure 7?

These details were described in the methods section **(line 678-685)**, but we have now added the vaccination schedule details to the figure legends. Mice were vaccinated IP with 3 doses of recombinant proteins at day 1, 14, and 28. The dose used was 10 µg per antigen per dose. Mice were infected with *S. pneumoniae* on day 52. The schedule was the same for the 24 hours experiment (Fig. 4) and for the survival study tested on the combination vaccine (Fig. 7). Sera samples were collected 3 days before challenge and at the experiment endpoint.

In Figure 6C, the capability of antibodies, induced by vaccination with the four selected antigens to bind to different pneumococcal strains, is shown by confocal microscopy. The images are not very convincing. Quantification of antibody-binding, IgG-deposition to intact pneumococcal strains, is important to include. An ideal negative control would be a mutant strain lacking these four antigens but might be difficult to create if some of them represent essential proteins. However, it should be possible to delete at least SP_1128, and SP_0368 and see whether IgG deposition, using vaccinated mouse serum, gets reduced in this double mutant.

We thank the reviewer for this important comment. We agree that the confocal microscopy images shown in the original version of Figure 6C were primarily qualitative and not optimal for accurately quantifying antibody binding to pneumococcal cells.

To address this concern, we have now included flow cytometry–based quantification of IgG binding to intact pneumococci using sera from vaccinated mice compared with sera from adjuvant-only control animals (**Fig.6c-d**). These experiments were performed for several encapsulated and unencapsulated pneumococcal strains, and the results demonstrate a significant increase in IgG binding to bacterial cells in the vaccinated group. These quantitative data are **now presented in Figure 6 and text (lines 386-387)**.

To further investigate the contribution of individual antigens to antibody binding, we generated single-gene deletion mutants in an unencapsulated TIGR4 strain (to increase IgG binding and thereby make changes for the mutant strains more obvious) for three of the four antigens: SP_0368, SP_0992, and SP_1882. We also tried to obtain a deletion mutant for SP_1128, but this was unsuccessful (as expected as this gene is reported to be essential in several publications).

39) *Liu X, Gallay C, Kjos M, Domenech A, Slager J, van Kessel SP, Knoops K, Sorg RA, Zhang JR, Veening JW. High-throughput CRISPRi phenotyping identifies new essential genes in Streptococcus pneumoniae. Mol Syst Biol. 2017 May 10;13(5):931. doi: 10.15252/msb.20167449. PMID: 28490437; PMCID: PMC5448163.*

40) *Song JH, Ko KS, Lee JY, Baek JY, Oh WS, Yoon HS, Jeong JY, Chun J. Identification of essential genes in Streptococcus pneumoniae by allelic replacement mutagenesis. Mol Cells. 2005 Jun 30;19(3):365-74. PMID: 15995353.*

Using these mutants as controls, we assessed IgG binding to the bacterial surface using sera from vaccinated animals. We observed a significant reduction in IgG binding in the SP_0368 and SP_0992 deletion mutants, indicating that antibodies raised against these antigens contribute measurably to the overall surface-binding activity of the immune sera. In contrast, deletion of SP_1882 did not significantly affect IgG binding under the conditions tested; this antigen though was a strong inducer of Th17 responses (Fig. 4C) (**Added text in lines 387-395** and **606-609**).

These new experiments provide quantitative confirmation of antibody binding to intact pneumococci and help to clarify the contribution of individual antigens to the antibody response elicited by the four-antigen vaccine formulation. The results have now been incorporated into **Figure 6 and in the revised manuscript.**

In Figure 7, you show protection in mice after immunization intraperitoneally with 4 combined proteins and get around 50% protection after IP (sepsis model, 104 bacteria) and IN (pneumonia model, 2x107 bacteria) challenge, which is good but not great and there was no complete clearance from the blood or spleen in any of the 10 infected mice. How many times did you repeat this experiment? It should have been done at least 2 times. Did you study survival for the individual proteins? A repetition of the IP challenge experiment using the same set up, but prolonging the time to sacrifice, to determine whether the vaccine can generate sterility in blood and spleen would be important. Did you also study protection against colonization?

We thank the reviewer for these thoughtful comments. The primary objective of this study was to demonstrate a novel methodology for the rapid identification of potential vaccine antigens, using *S. pneumoniae* as a model organism. The *in vivo* vaccination experiments were therefore intended as proof of concept, to demonstrate that antigens identified through this screening approach can confer measurable protection in established murine infection models. A more extensive evaluation of the individual proteins as vaccine candidates — including repeated survival studies, assessment of sterilizing immunity, and studies of colonization — are planned, but we believe that such investigations fall beyond the scope of the present study and would be better addressed in a dedicated follow-up study focused on the detailed characterization of these antigens.

With regard to the reviewer's specific questions:

(a) Repetition of the experiments.

In the existing manuscript each antigen has shown a degree of protective efficacy in animal models on three occasions; once as a single antigen in the pneumonia model, and then twice as a quadrivalent vaccine once each in a sepsis model and pneumonia model using relatively large group sizes (n = 10 vaccinated animals per group) to ensure sufficient statistical power within each experiment. Hence, we feel the data showing they induce protection is robust.

(b) Level of protection observed, lack of complete bacterial clearance, and protection conferred by individual antigens and against colonisation.

The challenge doses used in the survival experiments were intentionally set at levels that produce uniformly lethal infection in control animals. Under such stringent conditions, achieving sterilizing immunity is unlikely and significantly delaying fatal infection represents significant protective immunity (especially for the IP sepsis model which leads to rapid systemic dissemination of bacteria). In the intranasal pneumonia model — which more closely reflects the natural route of infection — we observed complete clearance from the bloodstream in 5 of the 6 surviving vaccinated mice, indicating that vaccination was able to effectively limit systemic dissemination following pulmonary infection. Taken together, the results demonstrate that antigens identified using our screening methodology can confer measurable protection in stringent murine models of pneumococcal disease, supporting the validity of this approach for the discovery of novel vaccine candidates. While not fully protective, the level of protective efficacy is notable considering that all four antigens are conserved proteins that, to our knowledge, have not previously been identified or predicted

as vaccine candidates. Furthermore, this level of protection is consistent with that typically previously reported for individual or small combinations of pneumococcal protein antigens evaluated in stringent murine challenge models (***see references below***). These data form the starting basis for more detailed vaccination studies to fully define the most effective protein antigen combinations for generating serotype-independent protection against different aspects of *S. pneumoniae* infection which requiring an extensive set of experiments that we believe are beyond the scope of the existing manuscript. We agree with the reviewer that these experiments will need to include extending the survival analysis to later time points to assess sterilizing immunity, evaluating protection against nasopharyngeal colonization, and improving protective immunity including testing our antigens in combination with existing know protective antigens. This has now been suggested in the discussion (***lines 559-562***).

Godfroid F, Hermand P, Verlant V, Denoël P, Poolman JT. Preclinical evaluation of the Pht proteins as potential cross-protective pneumococcal vaccine antigens. Infect Immun. 2011 Jan;79(1):238-45. doi: 10.1128/IAI.00378-10. Epub 2010 Oct 18. PMID: 20956575; PMCID: PMC3019885.

Olafsdottir TA, Lingnau K, Nagy E, Jonsdottir I. Novel protein-based pneumococcal vaccines administered with the Th1-promoting adjuvant IC31 induce protective immunity against pneumococcal disease in neonatal mice. Infect Immun. 2012 Jan;80(1):461-8. doi: 10.1128/IAI.05801-11. Epub 2011 Oct 24. PMID: 22025519; PMCID: PMC3255653.

Corsini B, Aguinagalde L, Ruiz S, Domenech M, Yuste J. Vaccination with LytA, LytC, or Pce of Streptococcus pneumoniae Protects against Sepsis by Inducing IgGs That Activate the Complement System. Vaccines (Basel). 2021 Feb 23;9(2):186. doi: 10.3390/vaccines9020186. PMID: 33672306; PMCID: PMC7926378.

Table 1 needs to be revised and updated. Please, find below comments on different proteins:

We thank the reviewer for providing this helpful information and have included all the suggested changes listed below; this has improved the quality of the information provided by this table.

SP_0027 should be named RPPK, and not PrsA.

SP_1882 has been predicted to be a membrane bound protein since it has a trans-membrane motif and is also present in membrane vesicles. It is annotated as a cytosolic protein in Table 1.

SP_1083 is probably a cytosolic transcription factor protein (SpxR) and likely not a trans-membrane transporter protein as given in Table 1.

SP_1479 contains a trans-membrane motif and is present in membrane vesicles. It is therefore likely not a cytosolic protein as given in Table 1. Its role as a peptidoglycan deacetylase suggests that at least the enzymatic portion of the protein could be exposed to the surface.

SP_ 0992 is a highly interesting potential vaccine target. This DNA-binding protein is called RocS and is a driver in chromosome segregation and nucleoid protection (Mercy et al Nature Microbiology, 2019). It is highly exposed on the pneumococcal surface as shown in Figure 4B suggesting that it is a transmembrane protein. It is not detected in membrane vesicles

suggesting that it is spatially localized in the membrane and/or produced at low levels. This is likely an essential protein.

SP_1518 is an essential trans-membrane protein. Its enzymatic region must be exposed on the outer leaflet of the membrane since it acts as an endolytic murein transglycosylase.

SP_0498b is an expressed part (indicate start and stop of this region) of SP_0498 that is a cell wall anchored surface protein not an exported lipoprotein.

SP_0648 a and c represent different parts of SP_0648 a typical cell wall-anchored surface exposed protein. SP_0648 is a typical cell wall-anchored surface exposed protein.

SP_1891 is an oligopeptide binding lipoprotein in an ABC transporter complex and most likely not secreted.

SP0368 (Eng) is a 1771 aa long protein that has one C-terminal cell wall anchor sequence but also a trans-membrane sequence. It is present in pneumococcal membrane vesicles, suggesting that it can be membrane associated. Whether or not some part of the Eng protein is also cell wall associated is not known. SP0368c is not the full protein, but the 76 KDa expression product from the C-terminal portion of the gene, that in the living cell, at least partially, must be localized in the bacterial cytosol and not exposed on the bacterial cell surface. The exact prediction of where the trans-membrane motif is localized is needed to estimate the potential surface exposure of the SP0-368c region. SP0368c did not elicit IgG antibodies that deposited on the pneumococcal surface, but a strong IL-17 release. The entire SP0368 protein had perhaps been a better choice as a vaccine candidate than a C-terminal fragment.

Due to technical constraints of the high-throughput expression and screening platform, proteins longer than approximately 1,000 amino acids could not be tested as full-length constructs and were therefore divided into discrete domains. Accordingly, SP_0368 was represented in the screen by three separate fragments (SP_0368a, SP_0368b, and SP_0368c), all of which were included in the initial antigenicity screening. Among these, SP_0368c emerged as the fragment with the most favourable immunological profile and was therefore selected for downstream characterization.

Sequence analysis indicates that SP_0368 contains a canonical LPXTG cell-wall anchoring motif at the C-terminus, which is included within the SP_0368c fragment. Using the TMHMM 2.0 prediction tool, we did not detect additional transmembrane helices within the SP_0368 sequence (see figure below), suggesting that the protein is most likely anchored to the cell wall through the LPXTG motif rather than integrated into the membrane through multiple transmembrane regions.

FULL LENGTH SP_0368 transmembrane prediction (Red box indicates the SP_0368c portion (c-term))

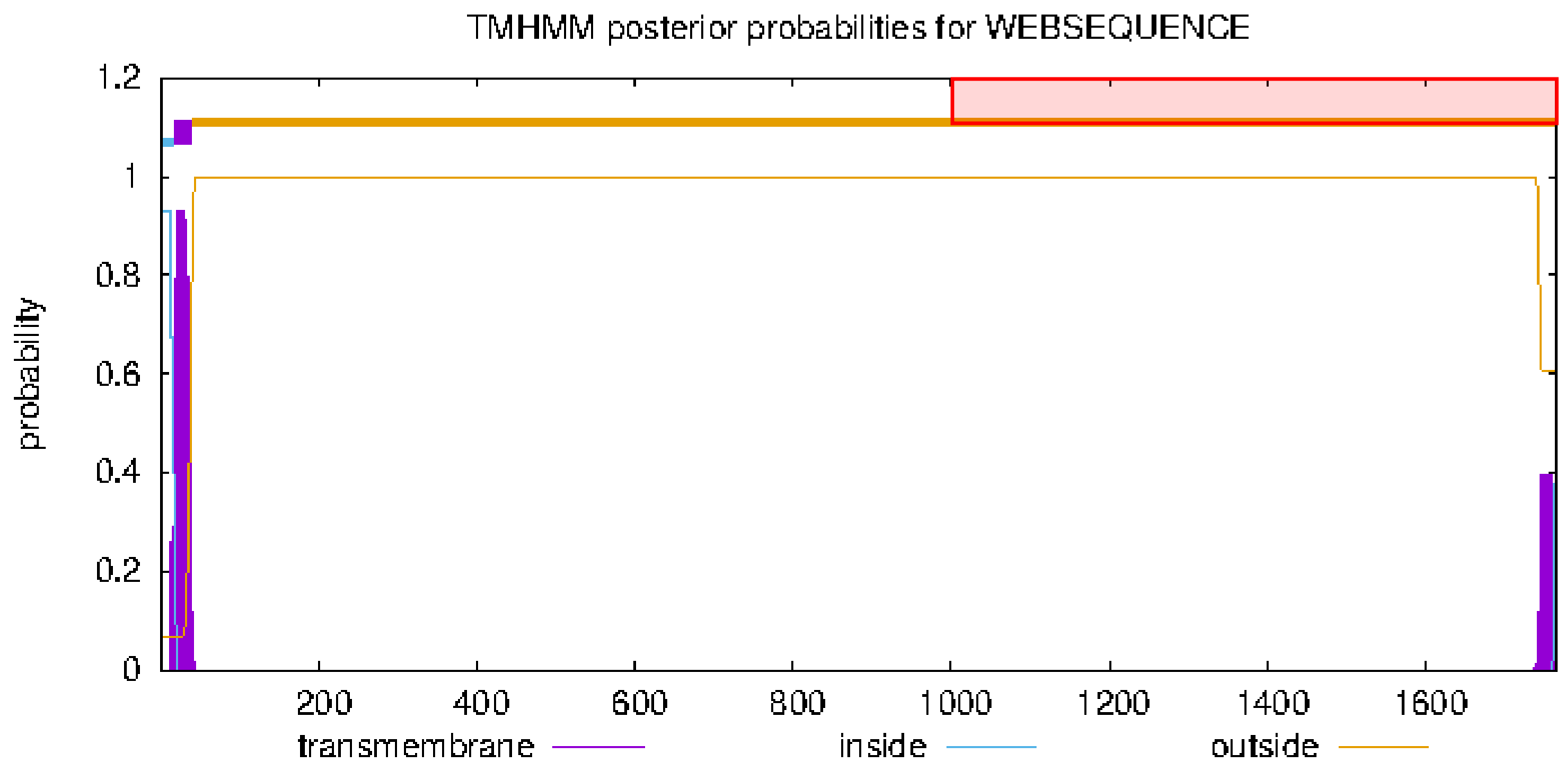

(https://services.healthtech.dtu.dk/services/TMHMM-2.0/)

We acknowledge that antibodies raised against SP_0368c showed limited IgG deposition on the surface of fully encapsulated pneumococci. However, encapsulated pneumococci are known to be difficult targets for antibodies directed against protein antigens due to shielding by the polysaccharide capsule. In the revised manuscript we include new data (**Figure 6D**) showing antibody binding to an unencapsulated pneumococcal strain, with and without deletion of the SP_0368 gene. These experiments demonstrate a specific binding signal attributable to antibodies raised against SP_0368c when the capsule barrier is removed, supporting the notion that this antigen can be accessible on the bacterial surface under appropriate conditions.

Importantly, antigen selection in this study was not based solely on the induction of surface-binding antibodies. A major objective was to identify antigens capable of eliciting cellular immune responses, particularly Th17 responses, which are increasingly recognised as contributing to protection against pneumococcal colonisation and infection. In this context, SP_0368c was notable for inducing a strong IL-17 response despite limited IgG deposition on encapsulated bacteria.

We agree with the reviewer that testing alternative fragments or the full-length SP_0368 protein could potentially reveal constructs with improved antibody accessibility and protective capacity. However, the full-length SP_0368 protein has a size of about 200 KDa, making it challenging to produce as recombinant. Moreover, optimisation of individual antigen constructs was not the primary objective of this study. Rather, the goal was to demonstrate the feasibility of Functional Genomic Vaccinology as a scalable and experimentally driven screening strategy to identify novel pneumococcal vaccine candidates. Further refinement of antigen design, including evaluation of full-length proteins and alternative domain combinations, represents an important next step building on the results obtained using FGV.

Reviewer #2 (Remarks to the Author):

This study has several areas of high impact. This work would be suitable for a commentary to set the stage.

Impact of this study on the process of vaccine discovery: Vaccine design has undergone several generational improvements over recent time. The fundamental new process, called 'reverse vaccinology', was initiated by whole genome sequencing and advanced through monoclonal antibody development for protective epitopes and now applies structure-based design with Alpha Fold. This manuscript describes the application of these technologies in iterative sequence, a process they call 'Functional Genomic Vaccinology', to find vaccine-suitable protein antigens against pneumococcal infection.

Medical impact of this study: The target of this study is the pneumococcus, which is a major highly invasive pathogen across the entire age spectrum. Many capsule based vaccines are currently in use worldwide but suffer from vaccine escape due to capsule switching to serotypes not in the vaccine formulations. This places a finite number of <30 of the >100 serotypes into any given vaccine formulation and therefore a gap in vaccine coverage that can't be overcome with existing approaches. The alternative has been to find protein antigens that are more universal. This search has been ongoing for decades with varied success leading to a small number of virulence determinants showing promise. However, further development has been difficult due to lack of interest at the big pharma level given the success of current vaccines. One of the important implications of the current study is the hope that a multi-component protein vaccine, as described in this work, will finally be produced and tested at large scale and successfully prevent pneumococcal infection more broadly.

Comments:

1) The structure of the investigation is clearly presented as is the reasoning behind their choices at the various stages of the willowing down of candidates from one step to the next. The final result is a vaccine comprised of a combination of 4 proteins of undisclosed function that together show protective efficacy in rodent models of pneumococcal sepsis and colonization. This study can serve as an excellent example of the application of what is currently the most advanced reverse vaccinology approach to vaccine design.

2) The investigative team is world class. The methods are clearly described and the attention to statistical rigor is outstanding. The conclusions are supported by the data.

We thank the reviewer for these positive comments.

3) Why is it that the final 4 proteins in the vaccine are not discussed as to potential function?

The four antigens selected for the final multicomponent vaccine include proteins with limited or poorly defined functional annotation, an outcome that was partly due to the unbiased nature of the FGV approach. A brief overview of their functions (known for only two) was given in the results section of the original submission (***lines 338-344*** in the resubmission text), and is now supported by additional text in the discussion (***lines 548-556***).

4) Given that the study is one of the first of a new generation of methods for vaccine discovery, it is important to reveal the limitations of each step in the process.

The main limitations of the FGV approach are given in several areas within the discussion text e.g. **lines 492-495, 537-539, 548-549, 559-562** and **565-567**.

a) Immunogenicity of the initial protein array. It is important to point out that strong immunogenicity does not equate with protective activity. Using both high and low antigenicity proteins in the next step is wise but it is not well described how they picked a variety from the pile.

We fully agree that immunogenicity alone is not predictive of protective efficacy. For this reason, immunogenicity was not used as a stand-alone selection criterion. Instead, we intentionally advanced antigens displaying a broad range of antibody responses, including proteins with relatively low IgG reactivity, to downstream functional evaluation. This approach was designed to avoid over-selection of highly immunodominant but potentially non-protective antigens. We have added text to the revised manuscript in the discussion on this point (***lines 524-528***).

b) Using conservation of sequence as a criterion for a vaccine has limitations. Three D structure/shape is more important than sequence in determining antibody binding. The use of convalescent sera as a screen can address this but the authors could more clearly discuss what is lost using sequence over serum binding. This will be more important in the future as Alpha Fold will show conservations of shape currently being missed.

We agree that three-dimensional structure and epitope conformation are critical determinants of antibody recognition and that sequence conservation alone may fail to capture structurally conserved protective epitopes. Sequence conservation was therefore used as an initial filtering step to ensure broad strain coverage rather than as a predictor of immunogenicity or protection. The use of human sera (including pooled immunoglobulin preparations) was intended to partially address this limitation by incorporating immune recognition of native antigenic structures. We agree that future integration of structure-based analyses, including AlphaFold-derived structural conservation, represents an important direction for further refinement of the FGV pipeline, and have acknowledged this in the discussion (***lines 565-567***).

c) After Step 2, the authors made the arbitrary decision to discard a set of major virulence determinants from moving forward. This is a serious point of controversy. This unfortunately divorces their final vaccine candidate from the extensive experience in the field for no apparent reason. Several of these previously discovered candidates might be very important additions to the final list of 4 they generated. An opportunity has been lost in this decision and it would be very important to add at least some of these previous candidates as positive controls in the mouse vaccination step to compare against the quadrivalent candidate they finally chose.

Previously studied pneumococcal vaccine candidates were intentionally excluded from downstream testing to allow an unbiased evaluation of novel antigens and to avoid rediscovering established targets. We agree that this decision excludes direct comparison with well-characterised candidates and that some of these proteins may ultimately complement the antigens identified here. Our intention was not to dismiss prior work, but rather to demonstrate the capacity of FGV to expand the antigen discovery space beyond traditional candidates. We have clarified this point in the revised Discussion and explicitly

state that FGV is complementary to, rather than a replacement for, existing candidate-driven strategies (***lines 556-562***).

d) The authors indicate that Th17 activity is a positive result they are in favor of. This is a point of controversy in the field and has been a point of exclusion in the strategies of several companies in choosing their candidates. This should be discussed as pro and con.

We agree that the role of Th17 responses in protective immunity remains an area of active debate and that different vaccine developers have adopted divergent strategies regarding their inclusion. In this study, Th17 responses were evaluated as one component of a broader immune profile, alongside antibody production and opsonisation. We consider Th17 responses as potentially beneficial, particularly in the context of mucosal immunity and protein-based vaccines, but more human data are needed. As suggested by the reviewer, the Discussion has been revised to reflect this (***lines 537-539***).
